# A Phase II, Randomized Study on an Investigational DTPw-HBV/Hib-MenAC Conjugate Vaccine Administered to Infants in Northern Ghana

**DOI:** 10.1371/journal.pone.0002159

**Published:** 2008-05-14

**Authors:** Abraham Hodgson, Abudulai Adams Forgor, Daniel Chandramohan, Zarifah Reed, Fred Binka, Cornelia Bevilacqua, Dominique Boutriau, Brian Greenwood

**Affiliations:** 1 Navrongo Health Research Centre, Ministry of Health, Navrongo, Ghana; 2 London School of Hygiene & Tropical Medicine, London, United Kingdom; 3 World Health Organization, Geneva, Switzerland; 4 University of Ghana, Accra, Ghana; 5 Consultant, Basel, Switzerland; 6 GlaxoSmithKline Biologicals, Rixensart, Belgium; Institute of Child Health, United Kingdom

## Abstract

**Background:**

Combining meningococcal vaccination with routine immunization in infancy may reduce the burden of meningococcal meningitis, especially in the meningitis belt of Africa. We have evaluated the immunogenicity, persistence of immune response, immune memory and safety of an investigational DTPw-HBV/Hib-MenAC conjugate vaccine given to infants in Northern Ghana.

**Methods and Findings:**

In this phase II, double blind, randomized, controlled study, 280 infants were primed with DTPw-HBV/Hib-MenAC or DTPw-HBV/Hib vaccines at 6, 10 and 14 weeks of age. At 12 months of age, children in each group received a challenge dose of serogroup A+C polysaccharides. Antibody responses were assessed pre, and one month-post dose 3 of the priming schedule and pre and 1 month after administration of the challenge dose. One month post-dose 3, 87.8% and 88.2% of subjects in the study group had bactericidal meningococcal serogroup A (SBA-MenA) and meningococcal serogroup C (SBA-MenC) antibody titres ≥1∶8 respectively. Seroprotection/seropositivity rates to the 5 antigens administered in the routine EPI schedule were non-inferior in children in the study group compared to those in the control group. The percentages of subjects in the study group with persisting SBA-MenA titres ≥ 1∶8 or SBA-MenC titres ≥1∶8 at the age of 12 months prior to challenge were significantly higher than in control group (47.7% vs 25.7% and 56.4% vs 5.1% respectively). The administration of 10 μg of serogroup A polysaccharide increased the SBA-MenA GMT by 14.0-fold in the DTPW-HBV/HibMenAC-group compared to a 3.8 fold increase in the control-group. Corresponding fold-increases in SBA-MenC titres following challenge with 10 μg of group C polysaccharide were 18.8 and 1.9 respectively. Reactogenicity following primary vaccination or the administration of the challenge dose was similar in both groups, except for swelling (Grade 3) after primary vaccination which was more frequent in children in the vaccine than in the control group (23.7%; 95%CI [19.6–28.1] of doses vs 14.1%; 95% CI [10.9–17.8] of doses). Fifty-nine SAEs (including 8 deaths), none of them related to vaccination, were reported during the entire study.

**Conclusions:**

Three dose primary vaccination with DTPw-HBV/Hib-MenAC was non-inferior to DTPw-HBV/Hib for the 5 common antigens used in the routine EPI schedule and induced bactericidal antibodies against *Neisseria meningitidis* of serogroups A and C in the majority of infants. Serogroup A and C bactericidal antibody levels had fallen below titres associated with protection in nearly half of the infants by the age of 12 months confirming that a booster dose is required at about that age. An enhanced memory response was shown after polysaccharide challenge. This vaccine could provide protection against 7 important childhood diseases (including meningococcal A and C) and be of particular value in countries of the African meningitis belt.

**Trial Registration:**

Controlled-Trials.com ISRCTN35754083

## Introduction

Meningococcal disease affects up to 1.2 million people worldwide each year with a death toll estimated at around 135, 000 [1]. The highest burden occurs in the “meningitis belt” of sub-Saharan Africa which extends across the Sahel and sub-Sahel from Senegal to Ethiopia. Meningitis epidemics have also been reported in Africa outside the meningitis belt (Morocco, Rwanda, Burundi, Democratic Republic of Congo, Kenya, and Zambia) [2]–[4]. In these regions, an estimated 250, 000 persons become infected each year [5] with a mortality rate averaging 10% but which can reach 30% during epidemics; 10–15% of survivors have neurological sequelae [1], [3], [6], [7]. Children between 3 months and 5 years of age have the highest risk of contracting the disease but during epidemics, older children and young adults are vulnerable [4], [8]. During inter-epidemic years, incidence and the case fatality rate are high among infants [8]. The treatment of choice in Africa during epidemics is parenteral chloramphenicol or third-generation cephalosporins, such as ceftriaxone [9], [10].

In Africa, serogroup A *Neisseria meningitidis* is still responsible for most meningococcal epidemics, while group C meningococci have caused some outbreaks [3]. An outbreak due to serogroup group W135 meningococci occurred recently among Hajj pilgrims [11], and W135 epidemics or outbreaks have also been reported in Niger [12], Burkina Faso [13] and Chad [14]. Cases of W135 disease have now been detected in nearly all countries of the “meningitis belt” including Ghana [7], [15]. A serogroup X outbreak was reported in Niger in 2006 [16].

Polysaccharide vaccines against serogroup A and C meningoccocal infections have existed since the late 1960s and are readily accessible and affordable. Following the recent emergence of the W-135 serogroup in Africa, a trivalent polysaccharide ACW-135 vaccine has been developed and made available at reasonable cost [4], [7]. There is no vaccine available to protect against serogroup X meningococci. Polysaccharide vaccines, used mainly for reactive mass vaccination during epidemics, are poorly immunogenic in children under 2 years of age (except for serogroup A), induce short-lived protection (3–5 years) in children, and have a limited ability to reduce nasopharyngeal carriage and induce herd immunity [7]. Although mass vaccination with polysaccharide vaccines can prevent up to 70% of cases [17], [18] if implemented at the onset of an outbreak such high levels of protection are rarely achieved and widespread use of polysaccharide vaccines has not prevented continuing epidemics in Africa [4], [19]. Meningococcal polysaccharide vaccines conjugated to immunogenic proteins (i.e. diphtheria toxoid, tetanus toxoid) are more immunogenic in infants than polysaccharide vaccines, and induce immunological memory and herd immunity [20], [21]. Meningococcal serogroup C conjugate vaccines are now available and have proved to be highly immunogenic, even in young infants. They reduce carriage and induce herd immunity [22], [23]. In the United Kingdom, serogroup C conjugate vaccines have proved to be highly effective in preventing invasive serogroup C meningococcal disease in all age groups but a booster dose is recommended in the second year of life to optimize the protection afforded by primary vaccination of infants [22]. Since 2005, a tetravalent ACWY conjugate vaccine has been available in the United States for use in subjects aged 11 years and older [24].

Two meningococcal A+C conjugate vaccines, employing diphtheria toxoid or a mutant of diphtheria toxoid (CRM197) as a carrier protein, have shown immunogenicity in infants during clinical studies in Africa [25], [26], but these vaccines were not taken forward into commercial development. The MenA component of one of them failed to demonstrate induction of immune memory [27]. In subjects who received a booster at 2 years of age, the immune response was long lasting (active up to 5 years of age) and superior to that obtained with polysaccharide vaccines [28].

The optimum schedule of administration of conjugate vaccines is not yet well defined. This was highlighted in a study that showed that 2 doses of conjugate Men A/C vaccine given at 2 and 6 months of age induced a lower MenC antibody response than a single dose given at 6 months of age [26]. The Meningitis Vaccine Project is developing a monovalent serogroup A meningococcal conjugate vaccine to be used in mass vaccination [29]. To offer African infants a broader protection against the most prevalent forms of meningococcal disease, GlaxoSmithKline (GSK) Biologicals has developed a combined *Haemophilus influenzae* type b, meningococcal serogroup A and C (Hib-MenAC) conjugate vaccine to be mixed extemporaneously with its diphtheria, tetanus, whole-cell pertussis, hepatitis B (DTPw-HBV) vaccine. Such a combination has already been shown to have a good reactogenicity and immunogenicity profile, including induction of immune memory, in the Philippines [30]. It can be given within the routine immunization calendar and thus has the potential to achieve high vaccine coverage.

The aim of the present study was to evaluate the immunogenicity, reactogenicity and safety of this combination vaccine when given to infants as a three-dose primary vaccination (following the EPI schedule) in an area within the African meningitis belt.

## Materials and Methods

The protocol for this trial and supporting CONSORT checklist are available as supporting information; see Checklist S1 and Protocol S1.

The primary objectives of the study (study ID:759346/009 & 104430; ISRCTN:35754083) were to demonstrate that the DTPw-HBV/Hib-MenAC candidate vaccine is non-inferior to the currently used DTPw-HBV/Hib vaccine with respect to the immunogenicity of the five antigens shared between the two vaccines, and to demonstrate the immunogenicity of the MenA and MenC conjugate antigen components of the new candidate vaccine.

Prior to the launch of the study, the protocol and study materials were approved by the Ghana Health Service Ethical Review Committee, the London School of Hygiene & Tropical Medicine Ethics Committee, Institutional Review Board of the Navrongo Health Research Centre and the World Health Organization Research Ethics Review Committee (ERC). The trial was conducted in accordance with ICH GCP guidelines. At the time of enrollment, the HIV prevalence amongst antenatal attendants was 2.8% [31].

### Study area

The study was carried out in the Kassena-Nankana District, Upper East Region, Ghana during the period between June 2004 and October 2005. The Kassena-Nankana District lies in a region of wooded Guinea savanna and has a seasonal climate with a long dry season and a short rainy season. It is situated within the African meningitis belt and experienced a large epidemic of serogroup A meningococcal disease in 1996/1997. The ecology of pharyngeal colonization with *N. meningitidis* has been studied in detail in the area since 1998 [32]. Serogroup A/C meningococcal polysaccharide vaccine has been used widely in the region since 1997. None of the study infants had received a meningococcal vaccine prior to entering the study but some of their mothers had been vaccinated (see below).

### Study participants

Male and female infants who had a birth weight ≥2 kg (when known) and who were 6 to 8 weeks of age at the time of first vaccination were enrolled in the study provided that they had none of the following exclusion criteria: (1) malnutrition, (2) a history of neurological disorder or immunodeficiency, (3) a family history of immunodeficiency, (4) a history of receiving immunosuppressants, (5) previous vaccines or treatments prohibited by the protocol, (6) exposure, by direct contact, to diphtheria (D), tetanus (T), pertussis (Pw), hepatitis B (HBV), *Haemophilus influenzae* type b (Hib), and/or meningococcal disease since birth. Witnessed, written, informed consent was obtained from the parents or guardians of each infant before enrolment.

### Study design

The study was controlled and double-blind. Infants were allocated to either DTPw-HBV/Hib-MenAC (study group) or DTPw-HBV/Hib (control group), following a randomization-blocking scheme (1:1 ratio, block 4, no covariates were used for blocking).

### Vaccination

The study vaccine (DTPw-HBV/Hib-MenAC) comprised one vial of Hib-MenAC, containing 5 μg of each polysaccharide conjugate, reconstituted extemporaneously with two vials of DTPw-HBV; only half of the mixture was administered as each vial contained two doses. The control vaccine was the commercially available DTPw-HBV/Hib vaccine *Tritanrix*™-HBV/*Hiberix*™ (GSK Biologicals). Three doses of study or control vaccine were injected intramuscularly, the first at 6 weeks (range 6–8 weeks) of age, the subsequent ones at 4 week intervals (range 28–42 days). When aged 12 months, each child received a challenge dose of 10 μg of meningococcal polysaccharides A and C, given as one fifth of a dose of GSK Biologicals' *Mencevax AC™* that contains 50 μg of each polysaccharide.

To comply with the Ghanaian routine immunization schedule [33], infants received Bacille Calmette-Guérin (BCG) and oral polio vaccine (OPV) at birth (or up to 2 weeks before the subject's first visit). OPV could be given concomitantly with the study vaccines or at any time during the study. Measles and yellow fever vaccines were administered to each study infant at 9 months of age. *Tritanrix*-HepB, *Hiberix* and *Mencevax* AC are trademarks of the GlaxoSmithKline group of companies.

### Reactogenicity and safety

Subjects were monitored for any immediate reactions to vaccination for 30 minutes after each injection. Field workers, blinded to study group, visited subjects at their home on the day of vaccine administration and on the 3 subsequent days to record on diary cards body temperature and any local or general adverse events. The occurrence of pain, swelling, fever, drowsiness, loss of appetite, or irritability was solicited from the parents or guardians. Any adverse events occurring within 30 days after administration of each dose of vaccine were also recorded on diary cards and reviewed by the investigators. Serious adverse events (SAEs), defined as any hospitalization, death or life-threatening medical condition, were recorded throughout the study and reviewed by the Ghanaian National Ethics Committee as well as by the Institutional Review Board of the Navrongo Health Research Centre. An Independent Data Monitoring Committee (IDMC) reviewed the safety and reactogenicity data after the first dose had been given to the first 60 infants; the IDMC also reviewed all SAEs on a quarterly basis.

### Immunogenicity

Four blood samples (3.5 ml) were collected from all subjects at the following times-immediately prior to administration of the first dose of study vaccine, one month after the third dose, immediately before the polysaccharide challenge and one month afterwards. Frozen samples were shipped to GSK Biologicals for serological assays. Serogroup A and serogroup C bactericidal antibody titres (SBA-MenA and SBA-MenC) were determined using a well standardized assay which utilizes baby rabbit serum as the source of complement (Pel-Freeze Incorporated, Rodgerson, AZ, USA) [34]. The strains used in the assays were F8238 for MenA and C11 for MenC. Titres of bactericidal antibody were expressed as the reciprocal of the dilution resulting in 50% killing after 60 minutes incubation. A titre of 1∶8 or greater was used to define seropositivity. A SBA titre of at least 1∶8 has been validated as protective against serogroup C invasive meningococcal disease [35]. For serogroup A meningococcal disease, no protective SBA titre has yet been validated but an anti-PSA IgG concentration of 2 μg/ml (measured by Radioactive Antigen Binding Assay: RABA) has been shown to be associated with protection [36]. In this study, IgG antibodies to meningococcal polysaccharides A and C were measured by an ELISA (cut-off for positivity = 0.30 μg/ml) [37]. Antibodies to diphtheria toxoid (D)(cut-off for positivity = 0.1U/ml), tetanus toxoid (T) (cut-off for positivity = 0.1IU/ml), inactivated *Bordetella pertussis* (whole cell) (BPT) (cut-off for positivity = 15 EL.U/ml), to hepatitis B surface antigen (HBV) (cut-off for positivity = 10 mIU/ml) and to Hib polyribose phosphate (PRP) (cut-off for positivity = 0.15 μg/ml) were quantified using standardized ELISAs at GSK Biologicals, Rixensart, Belgium.

Post-vaccination serum samples with ELISA anti-D antibody concentrations <0.1 IU/ml were re-tested using a VERO cell neutralization assay [38]. The cut-off for the VERO cell assay associated with protection is 0.016 IU/ml. Subjects with antibody response≥the assay cut-off were considered protected against Hib-disease, diphtheria, tetanus or hepatitis B.

### Statistical analyses

To cope with the multiplicity of study objectives, the pre-set criteria for meeting the study objectives were assessed sequentially. The sample size for the primary According-To-Protocol (ATP) cohort for immunogenicity comparisons was set at 280 enrolled subjects allowing for 60 drop-outs. A total of 220 evaluable subjects provided a study cumulative power to meet each criterion of co-primary endpoints individually (each individual power was at least 84%, except for anti-PRP for which it was 79.8%).

Data analysis was performed at GSK Biologicals using SAS software-Proc Plan (version 8.2) and StatXact (version 5.0). The primary immunogenicity analysis was performed on the initial ATP cohort, comprising all vaccinated subjects complying with the protocol and for whom assay results were available for antibodies against at least one study vaccine antigen component at one month post administration of vaccine dose 3. The analysis of persistence was performed on the ATP cohort for persistence, which comprised children vaccinated with the 3 primary vaccine doses and the polysaccharide challenge who had not received immunoglobulin or blood transfusion. The post-challenge ATP immunogenicity analysis was performed on children who complied with the protocol for the challenge part of the study and for whom assay results were available for antibodies against at least one study vaccine antigen one month post the challenge dose. Immune responses were expressed as geometric mean antibody concentrations (GMC) for antibodies detected by ELISA, or as geometric mean titres (GMT) for antibodies detected by serum bactericidal activity (SBA). In an exploratory way, the non-overlapping of the 95% CIs between the 2 groups was used as an indicator of a statistically significant difference. The primary objective was met if the differences between the groups were less than 10% (lower limit [LL] of the 95% confidence interval [CI] of difference between study population *minus* control more than −10%) in terms of seroprotection rate for D, T, HBV, and Hib antibodies, or if UL of the group ratio GMC was below 1.5 for pertussis antibodies, and if the lower limit of the 95% CI for SBA titres of at least 1:8 was >80% (for both serogroups A and C).

All 280 vaccinated subjects who received at least one primary vaccination dose of study vaccine (total vaccinated cohort) were included in the analysis of safety. The incidences of solicited symptoms, as well as of those graded 3 (i.e. preventing daily activity, or swelling >30mm, or rectal fever >40°C), within the 4-day follow-up period were tabulated over the whole primary vaccination course (i.e. per dose), with exact 95% CIs. The incidence of unsolicited symptoms during the 31-day follow-up period after administration of each dose of vaccine was calculated using the standard terminology of the Medical Dictionary for Regulatory Activities (MedDRA®) primary system organ class and primary term. Reactogenicity and safety after the challenge dose of polysaccharide were assessed similarly. Only unsolicited symptoms and Serious Adverse Events are discussed in this paper. Serious adverse events were described according to the following study period: from enrolment up to one month after the third dose of the primary vaccination series, from one month after the third dose of vaccine up to the time of the challenge dose and for the period of one month after the challenge dose had been given.

## Results

### Study population

**Figure 1:**
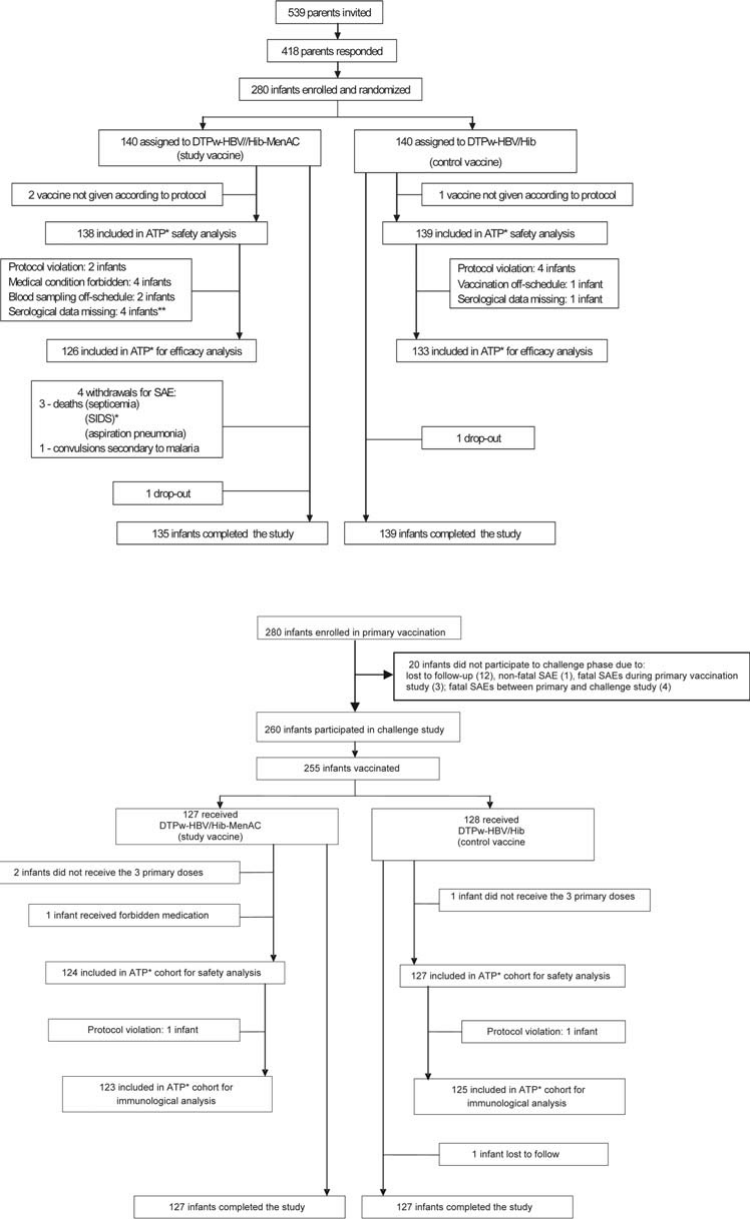
Study profile. Footnote: ATP: according to protocol; SIDS: sudden infant death syndrome; ** number of subjects with the elimination code assigned excluding subjects who have been assigned a lower elimination code number.

Two hundred and eighty of the 418 infants whose parents responded to an invitation to join the study were identified as eligible and were enrolled (Figure 1): 6 did not complete the study (4 in the study group due to SAEs and 1 subject in each study group who was lost to follow-up). Thus, 97.8% completed the primary vaccination phase of the study. At enrolment, the demographic profiles of infants in each group were comparable with no significant differences between groups (Table 1). Two hundred and fifty-nine children were included in the analysis of immunogenicity one month after the third dose. Of the 260 children who were part of the polysaccharide challenge phase of the study, 251 were included in the analysis of persistence of antibodies following vaccination and 248 in the post challenge immunogenicity analysis. Reasons for non inclusion in each phase of the study are specified in Figure 1.

**Table 1:** Demographic profile of infants in the study and control groups.

	DTPw-HBV/Hib-MenAC (study vaccine) (N = 140)	DTPw-HBV/Hib (control) (N = 140)
Mean age (± SD) at first vaccination (in weeks)	6.3 (± 0.47)	6.4 (± 0.56)
Females/males	74/66	72/68
Mean height (± SD) (in cm)	54.5 (± 2.50)	55.0 (± 2.49)
Mean weight (± SD) (in kg)	4.4 (± 0.59)	4.5 (± 0.65)

Approximately one quarter of the infants' mothers had received a meningococcal vaccine during the year preceding the infant's vaccination (38/140 vs. 37/140 for the study vaccine and the control vaccine groups respectively).

### Immunogenicity

**Figure 2:**
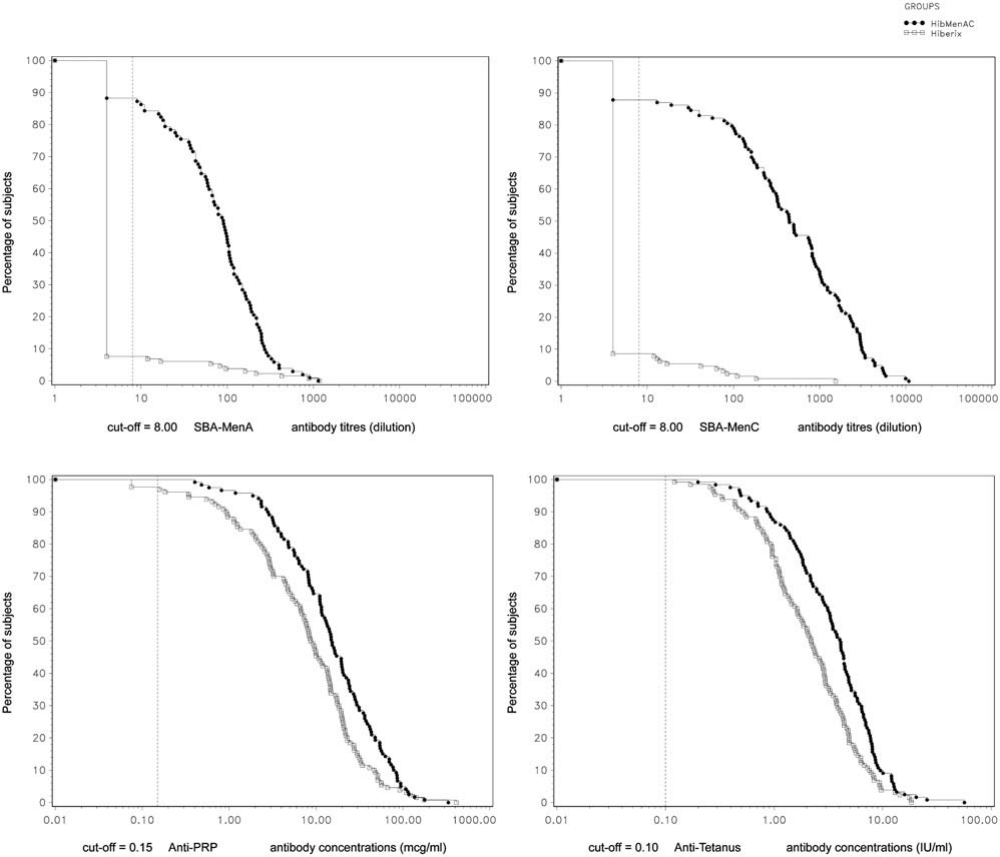
Reverse cumulative curves for antibody concentration/titre, 1 month after the third dose of vaccine (Primary ATP cohort for immunogenicity). Footnote: HibMenAC = DTPw-HBV/Hib-MenAC; Control = DTPw-HBV/Hib.

One month after the third dose of vaccine had been given, the lower limits of 95% CI of differences between groups in seroprotection rates (for diphtheria, tetanus, hepatitis B, Hib) or the upper limit of the 95% CI of the group ratio of GMC (for pertussis) were all within the pre-determined criteria for non-inferiority (Table 2). The new heptavalent combination was therefore non-inferior to the currently available pentavalent vaccine for the five antigens common to the two vaccines. The immune responses to tetanus and PRP antigens (GMCs) were higher in infants who received the study vaccine than in those who received the control vaccine (Figure 2).

**Table 2:** Primary objectives: inferential sequential analysis 1 month after the third vaccine dose (primary ATP cohort for immunogenicity)

Co-primary endpoints	Pre-set, non-inferiority criteria	Values (95%CI)
SBA-MenA ≥1:8	LL# seropositivity rate **>80%**	88.2 (**80.4**, 93.8)
SBA-MenC ≥1:8	LL# seropositivity rate **>80%**	87.8 (**80.7**, 93.0)
Anti-PRP ≥1 μg/ml	LL# group difference **>−10%**	8.18 (**1.76**, 15.26)
Anti-tetanus ≥0.1 IU/ml	LL# group difference **>−10%**	0.00 (**−3.08**, 2.87)
Anti-diphtheria ≥0.1 (ELISA) or 0.016 (Verocell) IU/ml	LL# group difference **>−10%**	−0.81 (**−4.46**, 2.05)
Anti-BPT concentration	LL# group ratio of adjusted GMCs† **>0.67**	0.86 (**0.73**, 1.00)
Anti-HBs ≥10 mIU/ml	LL# group difference **>−10%**	0.63 (**−7.94**, 9.05)

Note : antigens sequenced by priority

PRP = polyribosylribitol phosphate; BPT = Bortedella pertussis ; HBs = Hepatitis B surface antigen

# Lower limit of the 95% CI

† Adjusted GMC = GMC adjusted for baseline concentration using ANCOVA model

Seropositivity rate = % subjects above cut-off

Group difference = % subjects in vaccine group above cut-off-% subjects in control group above cut-off

Group ratio = GMC vaccine group/GMC control group

The lower limit of the 95%CI of the percentages of infants with SBA-MenA and SBA-MenC titres of at least 1:8 was well above the pre-set criterion of 80%, demonstrating the immunogenicity of the MenA and MenC conjugate antigens in the new candidate vaccine (Table 2). The proportion of infants with SBA-MenA titres ≥ 1:8 was significantly higher in the study vaccine group than in the control group : 88.2% (95%CI [80.4–93.8]) vs 7.6% (95%CI [3.7–13.6]) (Table 3). Similarly, the proportion of infants having SBA-MenC titres ≥1∶8 was significantly higher in the study vaccine group than in the control group: 87.8% (95%CI [80.7–93.0]) vs 8.6% (95%CI [4.4–14.9]). All subjects in the study group had anti-PSA and anti-PSC concentrations ≥0.3 μg/ml (Table 3) one month after the third dose. 94.9% and 96.7% of children in the study vaccine group had anti-PSA and anti-PSC concentrations respectively of at least 2.0 μg/ml (data not shown). Analysis of immunogenicity according to pre-vaccination status showed that one month after administration of the third dose of meningococcal conjugate vaccine SBA MenC and SBA MenA GMTs were lower in infants with pre-existing bactericidal antibodies (S+) compared to those with no pre-existing bactericidal antibodies S- (S+ 150.7; 95%CI [89.0–255.2], S- 704.5; 95% CI [412.7–1202.4] and S+ 54.9; 95% CI [31.7–95.2], S- 80.2; 95% CI [56.9–113.1]) for serogroup C and serogroup A antibodies respectively. Although 95% confidence limits overlapped for both assays, anti-PSC GMCs tended to be higher in S- subjects (23.54 μg/ml; 95%CI [15.94–34.76]) than in S+ subjects (S+) (13.72 μg/ml; 95%CI [11.55–16.30]), whereas anti-PSA GMCs were within the same range: 8.19 μg/ml; 95%CI [5.67–11.83] in S- and 6.27 μg/ml; 95%CI [5.35–7.35] in the S+ subjects.

**Table 3:** Seropositivity/seroprotection rates and GMTs/GMCs before and 1 month after primary vaccination (ATP cohort for the primary immunogenicity study)

		Study vaccine*			Control vaccine**		
Antibody	Timepoint to vaccination	N	% subjects with titres/concentrations ≥ cut-off (95% CI)	GMT or GMC (95% CI)	N	% subjects with titres/concentrations ≥ cut-off (95% CI)	GMT or GMC (95% CI)
SBA-MenA	Pre	112	27.7 (19.6,36.9)	8.4 (6.5, 10.8)	122	24.6 (17.2, 33.2)	7.4 (6.0, 9.3)
	Post-dose III	102	88.2 (80.4, 93.8)	65.0 (49.4, 85.6)	131	7.6 (3.7, 13.6)	5.2 (4.4, 6.2)
SBA-MenC	Pre	118	47.5 (38.2, 56.9)	17.2 (12.5, 23.6)	118	44.1 (34.9, 53.5)	14.3 (10.7, 19.1)
	Post-dose III	123	87.8 (80.7, 93.0)	327.5 (223.9, 479.0)	128	8.6 (4.4, 14.9)	5.0 (4.3, 5.8)
Anti-PSA	Pre	108	75.0 (65.7, 82.8)	0.9 (0.7, 1.2)	115	75.7 (66.8, 83.2)	0.9 (0.7, 1.15)
	Post-dose III	118	100.0 (96.9, 100.0)	6.4 (5.5, 7.4)	127	24.4 (17.2, 32.8)	0.2 (0.2, 0.3)
Anti-PSC	Pre	108	77.8 (68.8, 85.2)	1.0 (0.8, 1.3)	118	78.0 (69.4, 85.1)	1.0 (0.8, 1.2)
	Post-dose III	122	100.0 (97.0, 100.0)	15.5 (13.4, 17.9)	127	34.6 (26.4, 43.6)	0.3 (0.2, 0.3)
Anti-PRP	Post-dose III	119	100.0(1) (96.9, 100)	14.9 (11.8, 18.9)	130	97.7(1) (93.4, 99.5)	7.4 (5.6, 9.8)
			96.6(2) (91.6, 99.1)			88.5(2) (81.7, 93.4)	
Anti-tetanus	Pre	102	93.1 (86.4, 97.2)	1.0 (0.7, 1.2)	110	97.3 (92.2, 99.4)	0.9 (0.8, 1.2)
	Post-dose III	121	100.0 (97.0, 100.0)	3.4 (2.9, 4.1)	130	100.0 (97.2, 100.0)	2.1 (1.7, 2.5)
Anti-diphtheria	Post-dose III	123	99.2† (95.6, 100.0)†	1.2 (1.0, 1.4)††	131	100.0† (97.2, 100.0)†	1.3 (1.1, 1.5)††
Anti-BPT	Post-dose III	125	98.4 (94.3, 99.8)	132.9 (117.4, 150.5)	132	100.0 (97.2, 100.0)	160.8 (147.4, 175.5)
Anti-HBs	Post-dose III	117	88.0 (80.7, 93.3)	131.4 (94.4, 183.0)	127	87.4 (80.3, 92.6)	218.1 (159.8, 297.5)

* DTPw-HBV/Hib-MenAC

** DTPw-HBV/Hib

PSC/A = serogroup S/A polysaccharide ; PRP = polyribosylribitol phosphate; BPT = Bortedella pertussis ; HBs = Hepatitis B surface antigen

Dilution titre (SBA), μg/ml (anti PSA/C, anti PRP), IU/ml (anti–tetanus and anti-diphtheria), EL U/ml (anti-BP), mIU/ml (anti-HBs).

(1) : anti PRP ≥ 0.15 μg/ml ;

(2) : anti PRP ≥ 1.0 μg/ml

† ≥0.1IU/ml by ELISA or ≥ 0.016IU/ml by VERO cell neutralisation test when concentration by ELISA<0.1IU/ml (seroprotection);

†† : by ELISA

N = number of subjects with available results; Pre = pre-primary vaccination; Post-dose = 1 month post-dose 3

95% CI = 95% confidence interval

GMT/GMC = geometric mean antibody titre/concentration calculated on all subjects

**Figure 3:**
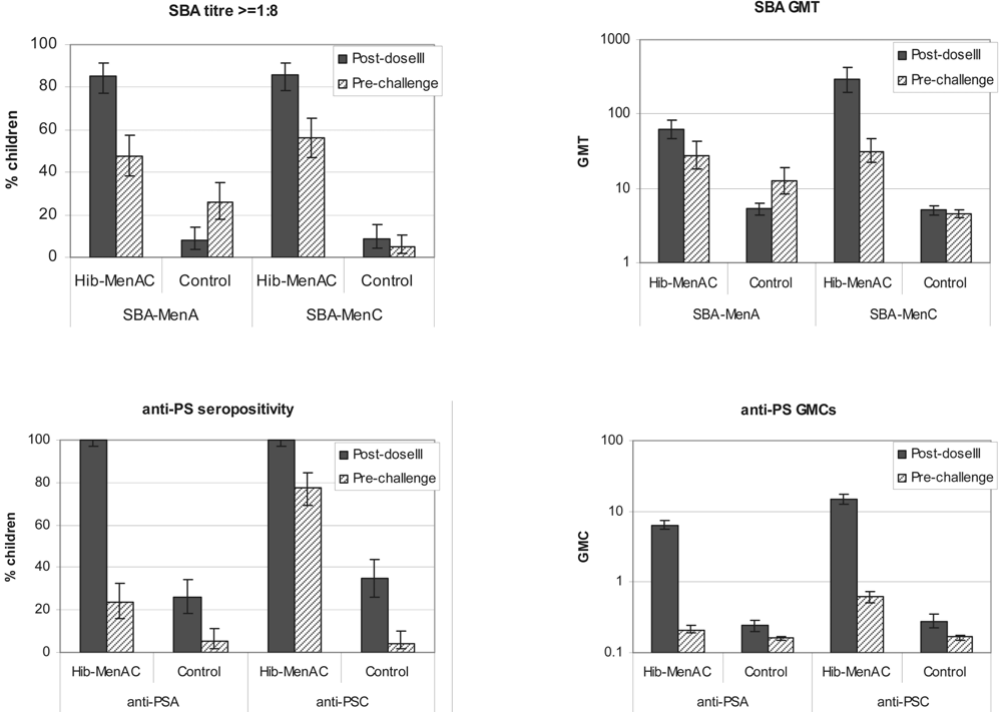
Persistence of meningococcal antibody responses. Seropositivity rates and GMTs/GMCs after primary vaccination (Post-dose III) and before the polysaccharide challenge (Pre-challenge) (ATP cohort for persistence). Footnote: Error bars represent 95%CI.

**Figure 4:**
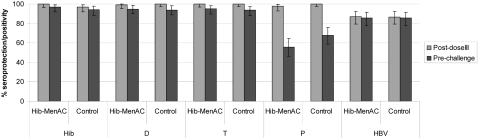
Seropositivity/seroprotection rates for other antigens (D*, T, P, HBV) after primary vaccination (Post-dose III) and before the polysaccharide challenge (Pre-challenge) (ATP cohort for persistence for diphtheria, ATP cohort for immunogenicity for post-dose III). Footnote: Error bars represent 95%CI.

Prior to challenge with MenA and MenC polysaccharides, 47.7% and 56.4% of subjects in the study group had SBA-MenA and SBA-MenC antibody titers ≥1∶8 respectively compared with 25.7% and 5.1% of children in the control group (Figure 3). Persistence of antibodies to the other antigens present in each vaccine was within the same range as in the control group (Figure 4). One month after polysaccharide challenge, SBA-MenA and SBA-MenC GMTs in children in the study vaccine group increased 14.0 and 18.8-fold respectively compared with 3.8 and 1.9-fold increases in children in the control group. The percentages of subjects with SBA titres ≥1∶8 following challenge were significantly higher in children in the study group (93.5% for MenA and 88.5% for MenC) than in those in the control group (51.4% for MenA and 18.2% for MenC)(Table 4).

**Table 4:** Seropositivity rates and GMTs/GMCs before (pre-challenge) and 1 month after (post-challenge) a polysaccharide challenge dose had been given (booster ATP cohort for immunogenicity)

		Study vaccine*			Control vaccine**		
Antibody	Timepoint to vaccination	N	Seropositivity (%) (95% CI)	GMT or GMC 95% CI)	N	Seropositivity(%) ≥ 1: 8 (95% CI)	GMT or GMC (95% CI)
SBA-MenA	Pre-challenge	109	47.7 (38.1, 57.5)	27.8 (18.2, 42.3)	103	25.2 (17.2, 34.8)	12.2 (8.3, 18.1)
	Post-challenge	107	93.5 (87.0, 97.3)	390.1 (280.2, 543.0)	109	51.4 (41.6, 61.1)	46.4 (28.8, 74.7)
SBA-MenC	Pre-challenge	117	56.4 (46.9, 65.6)	31.6 (21.9,45.8)	116	5.2 (1.9, 10.9)	4.6 (4.0, 5.1)
	Post-challenge	122	88.5 (81.5, 93.6)	593.5 (398.1, 884.7)	121	18.2 (11.8, 26.2)	8.7 (6.2, 12.3)
Anti-PSA	Pre-challenge	115	23.5 (16.1, 32.3)	0.21 (0.19, 0.24)	115	5.2 (1.9, 11.0)	0.16 (0.15, 0.17)
	Post-challenge	120	90.8 (84.2, 95.3)	4.72 (3.41, 6.52)	117	47.0 (37.7, 56.5)	0.38 (0.30, 0;48)
Anti-PSC	Pre-challenge	116	78.4 (69.9, 85.5)	0.62 (0.52, 0.73)	113	4.4 (1.5, 10.0)	0.17, (0.15, 0.18)
	Post-challenge	119	98.3 (94.1, 99.8)	4.29 (3.57, 5.14)	121	100 (97.0, 100)	3.58 (3.03, 4.24)

* DTPw-HBV/Hib-MenAC

** DTPw-HBV/Hib

PSA/C = serogroup A/C polysaccharide ; % subjects = percentage of subjects with seroprotective/seropositive titre/concentration of at least 1:8 (SBA-MenA/C), 0.3 μg/ml (anti PSA/C), 95% CI = 95% confidence interval

GMT/GMC = geometric mean antibody titre/concentration calculated on all subjects.

### Reactogenicity and safety

**Figure 5A:**
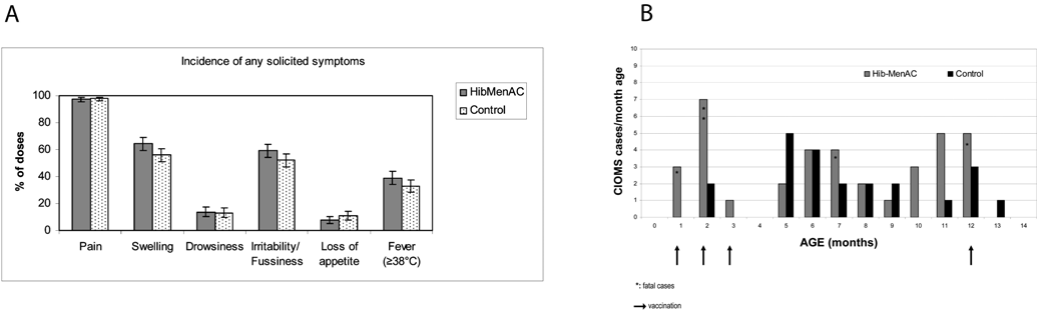
Incidence of adverse events. A: Solicited local and general symptoms within the 4-day period after vaccination. Footnote: Error bars represent 95%CI. B: SAEs distribution over time in the total vaccinated cohort. HibMenAC: DTPw-HBV/Hib-MenAC (study vaccine); Control : DTPw-HBV/Hib (control vaccine)

Incidences of reported symptoms following primary vaccination were similar in both groups, (Figure 5A) with >99% of doses apparently causing symptoms. Most doses caused pain at the injection site (>97%) in both groups, but none were graded 3. The study vaccine tended to induce more episodes of swelling >30 mm than the control vaccine (23.7% of doses vs. 14.1%, no overlapping of the 95%CI) but in all such cases swelling resolved within 9 days. The most prevalent general symptoms solicited were irritability and fever (Figure 5A). Fever >39°C occurred following 2.9% (95%CI [1.5–5.1]) of doses of the study vaccine and following 1.7% (95%CI[0.7–3.4]) of doses of the control vaccine. Only one child had fever >40°C (after the third dose of the DTPw-HBV/Hib-MenAC vaccine), and this lasted for only one day.

Unsolicited adverse events reported following primary vaccination showed a similar pattern in each group: 99 malaria episodes in 79 subjects were reported in the study vaccine group (within 30 days following vaccination) compared to 110 malaria episodes in 84 subjects in the control group, but most cases were not confirmed by microscopy. Other frequent, unsolicited adverse events were respiratory infections (82 vs. 82), injection site erythema (65 vs. 61), and diarrhoea (46 vs. 33) in the study and control vaccine groups respectively.

The percentage of subjects with unsolicited adverse events reported within 31 days following the administration of the challenge dose of polysaccharide vaccine was similar in both groups (55.1% in the vaccine group and 55.5% in the control group). Most adverse events were infectious diseases such as malaria, upper respiratory tract infections and dysentery, but none of these unsolicited events was considered causally related to vaccination. Grade 3 unsolicited symptoms were reported only infrequently in both groups (only three subjects): all 3 subjects had malaria (1 in the study vaccine group; 2 in the control group), one subject also had sepsis (study vaccine group) and another anaemia (control group).

A total of fifty-nine SAEs were reported throughout the study-37 in subjects who had received the study vaccine and 22 in subjects who had received the control vaccine. None of the SAEs was considered by the investigators or the IDMC to be related to vaccination. Thirteen of the SAEs were reported during the primary vaccination phase of the study (Figure 5B), 11 in subjects in the study vaccine group and 2 in the control group. Three deaths were reported (dysentery+impetigo+septicaemia, aspiration pneumonia, and sudden infant death syndrome) among subjects in the study vaccine group. The diagnoses of sudden infant death syndrome and dysentery+septicemia were made by verbal autopsy, while the third subject died during hospitalization. The 10 non-fatal cases with SAEs were all hospitalized and recovered: 8 were laboratory-confirmed cases of malaria (with or without complications including a respiratory tract infection, typhoid fever or enteritis). One child had broncho-pneumonia and one gastroenteritis. During the period from the end of the primary vaccination phase up to administration of the challenge dose of polysaccharide vaccine a further 38 SAEs were recorded. Twenty-two SAEs (including 1 death due to malaria, anaemia, bronchopneumonia and dehydration) were reported in the study vaccine group and 16 (including 3 deaths-2 due to malaria and 1 due to gastro-enteritis and dehydration) were reported in the control group. During the month after challenge a further 8 SAEs (all non-fatal) were reported; these were equally distributed between the study and the control groups. All except one non-fatal SAE resolved. The one exception occurred in a child with a history of head injury who had tonic convulsion secondary to malaria 13 days after receiving the second vaccine dose as well as a respiratory tract infection. The child recovered but developed epilepsy and discontinued the study.

## Discussion

This study has shown that both the MenA and MenC conjugate antigens present in the novel DTPw-HBV/Hib-MenAC combination vaccine candidate are immunogenic and induce bactericidal antibody titres ≥1∶8 in close to 90% of infants compared to a prevalence of less than 10% prevalence in unvaccinated control infants. High levels of immunity to serogroup A exist in populations living in the “meningitis belt” [39], [40]. Carriage of meningococci of serogroup A and C was reported to be low in Burkina Faso, [40], [41] although a study reported 17% carriers in Nigeria [42]. The high level of bactericidal antibodies in our study may be due to either cross-reacting antibodies or vaccination since a large segment of the population is routinely vaccinated with meningococcal polysaccharide vaccine. Thus, the presence of bactericidal antibodies (SBA titre ≥1∶8) in a substantial proportion of infants before vaccination (44–48% of infants had SBA-MenC, 25–28% had SBA-MenA titres at this level) is not surprising, probably reflecting the transfer of maternally derived antibody. The proportion of infants in the control vaccine group with titres above this level fell during the following months reflecting a natural decline in maternally acquired antibody. In contrast, bactericidal antibody titres persisted in infants who had received the study vaccine and at 12 months of age, approximately half of them still had SBA titres ≥1∶8 for each serogroup. The higher prevalence of anti-PSA (78%) and anti-PSC (75%) maternal antibodies compared to bactericidal antibodies prior to vaccination suggest that not all meningococcal antibodies transferred by the mother to her infant are bactericidal. When mothers were asked whether they had been vaccinated against meningitis in the year preceding the start of the study, approximately one quarter of them remembered being vaccinated; the protective immune response to meningococcal polysaccharide vaccines in adults is known to last for at least 3 years [43], [44]. Vaccination against meningitis is undertaken repeatedly in Northern Ghana and this could explain the high background prevalence of anti-PSA and anti-PSC seropositivity in our study.

MenA and Men C bactericidal antibody titres were lower in Ghanaian infants than those obtained in a similar study undertaken in the Philippines where 97.7 and 99% of vaccinated infants had SBA titres ≥1∶8 for Men A and Men C respectively; GMTs were 316.7 (95%CI [251.4–398.9]) and 3132.6 (95%CI [2496.9–3930.1]) for serogroups A and C respectively [30]. The present study was conducted in a tropical country but the cold-chain was closely monitored and there were no major deviations of the temperature at which the vaccines were kept (2–8°C). The immune response to the other antigens in the combined vaccine was as expected, so it is highly improbable that an undetected break in the cold-chain occurred. Another possibility that was considered was that the immune response of Ghanaian infants to the meningococcal conjugates might have been suppressed by high antibody concentrations in their mothers induced by natural exposure to meningococci or by vaccination. Therefore, the influence of infants' pre-vaccination antibody status (SBA-MenA, SBA-MenC, anti-PSA, anti-PSC) on the immune response to the new vaccine was studied. The response to the MenC component of the study vaccine was lower in infants seropositive for SBA-MenC or anti-PSC prior to vaccination, as has been found in the United Kingdom with the meningococcal serogroup C conjugate vaccine [45]. In contrast to MenC, pre-existing anti-MenA antibodies did not influence the immune response to the MenA component of the vaccine. Another possible reason for the lower immunogenicity seen in the present trial compared with the one undertaken the Philippines might be the very high level of pre-existing anti-TT antibodies found in the prevaccination samples of Ghanaian infants in the study vaccine and control groups (93% and 97% respectively) resulting from the fact that most women had been vaccinated against tetanus during pregnancy following the recommendation of the WHO [46]. However, a negative impact of pre-existing anti-TT antibodies on the MenAC response (the MenAC conjugate uses TT as a carrier) is unlikely as the anti-PRP response was higher in infants who received the study vaccine than in those who received the control vaccine, even though the Hib conjugate also uses a tetanus carrier. This effect was seen even though the quantity of PRP used in the study vaccine was 4 times less than in the control, probably as a result of a TT carrier effect as reported previously [21], [47]. Other possible explanations for the lower immunogenicity of the MenA and MenC components of the candidate vaccine in Ghanaian infants are malnutrition and malaria. Cases of malnutrition were excluded at study entry and no serious episodes of malnutrition were reported during the primary vaccination phase of the study. Malaria can suppress the response to many vaccines including Hib conjugate vaccines and meningococcal polysaccharide vaccines [48], [49] but malaria is relatively uncommon in the first six months of life, even in areas of high transmission. Malaria was reported in about half of the study infants during the primary vaccination phase of this study but, in most cases, diagnosis was not confirmed by microscopy. Further research may be needed to determine if malaria influences the immune response to the meningococcal conjugate antigens.

Non-inferiority of the DTPw-HBV/Hib-MenAC vaccine compared to the control vaccine was demonstrated for the five antigens common to each vaccine. Although seroprotection for hepatitis B was in the lower range of that found in published studies, it is comparable to that induced by the control vaccine and remains at an acceptable level given that no hepatitis B vaccine was administered at birth and that similar levels of protection have been seen previously following immunization with a hepatitis B monovalent vaccine in other African countries using the same immunization schedule [50].

The reactogenicity of DTPw-HBV/Hib-MenAC was similar to that of the control vaccine. The trend to more marked swelling at the injection site was without consequence and did not lead to any drop-outs. Three deaths reported in the study group within one month of primary vaccination were unrelated to the vaccine. These deaths, which occurred during the rainy season, when mortality is highest, are fewer than expected in a cohort of 280 children followed to the age of 7 months in a community with an infant mortality rate of 95.5/1000 [51]. One infant abandoned the study after a malaria episode followed by epilepsy; there is now good evidence that malaria increases the risk of epilepsy [52]. Although most SAEs during the primary vaccination phase of the trial were in the study vaccine group (11 vs. 2), the pattern of SAEs was that of common infections in this community, such as malaria and acute respiratory infections, and none of the events were considered to be vaccine related. The occurrence of a higher incidence of SAEs in the study than in the control group was probably a result of chance as evidenced by a more extended safety follow-up that was done up to the end of the challenge phase which did not reveal a difference in the overall incidence of SAEs between the groups. Furthermore, in a large safety study including 1780 infants vaccinated with either the DTPw-HBV/Hib-MenAC or the DTPw-HBV/Hib vaccines in Asia, the incidence of SAEs was similar in both groups [51]. Nevertheless, the safety of the new vaccine will require further evaluation in Africa, especially as a similar increase in SAEs was seen in a previous study that evaluated another meningococcal conjugate vaccine in Africa, although this was also considered to be most likely to be a result of chance [27].

Because the age distribution of cases of meningococcal disease in Africa covers a broader range than Hib or pneumococcal infections in children, introduction of the new vaccine into the EPI program would, alone, have only a limited impact on the overall burden of meningococcal disease until vaccination had been continued for many years. Recent experience in the UK with a MenC conjugate suggests that immunization in infancy gives only short-term direct protection and that a booster dose is required to provide sustained immunity [22]. Additional complementary vaccination strategies are needed in addition to infant immunization if a maximum effect is to be achieved. Vaccination of all children and young adults with a monovalent group A conjugate, or even better with a tetravalent ACWY conjugate, is a possible way of achieving this using infant immunization to maintain the pool of protected individuals.

In conclusion, the DTPw-HBV/Hib-MenAC vaccine is non-inferior to the control vaccine in inducing antibody titres that are associated with protection against 5 important childhood diseases in a single series of injections given during the routine vaccination EPI schedule. The vaccine induces bactericidal antibody titres which should provide protection against infection with group A and group C meningococci in infants in the “meningitis belt” during the first year of life. However, induction of more sustained protection will require booster immunization given routinely or perhaps through mass vaccination campaigns. Introduction of this combined conjugate vaccine into the routine immunization programme of countries within the meningitis belt could be an important option towards lowering the burden due to recurrent *N. meningitidis* epidemics in Africa.

## Supporting Information

Protocol S1 Trial Protocol
(0.80 MB PDF)

Checklist S1 CONSORT Checklist
(0.05 MB DOC)
