# Supplementary material for: A Phase II, Randomized Study on an Investigational DTPw-HBV/Hib-MenAC Conjugate Vaccine Administered to Infants in Northern Ghana
Source: PLoS One. 2008 May 14;3(5):e2159. doi: 10.1371/journal.pone.0002159 (PMC2374896; doi:10.1371/journal.pone.0002159)
Supplement: Protocol S1 — Trial Protocol (0.80 MB PDF) [file pone.0002159.s001.pdf]

Protocol
Amendment 7

GlaxoSmithKline Biologicals
Rue de l'Institut 89
1330 Rixensart, Belgium
Confidential & Proprietary Information

**Study Vaccine**	GlaxoSmithKline (GSK) Biologicals' *Haemophilus influenzae* type b-meningococcal AC-tetanus toxoid (Hib-MenAC) vaccine (as a mixed combination with Tritanrix™-HepB).
**eTrack study number** formerly Clinical Programs Monitoring System (CPMS) number 759346/009	759346/009 (DTPwHB/HIBMenACTT009) 104430 (DTPwHB/HIBMenACTT023 BST:009)
**Date of Approval**	Final Version 14 January 2003<br>Amendment 1: 05 June 2003<br>Amendment 2: 02 October 2003<br>Amendment 3: 05 January 2004<br>Amendment 4: 27 January 2004<br>Amendment 5: 2 April 2004<br>Modification 1: 7 July 2004<br>Amendment 6: 14 January 2005<br>**Amendment 7: 12 April 2006**
**Title**	A phase II, double blind, randomized, controlled study to evaluate the immunogenicity, reactogenicity and safety of GlaxoSmithKline (GSK) Biologicals' Hib-MenAC vaccine, extemporaneously mixed with GSK Biologicals' Tritanrix™-HepB, as compared to GSK Biologicals' Hiberix™ vaccine, extemporaneously mixed with GSK Biologicals' Tritanrix™-HepB, when administered intramuscularly in infants at 6, 10 and 14 weeks of age.

GlaxoSmithKline Biologicals

**Coordinating Authors:**	Uma Swaminathan ***and Chitra Nair***, Scientific Writer***s***
**Contributing Authors:**	Dr. Lode Schuerman, Director, Paediatric Vaccine Development<br>Dr. Dominique Boutriau, ***Director, Clinical Research & Development, Meningitis vaccines***<br>Cornelia Bevilacqua, Central Study Coordinator<br>Toufik Zahaf, Thierry Van-Effelterre ***and Christelle Durand***, Statisticians<br>**(Amendment 7: 12 April 2006)**

This protocol was developed based on Version 12 of GlaxoSmithKline Biologicals' Protocol Document Standard.
CARS Id : CLIN_200604_519/ Version : 1.2,Admin. QC/ Modify Date : 27/04/2006

GlaxoSmithKline Biologicals
Rue de l'Institut 89
1330 Rixensart, Belgium
Confidential & Proprietary Information

**Clinical Development Manager**	Dr. Dominique Boutriau, ***Director, Clinical Research & Development, Meningitis vaccines***<br>GlaxoSmithKline Biologicals,<br>89, Rue de l'Institut, 1330 Rixensart, Belgium<br>Tel: + 32 2 656.91.20<br>Fax: + 32 2 656. ***8044***<br>**(Amendment 7: 12 April 2006)**

Navrongo Health Research Center (NHRC), Navrongo, Ghana

**Contributing Author:**	Dr. Abraham Victor Obeng Hodgson

World Health Organization (WHO)

**Collaborating person:**	Dr. Marie-Paule Kieny

London School of Hygiene and Tropical Medicine

**Collaborating persons:**	Prof. Brian Greenwood<br>Dr. Daniel Chandramohan

For the ancillary study

Swiss Tropical Institute (STI), Basel, Switzerland

**Contributing Author:**	Dr. Gerd Pluschke, Molecular Immunology
**Principal Investigator:**	Dr. Abraham Victor Obeng Hodgson
**Co-investigators:**	Professor Fred Binka, University of Ghana, Accra, Ghana<br>Doctor Abudulai Adams Forgor, Navrongo Health Research centre, Navrongo, Ghana
**Co-investigator (for ancillary study only):**	Dr. Gerd Pluschke

This protocol was developed based on Version 12 of GlaxoSmithKline Biologicals' Protocol Document Standard.
CARS Id : CLIN_200604_519/ Version : 1.2,Admin. QC/ Modify Date : 27/04/2006

GlaxoSmithKline Biologicals
Rue de l'Institut 89
1330 Rixensart, Belgium
Confidential & Proprietary Information

Medical Monitor

GSK Biologicals:
Dr. Dominique Boutriau,
Director, Clinical Research & Development, Meningitis vaccines,
GlaxoSmithKline Biologicals,
89, Rue de l'Institut, 1330 Rixensart,
Belgium
Tel: + 32 2 656.91.20
Fax: + 32 2 656. ***8044***
e-mail: dominique.boutriau@gskbio.com

(Amendment 7: 12 April 2006)

Study Monitors

Cornelia Bevilacqua
'La Fédarié Haute'
81440 Vénès
France
Tel: + 33 563 75 92 66
Fax: + 33 563 75 92 66
Mobile phone: + 33 685 25 24 69
e-mail: corneliabevilacqua@usa.net

Valérie Marichal,
Central Study Coordinator,
GlaxoSmithKline Biologicals,
89, Rue de l'Institut, 1330 Rixensart,
Belgium
Tel: + 32 2 656.9812
Fax: + 32 2 656.8133
e-mail: valerie.marichal@gskbio.com

Local Safety Monitor

Dr. John Williams
Navrongo Health Research Center,
Ministry of Health, PO Box 114, Navrongo,
Ghana
Tel: + 233 742 22310/ 22380
Fax: + 233 742 22320
e-mail: jwilliams@navrongo.mimcom.net

Local Safety Monitor Back-up

Dr. Frank Baiden
Navrongo Health Research Center,
Ministry of Health, PO Box 114,
Navrongo, Ghana
Tel: + 233 742 22310/ 22380
Fax: + 233 742 22320
e-mail: fbaiden@navrongo.mimcom.net

GlaxoSmithKline Biologicals
Rue de l'Institut 89
1330 Rixensart, Belgium
Confidential & Proprietary Information

WHO Monitor

Dr.Lawrence Kweku Yamuah
Post-Doctoral Researcher / Data Manager
Armauer Hansen Research Institute (AHRI)
P.O. Box 1005
Addis Ababa
Ethiopia
Tel: + 251 1 71 02 88 (work)
Tel: + 251 1 71 17 04 (home)
Fax: + 251 1 71 13 90
Email:
yamuahlawrence_kweku@hotmail.com

WHO Study Contact for Reporting SAEs
Dr. Zarifah Reed
WHO/IVR/POP
WHO/IVB/IVR/POP
20 Avenue Appia
CH-1211 GENEVE 27
Switzerland
Tel. +41 22 791 4760
Fax. +41 22 791 4860
Email: reedz@who.int

Sponsor

GlaxoSmithKline Biologicals
Rue de l'Institut 89
1330 Rixensart, Belgium

Sponsor Information

Study Monitors

Cornelia Bevilacqua
'La Fédarié Haute'
81440 Vénès
France
Tel: + 33 563 75 92 66
Fax: + 33 563 75 92 66
Mobile phone: + 33 685 25 24 69
e-mail: corneliabevilacqua@usa.net

Valérie Marichal,
Central Study Coordinator,
GlaxoSmithKline Biologicals,
89, Rue de l'Institut, 1330 Rixensart,
Belgium
Tel: + 32 2 656.9812
Fax: + 32 2 656.8133
e-mail: valerie.marichal@gskbio.com

Study Monitor WHO

Dr.Lawrence Kweku Yamuah
Post-Doctoral Researcher / Data Manager
Armauer Hansen Research Institute (AHRI)
P.O. Box 1005
Addis Ababa
Ethiopia
Tel: + 251 1 71 02 88 (work)
Tel: + 251 1 71 17 04 (home)
Fax: + 251 1 71 13 90
Email: yamuahlawrence_kweku@hotmail.com

Study Contact for Reporting of a Serious Adverse Event at GSK Biologicals

Dr. Dominique Boutriau,
Director, Clinical Research & Development, Meningitis vaccines
GlaxoSmithKline Biologicals,
Rue de l'Institut 89, 1330 Rixensart, Belgium
Tel: +32.2.656. 9120
Fax: +32.2.656. ***8044***
e-mail: dominique.boutriau@gskbio.com

(Amendment 7: 12 April 2006)

Study Contact for Reporting of a Serious Adverse Event for WHO
Dr. Zarifah Reed
WHO/IVR/POP
WHO/IVB/IVR/POP
20 Avenue Appia
CH-1211 GENEVE 27
Switzerland
Tel. +41 22 791 4760
Fax. +41 22 791 4860
Email: reedz@who.int

Back-up Study Contact for Reporting of a Serious Adverse Event at GSK Biologicals
GSK Biologicals Clinical Safety Physician
Tel: +32-2-656 ***88 50***
Fax: +32-2-656 80 09***/5116***
Mobile phone for 7/7 day availability: +32 477 404 713 ***or 32-(0) 474 53 48 68***

(Amendment 7: 12 April 2006)

Study Contact for Emergency Code Break
Local safety monitor i.e.,
Dr. John Williams (who will forward information to IDMC chairman)
Navrongo Health Research Center,
Ministry of Health, PO Box 114, Navrongo, Ghana
Tel: + 233 742 22310/ 22380
Fax: + 233 742 22320
e-mail: jwilliams@navrongo.mimcom.net

Back-up Local Safety Monitor
Dr. Frank Baiden
Navrongo Health Research Center,
Ministry of Health, PO Box 114, Navrongo, Ghana
Tel: + 233 742 22310/ 22380
Fax: + 233 742 22320
e-mail: fbaiden@navrongo.mimcom.net

Study Center
Navrongo Health Research Center
Ministry of Health, PO Box 114, Navrongo, Ghana
Tel: + 233 742 22310/ 22380
Fax: + 233 742 22320

Investigator Agreement

**eTrack study number**	759346/009 (DTPwHB/HIBMenACTT009)<br>104430 (DTPwHB/HIBMenACTT023 BST:009)
**Title**	A phase II, double blind, randomized, controlled study to evaluate the immunogenicity, reactogenicity and safety of GlaxoSmithKline (GSK) Biologicals' Hib-MenAC vaccine, extemporaneously mixed with GSK Biologicals' Tritanrix™-HepB, as compared to GSK Biologicals' Hiberix™ vaccine, extemporaneously mixed with GSK Biologicals' Tritanrix™-HepB, when administered intramuscularly in infants at 6, 10 and 14 weeks of age.

I agree:

- To assume responsibility for the proper conduct of the study at this site.
- To conduct the study in compliance with this protocol, any mutually agreed future protocol amendments, and with any other study conduct procedures provided by GlaxoSmithKline Biologicals (GSK Biologicals).
- Not to implement any changes to the protocol without agreement from the sponsors and prior review and written approval from the Institutional Review Board (IRB) or Independent Ethics Committee (IEC), except where necessary to eliminate an immediate hazard to the subjects, or for administrative aspects of the study (where permitted by all applicable regulatory requirements).
- That I am thoroughly familiar with the appropriate use of the vaccine, as described in this protocol, and any other information provided by the sponsors, including, but not limited to, the following: the current Investigator's Brochure (IB) or equivalent document, IB supplement (if applicable), prescribing information (in the case of a marketed vaccine) and/or Master Data Sheet (if the Master Data Sheet exists and serves as reference document for the vaccine in the case of a marketed vaccine).
- That I am aware of, and will comply with, "Good Clinical Practices" (GCP) and all applicable regulatory requirements.
- That I have been informed that certain regulatory authorities require the sponsor (GSK Biologicals) to obtain and supply, as necessary, details about the investigator's ownership interest in the sponsor or the investigational product, and more generally about his/her financial ties with the sponsor. GSK Biologicals will use and disclose the information solely for the purpose of complying with regulatory requirements.

Hence I:

- Agree to supply GSK Biologicals with any necessary information regarding ownership interest and financial ties (including those of my spouse and dependent children).
- Agree to promptly update this information if any relevant changes occur during the course of the study and for 1 year following completion of the study.
- Agree that GSK Biologicals may disclose any information it has about such ownership interests and financial ties to regulatory authorities.
- Agree to provide GSK Biologicals with an updated Curriculum Vitae and other FDA required documents.

Synopsis

Title A phase II, double blind, randomized, controlled study to evaluate the immunogenicity, reactogenicity and safety of GlaxoSmithKline (GSK) Biologicals' Hib-MenAC vaccine, extemporaneously mixed with GSK Biologicals' Tritanrix™-HepB, as compared to GSK Biologicals' Hiberix™ vaccine, extemporaneously mixed with GSK Biologicals' Tritanrix™-HepB, when administered intramuscularly in infants at 6, 10 and 14 weeks of age.

Indication/Study population Primary vaccination of healthy infants at 6, 10 and 14 weeks of age.

Rationale African countries, and more specifically the countries of the meningitis belt, face regular epidemics of meningococcal disease, including meningitis with an incidence as high as 1000/100,000. Except for an outbreak of serogroup W-135 that spread in Burkina Faso in 2002 and 2003, serogroup A and to a lesser extent serogroup C are largely responsible for *N. meningitidis* epidemics in African countries. Children aged 5-14 years are the most affected. The existing meningococcal plain polysaccharide vaccines are less immunogenic in small children; also the duration of protection induced by these vaccines is short-lived, as polysaccharides vaccine do not induce immune memory. Therefore, there is a need to develop new generation meningococcal vaccines that protect against meningococcal disease in early childhood and induce immune memory.

Recently, meningococcal serogroup C conjugate vaccines have been developed and have proven effective in small children and infants.

In order to protect children from early childhood against *N. meningitidis* of the two most prevalent serogroups in Africa, and to facilitate the incorporation of such a vaccine in the immunization calendar, GSK Biologicals is developing a combined Hib-MenAC conjugate vaccine to be extemporaneously mixed with the Tritanrix™-HepB (DTPw-HBV) vaccine for administration as a 3-dose primary vaccination course in infants aged 6 weeks and over.

The new Tritanrix™-HepB/Hib-MenAC vaccine has been evaluated in a feasibility trial in the Philippines. The combination using the Hib-MenAC formulation with a dosage of 2.5 µg of each of the MenA, MenC and Hib

conjugate antigens has been selected for further clinical development: the selected formulation was well tolerated; it induced similar antibody levels against the diphtheria, tetanus, pertussis, hepatitis B, Hib and meningococcal serogroup C antigens as the benchmark vaccines (Tritanrix™-HepB/Hiberix™ and Meningitec™) and 97.7 % subjects had meningococcal serogroup A serum bactericidal antibody titres of at least 1:8. As feasibility of the new vaccine has now been shown, the present study plans to evaluate the use of GSK Biologicals' Tritanrix™-HepB/Hib-MenAC vaccine in a high endemicity region in Africa.

The study will evaluate the immunogenicity induced by three doses of the vaccine given at 6, 10 and 14 weeks of age by measuring the immune response one month post-vaccination to ensure the vaccine is effective in the youngest children when the disease burden is highest. In order to assess whether the vaccine has induced long-term protection, the persistence of the immune response will be measured when the child is one year of age, and a small dose of plain polysaccharide (1/5th of a dose of Mencevax™ AC, i.e. 10µg/polysaccharide) will be given to evaluate whether immune memory was induced by priming.

Objectives **Co-primary**

One month after primary vaccination:

- To demonstrate the immunogenicity of Tritanrix™-HepB/Hib-MenAC with respect to SBA-MenA and SBA-MenC.
- To demonstrate that Tritanrix™-HepB/Hib-MenAC is non-inferior to the control vaccine Tritanrix™-HepB/ Hiberix™ with respect to the immunogenicity of all common antigens (anti-PRP, anti-Diphtheria, anti-Tetanus, anti-BP, and anti-HBs).

Criteria to achieve the co-primary objectives:

- Immunogenicity of SBA-MenA and SBA-MenC: lower limit of the 95% CI for the percentage of subjects with SBA-MenA titers (respectively SBA-MenC titers) ≥ 1:8 is greater than 80%.
- Non-inferiority for all common antigens: upper limit of the 95% CI for the group difference in terms of the percentage of subjects with anti-PRP concentration ≥ 1 µg/ml (respectively, anti-diphtheria concentration ≥ 0.1 IU/ml, anti-tetanus concentration ≥ 0.1 IU/ml, anti-HBs concentration ≥ 10 mIU/ml) is below 10% and upper limit of the 95% CI for the group GMC ratio in anti-BP is below 1.5.

Secondary

- At 12 months of age, to evaluate the antibody persistence induced by the primary vaccination with Tritanrix™-HepB/Hib-MenAC (new vaccine) and Tritanrix™-HepB/ Hiberix™ (control vaccine) with respect to the immunogenicity of all antigens administered.
- At 12 months of age, to evaluate the immune memory induced by the primary vaccination with Tritanrix™-HepB/Hib-MenAC vaccine by administering 10 µg of each meningococcal A and C polysaccharide (one fifth of a dose of Mencevax™ AC), using the unprimed subjects of the Tritanrix™-HepB/ Hiberix™ group as control.
- To assess the reactogenicity and safety of the primary vaccination after each vaccine dose and overall in the two study groups.
- At 12 months of age, to assess the reactogenicity and safety of the 10 µg meningococcal A and C polysaccharides when given to subjects primed with either Tritanrix™-HepB/Hib-MenAC or with Tritanrix™-HepB/ Hiberix™.

Study design

- Experimental design: 2 parallel groups of 140 subjects each to receive either of the following vaccines:
  - Tritanrix™-HepB/Hib-MenAC
  - Tritanrix™-HepB/Hiberix™
- Active control group (Tritanrix™-HepB/Hiberix™)
- Indication: primary vaccination of infants according to the Expanded Program of Immunization (EPI) schedule (6, 10, 14 weeks of age).
- BCG and OPV vaccines can be given at birth or up to at least 2 weeks before study start. OPV can then be given concomitantly with the study vaccines at 6, 10 and 14 weeks of age or at any time during the study, whichever is more appropriate. At 9 months of age, all subjects will receive measles and yellow fever vaccines.
- At 12 months of age, all subjects will be evaluated for antibody persistence and will be administered 10 µg of each plain meningococcal serogroup A and C polysaccharides (one fifth of a dose of Mencevax™ AC) to evaluate the immune memory induced by vaccination.
- After study end, one dose of Mencevax™ ACWY will be offered to the Tritanrix™-HepB/Hiberix™ control group to protect them against meningococcal diseases of these serogroups: Mencevax™ ACWY will be given when the child is two years of age, or, if a meningitis epidemic occurs, earlier in the second year of life.

- Treatment allocation: randomized (1:1)
- Blinding: Double-blind
- Study duration per subject: approximately ***22.5 months to include the retrospective follow-up of SAE(s) up to the time that subjects are at least 24 months of age.***
- Data collection: Hard copy Case Report Form (CRF)
- For all subjects: 4 blood samples (3.5 ml):
  - immediately before the first dose of the primary vaccination course
  - one month after the third dose of the primary vaccination course
  - just prior to administration of the plain polysaccharides of serogroups A and C
  - one month after the administration of the plain polysaccharides of serogroups A and C.
- 4-day follow-up of solicited local and general symptoms after each dose.
- One month follow-up for unsolicited symptoms after each dose.
- Reporting of serious adverse events during the entire study period.
- ***Retrospective follow-up of SAE(s). The parents/guardians of study subjects will be contacted by the local health staff when their child is at least 24 months of age for a follow-up of SAE(s) (i.e. any SAE(s) that occurred since the last study visit [Visit 7] up to the time that subjects are at least 24 months of age).***

(Amendment 7: 12 April 2006)

Study Population

- Healthy infants aged 6-8 weeks.
- 280 in total (140/group) to allow 220 evaluable subjects for the co-primary endpoints.

Co-primary endpoints

Immunogenicity: post-primary vaccination:
In all subjects, one month after the 3rd dose of the primary vaccination:

- Serum bactericidal antibody titer (MenC) ≥1:8 (seropositivity)
- Serum bactericidal antibody titer (MenA) ≥1:8 (seropositivity)
- Anti-PRP concentration ≥ 1 µg/ml (seroprotection)
- Anti-HBs concentration ≥10 mIU/ml (seroprotection)
- Anti-tetanus concentration ≥ 0.1 IU/ml (seroprotection)
- Anti-diphtheria concentration ≥ 0.1 IU/ml by ELISA and, if negative, ≥ 0.016 IU/ml by Vero-cell (seroprotection)

- Concentration of anti-BP antibodies

Secondary endpoints

Immunogenicity

Pre-primary vaccination

In all subjects, just prior to the administration of the 1st dose of the primary vaccination

- SBA-MenA titer ≥ 1:8 (seropositivity)
- SBA-MenC titer ≥ 1:8 (seropositivity)
- Anti-PSA concentration ≥ 0.3 µg/ml (seropositivity)
- Anti-PSC concentration ≥ 0.3 µg/ml (seropositivity)
- Anti-PSA concentration ≥ 2 µg/ml
- Anti-PSC concentration ≥ 2 µg/ml
- Anti-diphtheria concentration ≥ 0.1 IU/ml (seroprotection)
- Anti-HBs concentration ≥10 mIU/ml (seroprotection)
- Anti-BP concentration ≥ 15 EL.U/ml (seropositivity)
- Concentration/titers of SBA-MenA, SBA-MenC, anti-PSA, anti-PSC, anti-HBs, anti-diphtheria and anti-BP antibodies

Post-primary vaccination:

In all subjects, one month after the 3rd dose of the primary vaccination:

- Anti-PSA concentration ≥ 0.3 µg/ml (seropositivity)
- Anti-PSC concentration ≥ 0.3 µg/ml (seropositivity)
- Anti-PSA concentration ≥ 2 µg/ml
- Anti-PSC concentration ≥ 2 µg/ml
- Anti-PRP concentration ≥ 0.15 µg/ml (seroprotection)
- Anti-BP concentration ≥15 EL.U/ml
- Titers/Concentrations of all antibodies except anti-BP (see co-primary endpoints)

Antibody persistence:

In all subjects, at twelve months of age, just prior to the administration of 1/5 of a dose of Mencevax™ AC:

- SBA-MenA titer ≥ 1:8 (seropositivity)
- SBA-MenC titer ≥ 1:8 (seropositivity)
- Anti-PSA concentration ≥ 0.3 µg/ml (seropositivity)
- Anti-PSC concentration ≥ 0.3 µg/ml (seropositivity)
- Anti-PSA concentration ≥ 2 µg/ml
- Anti-PSC concentration ≥ 2 µg/ml
- Anti-PRP concentration ≥ 0.15 µg/ml (seroprotection)
- Anti-PRP concentration ≥ 1 µg/ml (seroprotection)

- Anti-HBs concentration ≥10 mIU/ml (seroprotection)
- Anti-tetanus concentration ≥ 0.1 IU/ml (seroprotection)
- Anti-diphtheria concentration ≥ 0.1 IU/ml by ELISA and, if negative, ≥ 0.016 IU/ml by Vero-cell (seroprotection)
- Anti-BP concentration ≥15 EL.U/ml (seropositivity)
- Concentration/titers of SBA-MenA, SBA-MenC, anti-PSA, anti-PSC, anti-PRP, anti-HBs, anti-tetanus, anti-diphtheria and anti-BP antibodies.

Immune memory:
In all subjects, one month after the administration of 1/5 of a dose of Mencevax™ AC:

- SBA-MenA titer ≥ 1:8 (seropositivity)
- SBA-MenC titer ≥ 1:8 (seropositivity)
- Anti-PSA concentration ≥ 0.3 µg/ml (seropositivity)
- Anti-PSC concentration ≥ 0.3 µg/ml (seropositivity)
- Anti-PSA concentration ≥ 2 µg/ml
- Anti-PSC concentration ≥ 2 µg/ml
- Concentration/titers of SBA-MenA, SBA-MenC, anti-PSA and anti-PSC antibodies.

Reactogenicity and safety:
After each vaccine dose:

- Occurrence of solicited symptoms (during the 4-day follow-up period)
  Local: Pain and swelling at the injection site
  General: Drowsiness, Fever, Irritability/ Fussiness, Loss of appetite
- Occurrence of unsolicited symptoms (during the 30-day follow-up period)

Over the full course of the study:

- Occurrence of serious adverse events

Table of Contents

1 Introduction 22
1.1 Background 22
1.2 GSK Biologicals' combined *H. influenzae* type b - *N. meningitidis* polysaccharide A, C (Hib-MenAC) conjugated vaccine 24
1.3 Rationale for the study 24
1.4 Study site 25

2 Objectives 27
2.1 Co-primary objectives 27
2.2 Secondary objectives 28

3 Study Design Overview 29

4 Study Cohort 31
4.1 Number of subjects 31
4.2 Inclusion criteria 31
4.3 Exclusion criteria for enrolment 32
4.4 Elimination criteria during the study 33
4.5 Contraindications to subsequent vaccination 33

5 Conduct of Study 35
5.1 Ethics and regulatory considerations 35
5.1.1 Institutional Review Board/Independent Ethics Committee (IRB/IEC) 36
5.1.2 Informed consent 37
5.1.3 Independent Data Monitoring Committee (IDMC) 39
5.2 General study aspects 41
5.3 Outline of study procedures 43
5.4 Detailed description of study stages/visits 45
5.5 Sample handling and analysis 53
5.5.1 Treatment and storage of biological samples 53
5.5.2 Laboratory assays 54
5.5.3 Serology plan 55
5.5.4 Endpoints for suboptimal response 56

6 Investigational Products and Administration 57
6.1 Study Vaccines 57
6.1.1 GSK Biologicals' *Haemophilus influenzae* type b meningococcal AC-TT (Hib-MenAC) conjugate vaccine 57
6.1.2 GSK Biologicals' combined diphtheria-tetanus-whole cell *Bordetella pertussis*-hepatitis B (DTPw-HBV) vaccine: Tritanrix™-HepB 57
6.1.3 GSK Biologicals' *Haemophilus influenzae* type b conjugate vaccine:

Hiberix™ 58
6.1.4 GSK Biologicals' Mencevax™ AC vaccine, used as booster vaccine.......58
6.1.5 Commercial Measles and Yellow Fever vaccines....................59
6.1.6 Dosage and administration....................59
6.1.7 Extemporaneous mixing of Tritanrix™-HepB and Hib-MenAC vaccines or Hiberix™....................60
6.2 Storage....................61
6.3 Treatment allocation and randomization....................61
6.4 Method of blinding and breaking the study blind....................62
6.5 Replacement of unusable vaccine doses....................62
6.6 Packaging....................63
6.7 Vaccine accountability....................63
6.8 Concomitant medication/treatment....................63
6.8.1 Recording of concomitant medication/treatment during the primary phase of the study....................63
6.8.2 Recording of concomitant medication/treatment during the booster phase of the study....................64
7 Health Economics.................... 65
8 Adverse Events, Serious Adverse Events and vaccine failure 65
8.1 Definition of an adverse event....................66
8.2 Definition of a serious adverse event....................67
8.3 Lack of efficacy....................68
8.4 Clinical laboratory parameters and other abnormal assessments qualifying as adverse events and serious adverse events....................68
8.5 Time period, frequency, and method of detecting adverse events and serious adverse events....................69
8.5.1 Solicited adverse events....................70
8.6 Evaluating adverse events and serious adverse events....................71
8.6.1 Assessment of intensity....................71
8.6.2 Assessment of causality....................73
8.7 Follow-up of adverse events and serious adverse events and assessment of outcome....................74
8.8 Prompt reporting of serious adverse events to GSK Biologicals, WHO and the local safety monitor....................76
8.8.1 Time frames for submitting serious adverse event reports to GSK Biologicals, WHO and the local safety monitor....................76
8.8.2 Completion and transmission of serious adverse event reports to GSK Biologicals, WHO and the local safety monitor....................76
8.9 Regulatory reporting requirements for serious adverse events....................79

8.10 Post study adverse events and serious adverse events....79
8.11 Treatment of adverse events....80

9 Subject Completion and Withdrawal 80
9.1 Subject completion....80
9.2 Subject withdrawal....80
9.2.1 Subject withdrawal from the study....80
9.2.2 Subject withdrawal from investigational product 81
9.3 Extension study 81
9.4 Screen and baseline failures 82

10 Data Evaluation: Criteria for Evaluation of Objectives 82
10.1 Co-primary endpoints....82
10.2 Secondary endpoints....82
10.3 Estimated sample size 84
10.4 Study cohorts to be evaluated....85
10.5 Derived and transformed data 87
10.6 Final analyses for the primary vaccination course....88
10.6.1 Analysis of demographics/baseline characteristics 88
10.6.2 Analysis of Immunogenicity....88
10.6.3 Analysis of Safety....89
10.7 Final Analyses for the booster vaccination....90
10.7.1 Analysis of immunogenicity....90
10.7.2 Analysis of safety 91
10.8 Reporting of final analyses....92
10.9 Planned interim analysis....92

11 Administrative Matters.... 93

Appendices

List of Abbreviations

AE	Adverse event
ATP	According to protocol
BCG	Bacille Calmette-Guérin
BP	Bordetella Pertussis Antibodies
CDM	Clinical Development Manager
CI	Confidence Interval
CPMS	Clinical Programs Monitoring System
CRA	Clinical Research Associate
CRF	Case report form
CRM_{197}	A non-toxic mutant form of *Corynebacterium diphtheria* toxin
CSC	Central Study Coordinator
DT	Diphtheria toxoid
DTPw-HBV	Combined Diphtheria, Tetanus, Whole Cell Pertussis and Hepatitis B Vaccine
EISR	Expedited Investigator Safety Reports
ELISA	Enzyme-linked immunosorbent assay
EL.U	ELISA unit
EPI	Expanded Program of Immunization
GCP	Good Clinical Practice
GMC/GMT	Geometric mean concentration/Geometric mean titer
GSK	GlaxoSmithKline
HBs	Hepatitis B surface antigen
HBV	Hepatitis B virus
Hib	*Haemophilus influenzae* type b
Hiberix™	GSK Biologicals' *Haemophilus influenzae* type b conjugate vaccine
Hib-MenAC	GSK Biologicals' *Haemophilus influenzae* type b - meningococcal AC-tetanus toxoid conjugate (vaccine)
HIV	Human immunodeficiency virus
IB	Investigator Brochure
IDMC	Independent Data Monitoring Committee
IEC	Independent ethics committee
IRB	Institutional Review Board
IU	International unit
KND	Kassena-Nankana District
Lf	Limes flocculation
LSM	Local Safety Monitor

MedDRA	Medical Dictionary for Regulatory Activities
µg	Microgram
mg	Milligram
MHC	Major Histocompatibility Complex
ml	Milliliter
NDSS	Navrongo Demographic Surveillance System
NHRC	Navrongo Health Research Center
OPV	Oral polio vaccine
PID	Patient identification number
PRP	Polyribosil ribitol phosphate
PSA	Polysaccharide A
PSC	Polysaccharide C
RAP	Report Analysis Plan
RDE	Remote data entry
SAE	Serious adverse event
SBA	Serum bactericidal assay/activity
SBA-MenA	Serum bactericidal assay/activity against *N. meningitidis* serogroup A
SBA-MenC	Serum bactericidal assay/activity against *N. meningitidis* serogroup C
SIDS	Sudden infant death syndrome
SOP	Standard operating procedure
STI	Swiss Tropical Institute
Tritanrix™-HepB	GSK Biologicals' combined diphtheria, tetanus, whole cell pertussis, hepatitis B vaccine
TT/T	Tetanus toxoid
VERO	African monkey kidney cells
WHO	World Health Organization
WMH	War Memorial Hospital

Glossary of Terms

**Adverse Event:**	Any untoward medical occurrence in a patient or clinical investigation subject administered a pharmaceutical product and which does not necessarily have a causal relationship with the pharmaceutical product (as defined by ICH Guideline for Good Clinical Practice).
**Blinding:**	A procedure in which one or more parties to the trial are kept unaware of the treatment assignment in order to reduce the risk of biased study outcomes. In a single-blind trial, the investigator and/or his staff are aware of the treatment assignment but the subject is not. When the investigator and sponsor staff who are involved in the treatment or clinical evaluation of the subjects and review/analysis of data are also unaware of the treatment assignments, the study is double blind. This level of blinding is maintained throughout the conduct of the trial, and only when the data are cleaned to an acceptable level of quality will appropriate personnel be unblinded or when required in case of a serious adverse event.
**Eligible:**	Qualified for enrolment into the study based upon strict adherence to inclusion/exclusion criteria.
**Evaluable:**	Meeting all eligibility criteria, complying with the procedures defined in the protocol, and, therefore, included in the according-to-protocol (ATP) analysis (see Sections 4.4 and 10.4 for details on criteria for evaluability).
**eTrack**	GlaxoSmithKline’s clinical trials tracking tool
**Investigational Product:**	A pharmaceutical form of an active ingredient or placebo being tested or used as a reference in a clinical trial, including a product with a marketing authorization when used in a way different from the approved form, or when used for an unapproved indication, or when used to gain further information about an approved use.
**Protocol Amendment:**	Any change in a clinical protocol which affects the safety of subjects, the scope, design, assessments or scientific

	validity of the clinical investigation, e.g., dose change, duration of treatment, number of subjects, control group(s), the assessments.
**Protocol Modification:**	Strictly, any change to a clinical protocol that is not considered to be a protocol amendment. In essence, changes to clarify (but not alter) existing design features of a clinical investigation or to encourage greater compliance with the intent of the clinical protocol may be issued as modifications rather than amendments. A protocol modification addresses logistical or administrative aspects of the study (e.g., change of monitor(s), telephone number(s).
**Site Monitor:**	An individual assigned by GSK Biologicals who is responsible for assuring proper conduct of a clinical study at one or more investigational sites.
**Local Safety Monitor:**	The overall role of the Local Safety Monitor, an experienced physician based in-country, will be to support the study investigators and to act as a link between the investigators and the Independent Data Monitoring Committee (IDMC) (see Section 5.1.3 for further details).
**Solicited Adverse Event:**	Adverse events (AEs) to be recorded as endpoints in the clinical study. The presence/occurrence/intensity of these events is actively solicited from the subject or an observer during a specified post-vaccination follow-up period.
**Study Monitor:**	An individual assigned by and centrally located at GSK Biologicals who is responsible for assuring proper conduct of a clinical study, i.e., Central Study Coordinator.
**Subject(s):**	Term used throughout the protocol to denote the enrolled individual(s).
**Unsolicited Adverse Event:**	Any adverse event (AE) reported in addition to those solicited during the clinical study. Also any “solicited” symptom with onset outside the specified period of follow-up for solicited symptoms will be reported as an unsolicited adverse event.

1 Introduction

Meningococcal disease, including meningitis and meningococcaemia (meningococcal septicaemia), usually follows invasive infection by *Neisseria meningitidis* (meningococcus) and is a major cause of death and morbidity throughout the world. Despite the availability of appropriate treatment, the mortality rate is still 7-19% [1]. The risk of meningococcal disease is inversely related to age with 49% of cases occurring in children of four years and younger in Europe [2]. However, older children, adolescents and adults are more often affected during epidemics [3,4].

The disease is endemic in developed countries, such as the US and Europe, and developing countries [5]. Epidemics occur regularly worldwide with highest attack rates in sub-Saharan countries. Serogroups A, B, C, W-135 and Y are the most common causes of invasive meningococcal disease worldwide; serogroups B and C account for most of the cases in Europe and Latin America, while B, C and Y are the 3 prevalent serogroups in the USA. In Asia, serogroups A and C are most common [2,5,6].

African countries, and more specifically the countries of the meningitis belt, face regular epidemics of meningococcal disease, including meningitis with incidence as high as 1000/100,000 and a case-fatality rate of 10 % but much higher death rates are reported in remote areas where the population have a poor access to health care services. Except for outbreaks of serogroup W-135 that spread in Burkina Faso in 2002 and 2003, serogroup A and to a less extent serogroup C are the major serogroups responsible for *N. meningitidis* epidemics in African countries. Outbreaks hit all age-groups, children aged 5-14 years being the most affected [7].

The disease presentation may be fulminant with no time for antibiotics to be effective, and permanent neurological damage can occur in children after infection, despite antibiotic therapy. Meningococcal strains with relative or absolute resistance to penicillin have been reported in many countries. In Africa, oily chloramphenicol is the drug of choice and although no cases of resistance have been reported in Africa, some few cases have been reported in Vietnam.

1.1 Background

Currently available meningococcal vaccines consisting of pure capsular polysaccharides (serogroup A, C, W-135 and Y meningococcal vaccines) are safe and efficacious in adults and children over two years of age. However they do not induce a satisfactory response in infants and young children under two years of age, the age group most at risk of

meningococcal infections. Indeed in this age group, maturation of B-cells is incomplete which leads to a defective B-cell activation. In addition, polysaccharide vaccines do not reduce mucosal carriage and cannot consequently confer herd immunity; they do not induce immunological memory and the associated affinity/avidity maturation of antibodies.

Polysaccharide antigens can however be made to induce a T-cell response and immunological memory, by their covalent coupling with proteins which, in association with Major Histocompatibility Complex (MHC) class II molecules, will be presented to T-helper cells on the surface of Antigen Presenting Cells. This principle was the key to the successful development of *Haemophilus influenzae* type b (Hib)-conjugate vaccines immunogenic in young children and able to induce immunological memory.[8,9,10,11]

To date, vaccine manufacturers have been developing meningococcal conjugate vaccines using the same carrier proteins as the one they use for their Hib conjugate vaccine. Based on this principle, meningococcal serogroup C conjugate vaccines using a non-toxic mutant of diphtheria toxin (CRM_{197}) or tetanus toxoid (TT) as carrier proteins have been recently licensed in Europe, Canada, Australia and Latin America. The first data available after the introduction of the meningococcal serogroup C conjugate vaccines in a mass vaccination campaign in the United Kingdom have proven the effectiveness of these vaccines in all age-groups including infants [12,13,14].

N. meningitidis of serogroup A being important in Africa, meningococcal serogroup A + C conjugate vaccines using carrier proteins derived from the diphtheria toxoid have been developed and evaluated in clinical trials in African countries such as Niger and The Gambia. They were found to be safe in these populations. [15,16].

The immunogenicity of meningococcal conjugate vaccines in infants is best shown by measuring bactericidal antibodies one month after primary vaccination. Demonstrating that meningococcal serogroup A and C conjugate vaccines prime for immune memory is an important mechanism to support the long-term protection effect of these vaccines. Such priming for memory was shown for the meningococcal serogroup C conjugate vaccine [17]and for a preliminary meningococcal serogroup A+C conjugate candidate vaccine evaluated in the United Kingdom [18]. However, in another trial in the Gambia, strong memory to the C component was induced but evidence of memory induction for the A component was less convincing [15]. Priming for memory was demonstrated by administering a small dose (10 µg) of unconjugated polysaccharide C six to twelve months after priming. The dose of 10 µg polysaccharide was selected after three adverse events (allergic reactions) had been reported in 10 subjects initially vaccinated with the full polysaccharide vaccine (50 µg/polysaccharide). No further allergic reactions were reported with the reduced dose of 10 µg [18].

1.2 GSK Biologicals' combined *H. influenzae* type b - *N. meningitidis* polysaccharide A, C (Hib-MenAC) conjugated vaccine

GSK Biologicals is developing a combined Hib-MenAC conjugate vaccine, where each polysaccharide (PRP, PSA, PSC) is covalently linked to the tetanus toxoid (-TT) carrier protein.

In a previous set of studies, GSK Biologicals had evaluated a Hib-MenAC conjugate vaccine (the meningococcal serogroup A and C polysaccharides were conjugated to another carrier protein than tetanus toxoid) and this combination, given as such or extemporaneously mixed with the Tritanrix™-Hep B vaccine was found to be safe (total N: 259 infants).

Recently, GSK Biologicals has developed a MenC and a Hib-MenC conjugate vaccine using also –TT as carrier protein. Both vaccines were shown to be safe and immunogenic in infants.

In the first three-dose primary vaccination study performed in the Philippines, three formulations of the combined Hib-Men AC vaccine (using TT as carrier protein) each extemporaneously mixed with Tritanrix™-HepB were evaluated and the Tritanrix™-HepB/Hib-MenAC combination using the Hib-MenAC formulation with a dosage of 2.5 µg of each of the MenA, MenC and Hib conjugate antigens was selected for further clinical development: the selected formulation was well tolerated; it induced similar antibody levels against the diphtheria, tetanus, pertussis, hepatitis B, Hib and meningococcal serogroup C antigens as the benchmark vaccines (Tritanrix™-HepB/Hiberix™ and Meningitec™) and 97.7 % subjects had meningococcal serogroup A serum bactericidal antibody titres of at least 1:8.

Pre-clinical and clinical data generated with GSK Biologicals' Tritanrix™-HepB/Hib-MenAC vaccine are available in the combined Diphtheria-Tetanus-whole cell Pertussis-Hepatitis B, Haemophilus Influenzae type B and Neisseria meningitidis serogroup A and C-Tetanus toxoid conjugate vaccine Investigator Brochure (DTPw-HBV-Hib-MenAC, 1st Edition, January 2004).

1.3 Rationale for the study

African countries, and more specifically the countries of the meningitis belt, face regular epidemics of meningococcal disease, including meningitis with an incidence as high as 1000/100,000. Except for an outbreak of serogroup W-135 that spread in Burkina Faso in 2002 and 2003, serogroup A and to a lesser extent serogroup C are largely responsible for *N. meningitidis* epidemics in African countries. Children aged 5-14 years are the most

affected. The existing meningococcal plain polysaccharide vaccines are less immunogenic in small children; also the duration of protection induced by these vaccines is short-lived as polysaccharides vaccine do not induce immune memory. Therefore, there is a need to develop new generation meningococcal vaccines that protect against meningococcal disease in early childhood and induce immune memory.

Recently, meningococcal serogroup C conjugate vaccines have been developed and have proven effective in small children and infants.

In order to protect children from early childhood against *N. meningitidis* of the two most prevalent serogroups in Africa, and to facilitate the incorporation of such a vaccine in the immunization calendar, GSK Biologicals is developing a combined Hib-MenAC conjugate vaccine to be extemporaneously mixed with the Tritanrix™-HepB vaccine for administration as a 3-dose primary vaccination course in infants aged 6 weeks and over.

The new Tritanrix™-HepB/Hib-MenAC vaccine has been evaluated in a feasibility trial in the Philippines. The combination using the Hib-MenAC formulation with a dosage of 2.5 µg of each of the MenA, MenC and Hib conjugate antigens has been selected for further clinical development: the selected formulation was well tolerated; it induced similar antibody levels against the diphtheria, tetanus, pertussis, hepatitis B, Hib and meningococcal serogroup C antigens as the benchmark vaccines (Tritanrix™-HepB/Hiberix™ and Meningitec™) and 97.7 % subjects had meningococcal serogroup A serum bactericidal antibody titres of at least 1:8. As feasibility of the new vaccine ~~is~~ has now been shown, the present study plans to evaluate the use of GSK Biologicals' Tritanrix™-HepB/Hib-MenAC vaccine in a high endemicity region in Africa.

The study will evaluate the immunogenicity induced by three doses of the vaccine given at 6, 10 and 14 weeks of age by measuring the immune response one month post-vaccination to ensure the vaccine is effective in the youngest children when the disease burden is highest. In order to assess whether the vaccine has induced long-term protection, the persistence of the immune response will be measured when the child is one year of age, and a small dose of plain polysaccharide (1/5th of a dose of Mencevax™ AC, i.e. 10µg/polysaccharide) will be given to evaluate whether immune memory was induced by priming.

1.4 Study site

The Kassena-Nankana district (KND) is within the guinea savannah area of northern Ghana bordering Burkina Faso. The entire district lies within the meningitis belt of sub-Saharan Africa. The climate is that of the sub-Sahel with a short rainy season from May

to October (average annual rainfall 850-950 mm) and a long dry season from November to April. The district covers an area of 1,675 km^2 with a population of about 140,000. The entire population of the district is included in a demographic surveillance system, the Navrongo Demographic Surveillance System (NDSS), in which births, deaths, in and out migrations and other demographic parameters are recorded in a database and updated every ninety days.[19]

There are four health centers in the district, one each in the northern, southern, eastern and western parts of the district. The district hospital, the War Memorial Hospital (WMH), is situated in the central part of the district. It is a 120-bed hospital. Immunization services are conducted in all the health facilities as well as outreach clinics. The Expanded Programme of Immunization (EPI) calendar is as follows: BCG and OPV are given at birth. Tritanrix™-HepB/Hib (DTPw-HepB/Hib) and OPV vaccines are given as 3 doses at 6, 10 and 14 weeks of age. Measles and yellow fever vaccines are given at 9 months.

The Navrongo Health Research Center (NHRC) has the mandate to research into health problems facing the people of the Sahelian ecological belt with a view to informing policy decisions. Over the years, it has conducted research on Vitamin A supplementation, use of impregnated bednets, Community Health and Family planning services, malaria, diarrhoea and meningitis. The findings from many of these studies have been made into policies in the country.

Ongoing work on meningitis in collaboration with the Swiss Tropical Institute, Basel, Switzerland has involved studies on carriage [20, 21], risk factors[22], survival and sequelae[23] and aetiological agents of meningitis in the district [24].

Coordination with the EPI

The vaccine trial will be conducted at several EPI clinics in the Kassena-Nankana district where routine immunization is taking place.

All vaccinees in the EPI system have the so-called “Growth Monitoring Cards” on which the vaccines they receive are recorded. The subjects will be recruited at the time they are to receive the routine DTP, Hib and HepB vaccines (at 6 weeks of age). The vaccines given will be recorded on a specific immunization card (yellow card) and also on the Growth Monitoring Cards, i.e. the name of the study vaccine will be recorded in the box where DTP, Hib and HepB vaccines are usually recorded. Therefore, a duplication of vaccination is prevented as the Growth Monitoring Cards are always brought to the EPI clinics by the parents/guardians.

Health indicators

1. Infant Mortality Rate 95.5 per 1000 live births[25]

2. Crude death rate 16.2 deaths per 1000 person years [25]

3. The three top causes of death in the WMH in 2000 were:
   i. Malaria
   ii. Anaemia
   iii. Pneumonia

Malaria is the leading cause of both morbidity and mortality, accounting for 60% of hospital admissions and 41% of hospital deaths.
Data from verbal autopsies coded between 1993 and 1999, document malaria as responsible for 35% of all deaths in children aged less than 5 years.

4. The 1996/97 meningococcal disease epidemic in the district, resulted in 1,396 cases with 65 deaths [26].

5. The EPI coverage for Diphtheria-Tetanus-Pertussis 3rd dose in the district is 70%.

6. The HIV prevalence amongst antenatal attendants is 2.4% only.

2 Objectives

2.1 Co-primary objectives

One month after primary vaccination:

- To demonstrate the immunogenicity of Tritanrix™-HepB/Hib-MenAC with respect to SBA-MenA and SBA-MenC.
- To demonstrate that Tritanrix™-HepB/Hib-MenAC is non-inferior to the control vaccine Tritanrix™-HepB/ Hiberix™ with respect to the immunogenicity of all common antigens (anti-PRP, anti-Diphtheria, anti-Tetanus, anti-BP, and anti-HBs).

Criteria to achieve the co-primary objectives:

- Immunogenicity of SBA-MenA and SBA-MenC: lower limit of the 95% CI for the percentage of subjects with SBA-MenA titers (respectively SBA-MenC titers) ≥ 1:8 is greater than 80%.
- Non-inferiority for all common antigens: upper limit of the 95% CI for the group difference in terms of the percentage of subjects with anti-PRP concentration ≥ 1 µg/ml (respectively, anti-diphtheria concentration ≥ 0.1 IU/ml, anti-tetanus

concentration ≥ 0.1 IU/ml, anti-HBs concentration ≥ 10 mIU/ml) is below 10% and upper limit of the 95% CI for the group GMC ratio in anti-BP is below 1.5.

Refer to Section 10.1 for definition of the co-primary endpoints.

2.2 Secondary objectives

- At 12 months of age, to evaluate the antibody persistence induced by the primary vaccination with Tritanrix™-HepB/Hib-MenAC (new vaccine) and Tritanrix™-HepB/ Hiberix™ (control vaccine) with respect to the immunogenicity of all antigens administered.
- At 12 months of age, to evaluate the immune memory induced by the primary vaccination with Tritanrix™-HepB/Hib-MenAC vaccine by administering 10 µg of each meningococcal A and C polysaccharide (one fifth of a dose of Mencevax™ AC), using the unprimed subjects of the Tritanrix™-HepB/ Hiberix™ group as control.
- To assess the reactogenicity and safety of the primary vaccination after each vaccine dose and overall in the two study groups.
- At 12 months of age, to assess the reactogenicity and safety of the 10 µg meningococcal A and C polysaccharides when given to subjects primed with either Tritanrix™-HepB/Hib-MenAC or with Tritanrix™-HepB/ Hiberix™.

Refer to Section 10.2 for definitions of secondary endpoints.

3 Study Design Overview

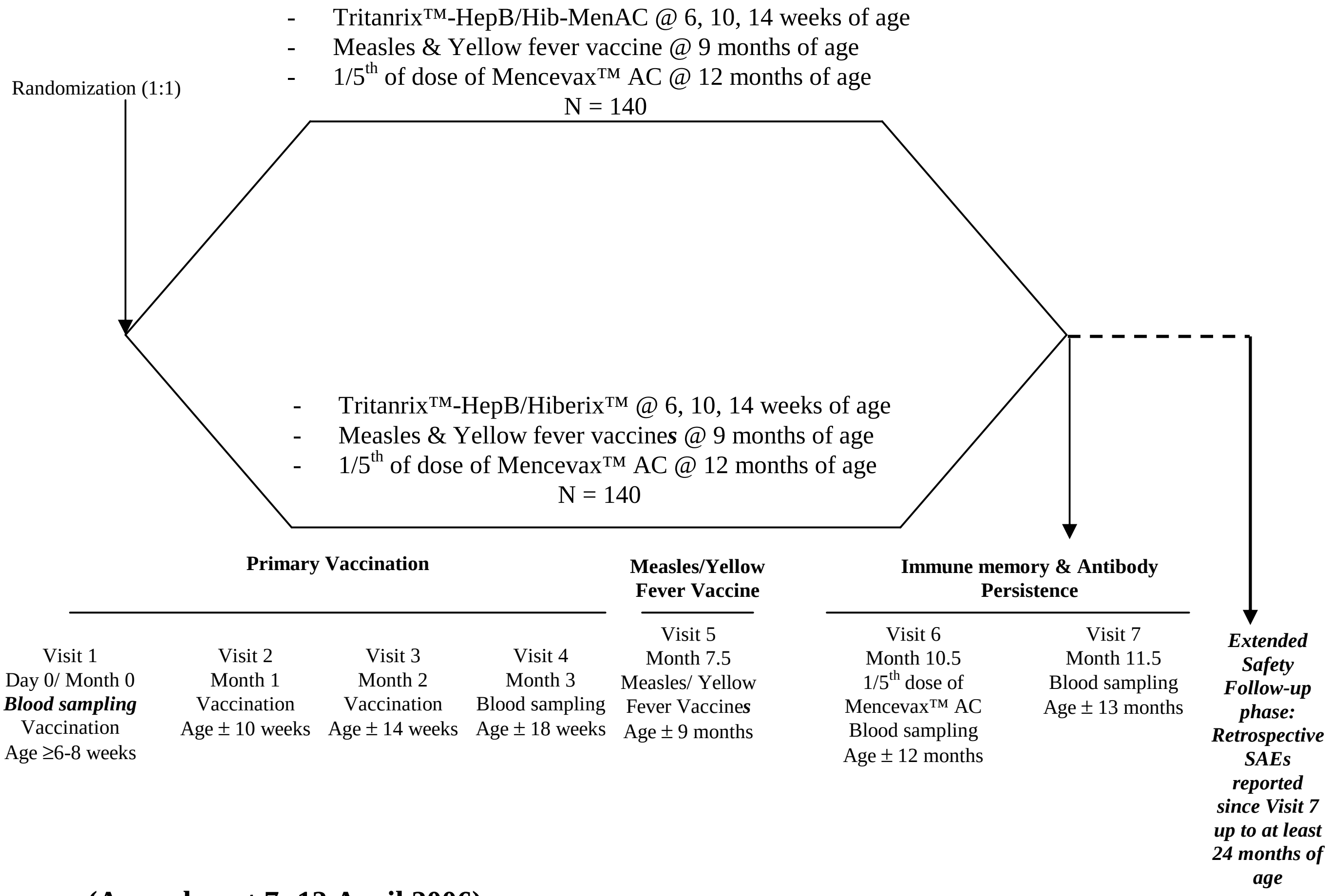

(Amendment 7: 12 April 2006)

Note: BCG and OPV vaccines can be given at birth (or up to at least 2 weeks before the subject's first visit) according to the nationally recommended immunization schedule. OPV can also be given at 6, 10, 14 weeks of age concomitantly with the study vaccines or at any time during the study, whichever is more appropriate.

- Experimental design: 2 parallel groups of 140 subjects each to receive either of the following vaccines**:**

- Tritanrix™-HepB/Hib-MenAC
- Tritanrix™-HepB/Hiberix™

- Indication: primary vaccination of infants according to the Expanded Program on Immunization (EPI) schedule (6, 10, 14 weeks of age).

- To comply with the Ghanean Immunisation Calendar, at 9 months of age, all subjects will receive measles and yellow fever vaccine***s***.

- At 12 months of age, all subjects will be evaluated for antibody persistence and will be administered 10 µg of each plain meningococcal serogroup A and C polysaccharides (one fifth of a dose of Mencevax™ AC) to evaluate the immune memory induced by vaccination.

- Active control group (Tritanrix™-HepB/Hiberix™)

- As the control group will not be properly immunised with 1/5th of a dose of Mencevax™ AC, after study end, one dose of Mencevax™ ACWY will be offered to the Tritanrix™-HepB/Hiberix™ control group to protect them against meningococcal diseases of these serogroups: Mencevax™ ACWY will be given when the child is two years of age, or, if a meningitis epidemic occurs, earlier in the second year of life.

- Treatment allocation: randomized (1:1). Refer to Section 6.3 for a detailed description of the randomization method.

- Blinding: Double-blind. Refer to Section 6.4 for details of blinding procedure.

- Study duration per subject: approximately ***22.5 months to include the retrospective follow-up of SAE(s) up to the time that subjects are at least 24 months of age.***

- Data collection: Hard copy Case Report Form (CRF)

- For all subjects: 4 blood samples (3.5 ml each):
  - immediately before the first dose of the primary vaccination course
  - one month after the third dose of the primary vaccination course
  - just prior to administration of the plain polysaccharides of serogroups A and C
  - one month after the administration of the plain polysaccharides of serogroups A and C.**:**

- 4-day follow-up of solicited local and general symptoms after each dose.

- One month follow-up for unsolicited symptoms after each dose.

- Reporting of serious adverse events during the entire study period.

- ***Retrospective follow-up of SAE(s). The parents/guardians of study subjects will be contacted by the local health staff when their child is at least 24 months of age for a follow-up of SAE(s) (i.e. any SAE(s) that occurred since the last study visit [Visit 7] up to the time that subjects are at least 24 months of age).***

(Amendment 7: 12 April 2006)

4 Study Cohort

4.1 Number of subjects

Target enrolment will be 280 healthy male and female infants (140 subjects per group) to provide 220 evaluable subjects (110 per group) for the immunogenicity analysis. The study will be conducted at the Navrongo Health Research Center in Ghana (War Memorial Hospital and several health centres of Navrongo health district).

Enrolment will be terminated when 280 subjects have been enrolled.

Refer to Section 10.3 for a detailed description of the criteria used in the estimation of sample size.

Details of recruitment at each health center (immunization point), including any criteria for termination of enrolment, will be discussed in the recruitment plan, which is summarized in Appendix C of this document.

If at the time of the initiation of the booster phase any parent/guardian declines participation of his/her child, refusal will be documented as instructed in the "subject tracking document" provided by GSK Biologicals. A copy of the completed tracking document will be forwarded to GSK Biologicals' Study Monitor. The information will be entered in the GSK Biologicals' clinical database for use in identification of any safety issue that may have prevented a subject's participation.

4.2 Inclusion criteria

All subjects must satisfy the following criteria at study entry:

- Subjects for whom the investigator believes that their parents/guardians can and will comply with the requirements of the protocol (e.g., completion of the diary cards, return for follow-up visits) should be enrolled in the study.

- A male or female between, and including, 6 and 8 weeks of age at the time of the first vaccination.
- Written informed consent obtained from the parent or guardian of the subject.
- Free of obvious health problems as established by medical history and clinical examination before entering into the study.

4.3 Exclusion criteria for enrolment

The following criteria should be checked at the time of study entry. If any apply, the subject must not be included in the study:

- Use of any investigational or non-registered drug or vaccine other than the study vaccine(s) within 30 days preceding the first dose of study vaccine, or planned use during the study period.
- Chronic administration (defined as more than 14 days) of immunosuppressants or other immune-modifying drugs since birth. (For corticosteroids, this will mean prednisone, or equivalent, ≥ 0.5 mg/kg/day. Inhaled and topical steroids are allowed.)
- Planned administration/ administration of a vaccine not foreseen by the study protocol within 30 days before each dose of vaccine, with the exception of OPV.
- Hepatitis B and BCG vaccine given within two weeks prior to vaccination.
- Previous vaccination against diphtheria, tetanus, pertussis, *Haemophilus influenzae* type b or meningococcal serogroup A or C diseases.
- History of diphtheria, tetanus, pertussis, hepatitis B, *Haemophilus influenzae* type b or meningococcal serogroup A or C diseases.
- Known exposure to (direct contact) diphtheria, tetanus, pertussis, hepatitis B, *Haemophilus influenzae* type b and/or meningococcal disease since birth.
- A diagnosis or clinical suspicion of an immune suppressive or immunodeficient condition of any cause based on full clinical history and medical examination.
- A family history of congenital or hereditary immunodeficiency.
- History of allergic disease or reactions likely to be exacerbated by any component of the vaccine.
- Major congenital defects or serious chronic illness.
- Babies for which birth weight is <2 kg (if known), and/or malnutrition at visit 1.
- History of any neurologic disorders or seizures.
- Acute disease at the time of enrolment. (Acute disease is defined as the presence of a moderate or severe illness with or without fever. All vaccines can be administered to persons with a minor illness such as diarrhea, mild upper respiratory infection with or without low-grade febrile illness, i.e., axillary temperature <37.5°C or rectal temperature <38.0°C).

Note: Subjects with fever at the time of first vaccination (i.e., axillary temperature ≥ 37.5°C or rectal temperature ≥ 38.0°C) need not necessarily be excluded but can be vaccinated at a later date after the subject recovers and if the subject still meets all eligibility criteria.

- Administration of immunoglobulins and/or any blood products since birth or planned administration during the study period.
- Other conditions which in the opinion of the investigator may potentially interfere with interpretation of study outcomes.

4.4 Elimination criteria during the study

The following criteria should be checked at each visit subsequent to the first visit. If any become applicable during the study, it will not require withdrawal of the subject from the study but does determine a subject's evaluability in the according-to-protocol (ATP) analysis. See Section 10.4 for definition of study cohorts to be evaluated.

- Use of any investigational or non-registered drug or vaccine other than the study vaccine(s) during the study period.
- Chronic administration (defined as more than 14 days) of immunosuppressants or other immune-modifying drugs during the study period. (For corticosteroids, this will mean prednisone, or equivalent, ≥ 0.5 mg/kg/day. Inhaled and topical steroids are allowed).
- Administration of a vaccine not foreseen by the study protocol during the period starting from **30 days** before the first dose of vaccine(s) and ending 30 days after the last dose of vaccine(s) with the exception of OPV.
- Administration of a meningococcal vaccine not foreseen by the study protocol during the entire study period.
- Administration of immunoglobulins and/or any blood products during the study period.

4.5 Contraindications to subsequent vaccination

The following adverse events (AEs) constitute absolute contraindications to further administration of the **study vaccines**; if any of these AEs occur during the study, the subject must not receive additional doses of vaccine but may continue other study procedures at the discretion of the investigator (see Section 9). The subject must be followed until resolution of the event, as with any AE (see Section 8.7):

- Anaphylactic reaction following the administration of vaccine(s).

- Known hypersensitivity to any component of the vaccine, or subjects having shown signs of hypersensitivity after previous administration of diphtheria, tetanus, pertussis, HB, Hib or meningococcal vaccines.
- Any confirmed or suspected immunosuppressive or immunodeficient condition, including human immunodeficiency virus (HIV) infection.

The following AEs constitute contraindications to administration of the study vaccines at that point in time; if any one of these AEs occurs at the time scheduled for vaccination, the subject may be vaccinated at a later date, within the time window specified in the protocol (see Section 5.3) or withdrawn at the discretion of the investigator (see Section 9). The subject must be followed until resolution of the event, as with any adverse event (see Section 8.7):

- Acute disease at the time of vaccination. (Acute disease is defined as the presence of a moderate or severe illness with or without fever. All vaccines can be administered to persons with a minor illness such as diarrhoea, mild upper respiratory infection with or without low-grade febrile illness, i.e., axillary or oral temperature $< 37.5°C$/rectal temperature $< 38°C$).
- Axillary or oral temperature $\geq 37.5°C$/rectal temperature $\geq 38°C$ at the time of vaccination.

DTPw-HBV (Tritanrix™-HepB)

The following AEs associated with DTP vaccination constitute absolute contraindications to further administration of DTP. If any of these AEs occur during the study, the subject must be withdrawn and must be followed until resolution of the event, as with any adverse event (see Section 8.7).

- Encephalopathy (not due to another identifiable cause). This is defined as an acute, severe central nervous system disorder occurring within 7 days following vaccination, and generally consisting of major alterations in consciousness, unresponsiveness, generalized or focal seizures that persist more than a few hours, with failure to recover within 24 hours. Even though causation by DTP vaccine cannot be established, no subsequent doses of pertussis vaccine should be given. In these circumstances the vaccination course should be continued with DT and HB vaccines.

Special warnings and special precautions for use

If any of the following events occur in temporal relation to receipt of Tritanrix™- HepB, the decision to give subsequent doses of vaccine containing the pertussis component should be carefully considered.

- Axillary or oral temperature of ≥ 40.0°C/rectal temperature ≥ 40.5°C within 48 hours of vaccination, not due to another identifiable cause.
- Collapse or shock-like state (hypotonic-hyporesponsive episode) within 48 hours of vaccination.
- Persistent crying lasting ≥ 3 hours, occurring within 48 hours of vaccination.
- Convulsions with or without fever, occurring within 3 days of vaccination.

There may be circumstances, such as a high incidence of pertussis, when the potential benefits outweigh possible risks.

A history of febrile convulsions, a family history of convulsions, a family history of Sudden Infant Death Syndrome (SIDS) and a family history of an adverse event following Tritanrix™-HepB vaccination do not constitute contraindications.

As with all injectable vaccines, appropriate medical treatment should always be readily available in case of anaphylactic reactions following the administration of the vaccine. For this reason, the vaccinee should remain under medical supervision for 30 minutes after vaccination.

Tritanrix™-HepB should be administered with caution to subjects with thrombocytopenia or a bleeding disorder since bleeding may occur following an intramuscular administration to these subjects.

Tritanrix™-HepB should under no circumstances be administered intravenously.

Hiberix™

The **Hiberix™** vaccine should not be administered to subjects with known hypersensitivity to any component of the vaccine, or to subjects having shown signs of hypersensitivity after previous administration of Hib vaccines.

5 Conduct of Study

5.1 Ethics and regulatory considerations

The study will be conducted according to Good Clinical Practice (GCP), the 1996 version of the Declaration of Helsinki (Protocol Appendix A), and local rules and regulations of the country. The study protocol will be submitted for ethical review to the Navrongo Health Center Ethics Committee, WHO Ethical Review Board, the Ethics Committee of the London School of Hygiene and Tropical Medicine**.**

5.1.1 Institutional Review Board/Independent Ethics Committee (IRB/IEC)

The IRB/IEC must be constituted according to the local laws/customs of each participating country. The ICH Harmonized Tripartite Guideline for Good Clinical Practice recommends that the IRB/IEC should include:
(a) At least five members.
(b) At least one member whose primary area of interest is in a non-scientific area.
(c) At least one member who is independent of the institution/ study site.
Only those IRB/IEC members who are independent of the investigator and the sponsor of the study should vote/ provide opinion on a study-related matter.

A list of IRB/IEC members and their qualifications should be obtained by the investigator.

This protocol and any other documents that the IRB/IEC may need to fulfil its responsibilities, including subject recruitment procedures and information about payments and compensation available to subjects, will be submitted to the IRB/IEC by the investigator. Written unconditional approval of the IRB/IEC must be in the possession of the investigator and GSK Biologicals before commencement of the study. Relevant GSK Biologicals' data will be supplied by the investigator to the hospital/ university/ independent IRB/IEC for review and approval of the protocol. Verification of IRB/IEC unconditional approval of the protocol and the written informed consent statement will be transmitted by the investigator to the Site Monitor using the standard notification form, prior to shipment of vaccine supplies and CRFs to the site. This approval must refer to the study by exact protocol title and number, and should identify the documents reviewed and state the date of review.

No deviations from, or changes to, the protocol should be initiated without prior written IRB/IEC approval/ favourable opinion of an appropriate amendment, except when necessary to eliminate immediate hazards to the subjects or when the change(s) involves only logistical or administrative aspects of the study (e.g., change of monitor[s], telephone number[s].) Modifications are submitted to the IRB/IEC for information only. However, written verification that the modification was submitted should be obtained. Approvals/ verifications must be transmitted in writing to the Site Monitor, by the investigator.

The IRB/IEC must be informed by the investigator of:

- all subsequent protocol amendments, informed consent changes or revisions of other documents originally submitted for review,
- serious and/or unexpected adverse events occurring during the study, where required,
- all subsequent protocol modifications (for information),

- new information that may affect adversely the safety of the subjects or the conduct of the study,
- an annual update and/or request for re-approval, where required,
- when the study has been completed, where required.

5.1.2 Informed consent

In obtaining and documenting informed consent, the investigator should comply with the applicable regulatory requirement(s), and should adhere to GCP and to the ethical principles that have their origin in the 1996 version of the Declaration of Helsinki. Prior to the beginning of the trial, the investigator should have the IRB/IEC’s written approval/favourable opinion of the written informed consent form and any other written information to be provided to the subjects’ parents/guardians.

Information should be given in both oral and written form whenever possible and as deemed appropriate by the IRB/IEC.

An investigator or designate will describe the protocol to potential subjects’ parents/guardians face to face. The Subject Information and Consent Form may be read to the subjects’ parents/guardians, but, in any event, the investigator or designate shall give the subjects’ parents/guardians ample opportunity to inquire about details of the study and ask any questions before dating and signing the Consent Form.

Subject information and consent forms must be in a language fully comprehensible to the prospective subjects’ parents/guardians. Informed consent shall be documented by the use of a written consent form approved by the IRB/IEC and signed/thumbprinted and dated by the parent/guardian, and by the person who conducted the informed consent discussion. The signature/thumbprint confirms the consent is based on information that has been understood. All illiterate individuals will have the study, the Subject Information and Consent Form explained to them point by point by the interviewer in the presence of an impartial witness. The witness will personally sign and date the consent form. Oral witnessed consent will replace written consent only in countries where the local custom is contrary or if the parents’/guardians’ illiteracy precludes this and provided that the local legal obligations are fulfilled.

Each subject's signed/ thumb printed informed consent form must be kept on file by the investigator for possible inspection by Regulatory Authorities and/or GSK Biologicals’ professional and Regulatory Compliance persons. The parents/guardians should receive a copy of the signed/ thumb printed and dated written informed consent form and any other written information provided to the subjects’ parents/guardians, and should receive copies

of any signed and dated consent form updates. Any amendments to the written information will be provided to subjects' parents/guardians.

Both the informed consent discussion and the written informed consent form and any other written information to be provided to the subjects' parents/guardians should include explanations of the following:

(a) That the trial involves research.
(b) The purpose of the trial.
(c) The trial treatment(s) and the probability for random assignment to each treatment.
(d) The trial procedures to be followed, including all invasive procedures.
(e) The subject's parents'/guardians' responsibilities.
(f) Those aspects of the trial that are experimental.
(g) The reasonably foreseeable risks or inconveniences to the subjects and, when applicable, to an embryo, fetus or nursing infant.
(h) The reasonable expected benefits. When there is no intended clinical benefit to subjects, the subjects and/or subjects' parents/guardians should be made aware of this.
(i) The alternative procedure(s) or course(s) of treatment/ methods of prevention that may be available to subjects, and their important potential benefits and risks.
(j) The compensation and/or treatment available to subjects in the event of trial-related injury.
(k) The anticipated prorated payment, if any, to subjects' parents/guardians for participating in the trial.
(l) The anticipated expenses, if any, to subjects' parents/guardians for participating in the trial.
(m) That the subjects' participation in the trial is voluntary and subjects' parents/guardians may refuse to participate or withdraw from the trial, at any time, without penalty or loss of benefits to which subjects are otherwise entitled.
(n) That the monitor(s), the auditor(s), the IRB/IEC, and the regulatory authority(ies) will be granted direct access to the subject's original medical records for verification of clinical trial procedures and/or data, without violating the confidentiality of subjects, to the extent permitted by the applicable laws and regulations and that, by signing a written informed consent, the subject's parents/guardians is authorizing such access.
(o) That records identifying subjects will be kept confidential and, to the extent permitted by the applicable laws and/or regulations, will not be made publicly available. If the results of the trial are published, subjects' identity will remain confidential.
(p) That the subjects' parents/guardians will be informed in a timely manner if information becomes available that may be relevant to the subjects' parents/guardians willingness for their continued participation in the trial.
(q) The person(s) to contact for further information regarding the trial and the rights of trial subjects, and who to contact in the event of trial-related injury.

(r) The foreseeable circumstances and/or reasons under which a subject's participation in the trial may be terminated.
(s) The expected duration of a subject's participation in the trial.
(t) The approximate number of subjects involved in the trial.

GSK Biologicals will prepare a representative subject information sheet/informed consent document which will embody all the elements described above. While it is strongly recommended that this representative document be followed as closely as possible, the informed consent requirements given in this document are not intended to pre-empt any local regulations which require additional information to be disclosed for informed consent to be legally effective. Clinical judgement, local regulations and requirements should guide the final structure and content of the document.

The investigator has the final responsibility for the final presentation of the subject information sheet/informed consent document, respecting the mandatory requirements of local regulations. The consent form generated by the investigator with the assistance of the local sponsor's representative, must be approved (along with the protocol, and any other necessary documentation) by the IRB/IEC and be acceptable to GSK Biologicals.

5.1.3 Independent Data Monitoring Committee (IDMC)

Composition of the IDMC

An independent committee consisting of up to five experts in meningococcal diseases, paediatrics, vaccines, statistics and other appropriate disciplines will be appointed to oversee ethical and safety aspects of the study conduct.

The Charter (terms of references) of the IDMC will be written after the first meeting of the IDMC members, prior to study start.

Role of the IDMC

The role of the IDMC includes the review of the implementation and progress of the study. It provides initial, regular, and closing advice on safety-related issues to GSK Biologicals. Its advice is based on the interpretation of study data with reference to the study protocol.

The IDMC will confer before the initiation of the study (pre-initiation review), during the study proper and at the close out of the study to review any relevant safety data; after study end, the IDMC will have a first assessment report. Other unscheduled meetings may be required. Meetings must be documented and minutes made available for the study files

on site and to the sponsors. The IDMC may, if deemed necessary, convene a meeting with or request further information from the Principal Investigators, the Local Safety Monitor, GSK Biologicals, WHO and designated project representatives at any stage of the study.

The IDMC must be informed by the investigator of:

- all subsequent protocol amendments, informed consent changes or revisions of other documents originally submitted for review
- systemic grade 3 adverse events (AEs) related to vaccination**,** serious adverse events and adverse events suggesting a lack of efficacy of the vaccine occurring during the study (transmitted through GSK Biologicals for SAEs and through the Local safety Monitor for the other adverse events)
- all subsequent protocol modifications (for information)
- new information that may affect adversely the safety of the subjects or the conduct of the study.

The IDMC will be empowered to put the study on hold pending review of potential safety issues. All SAEs, including death, will be reported by the Principal Investigator to the local safety monitor, to the Manager of Clinical Safety Vaccines at GSK Biologicals, to WHO and to the WHO safety monitor. GSK Biologicals will be responsible to provide all information related to the SAEs to the IDMC according to the IDMC Charter.

Reporting to IDMC **– Role of Local Safety Monitor**

A Local Safety Monitor (LSM) will act independently from the investigational team. The overall role of the Local Safety Monitor (LSM), who will be an experienced clinician based in-country, will be to support the clinical investigators and to act as a link between the investigators and the IDMC. All systemic grade 3 AEs probably or suspected to be related to vaccination, all SAEs and all AEs suggesting a lack of efficacy of the vaccine will be reported to him/her. His/her involvement will be particularly important when decisions have to be made quickly. In exceptional circumstances, for example a death possibly related to vaccination, he/she will have the authority to suspend the whole or any specific aspect of the trial pending discussion with the IDMC.

The LSM’s role will include:

- acting as the study volunteer's advocate
- provide the IDMC and the medical monitor of GSK Biologicals with a blinded listing of non-serious systemic grade 3 symptoms (solicited and unsolicited) that are related with vaccination, and of non-serious adverse events suggesting a lack of efficacy of the vaccine, this according to the IDMC Charter (note: GSK Biologicals will send all

information related to serious adverse events to the IDMC according to the IDMC Charter).

- reviewing blinded reactogenicity data according to IDMC charter and discussing with IDMC if necessary
- promptly communicating relevant safety information to the IDMC
- providing advice to the investigators on whether a set of clinical circumstances in a study warrants formal notification to the IDMC
- providing clinical advice on any illness in study subjects especially in circumstances in which treatment might influence the course of the trial
- Unblinding a subject if deemed necessary**,** upon request of the investigator/physician in charge of the subject, to allow for adequate treatment.

The LSM will liaise closely with the chair of the IDMC throughout the course of the trial.

5.2 General study aspects

The maximum distance between a subject's compound and the nearest health facility will be 10 km. Each visit to the clinic for vaccination will last approximately 4 hours; each home visit by a field worker will last about 15 minutes.

The dates of administration of a dose of hepatitis B vaccine and/or Bacille Calmette-Guérin (BCG) and/or Oral Polio Vaccine (OPV) vaccine as well as any other vaccines other than the study vaccines that may be given to the subjects from birth until the end of the study will be documented in the concomitant vaccination section of the individual CRF.

Any meningococcal vaccine given to the **mother** of the child from one year before the first study vaccine dose administration up to the first study vaccine administration will be documented in the concomitant vaccination section of the individual CRF (see section 6.8 for details).

One specific ancillary study will be done on the subjects participating in this study by Abraham Hodgson, Navrongo Health Research Center (NHRC), Navrongo, Ghana; Gerd Pluschke, Molecular Immunology, Swiss Tropical Institute (STI), Basel, Switzerland. GSK Biologicals is not sponsor of this ancillary study but the protocol of this ancillary study is attached as an Appendix to this protocol (refer Appendix H for further details) for operational reasons (only one subject information sheet will be written and one informed consent will be submitted to the approval of the parents/guardians of the child, however, any parent/guardian accompanying the child at visits subsequent to the first visit who has not already signed an Informed Consent for this study will also have to sign an

Informed Consent prior to providing any throat swab). This ancillary study will be reported separately by the investigators of the ancillary study. This ancillary study requires collection of throat swabs from the parent/guardian and infant prior to the first dose (6-8 weeks) and at 10 weeks, 18 weeks, 9 months and 12 months of age. The collection of throat swab will be detailed in the study procedures (section 5. 3) and the subject information sheet.

5.3 Outline of study procedures

Table 5-1: List of study procedures

	Primary Phase (759346/009)				***Booster Phase (104430)***		
Age	±6 w	±10 w	±14 w	±18 w	± 9 m	±12 m	±13 m
Visit	**VISIT 1**	**VISIT 2**	**VISIT 3**	**VISIT 4**	**VISIT 5**	**VISIT 6**	**VISIT 7**
Timing	Month 0	Month 1	Month 2	Month 3	Month 7.5	Month 10.5	Month 11.5
Sampling time point	Pre vacc			Post vacc III		Pre booster	Post booster
Informed consent#	•	(•)#		(•)#	(•)#	(•)#	
Check inclusion criteria	•						
Check exclusion criteria	•						
Check elimination criteria		•	•	•	•	•	•
Check contraindications		•	•		○##	•	
Medical history	•						
Physical examination	•	•	•	•	•	•	•
Pre-vaccination body temperature	•	•	•		•	•	
Measure/record height and weight	•						
Randomization	•						
Blood sampling for antibody determination (3.5 ml)	•*			•		•	•
Throat swab	•**	•**		•**	•**	•**	
Vaccination	•	•	•		•***	•****	
Daily post-vaccination recording of solicited symptoms (days 0-3) by field workers	•	•	•			•	
Recording of non serious adverse events occurring one month (minimum 30 days) post-vaccination, by investigator	•	•	•	•		•	•
Return of diary cards		•	•	•			•
Diary card transcription		•	•	•			•
Record any concomitant medication	•	•	•	•	•	•	•
Retrospective recording of relevant concomitant medication/vaccination since Visit 4###					•		
Reporting of Serious Adverse Events	•	•	•	•	•	•	•
Study Conclusion of Prirmary Phase				•			
Study Conclusion of Booster Phase							•

Footnotes to Table 5-1
• denotes a procedure which must be recorded in the individual CRF.<br>° denotes a procedure which need not be recorded in the individual CRF.<br># any parent/guardian accompanying the child at visits subsequent to the first visit who has not already signed an Informed Consent for this study will have to sign an Informed Consent prior to providing any throat swab<br>.## Contraindications for measles and yellow fever vaccines.<br>### Relevant medication/treatment will include meningococcal vaccine, any investigational or non-registered product (drug or vaccine), any immunosuppressant(s) or other immune-modifying drug(s), immunoglobulins and/or any blood product(s).<br>* a minimum of 2 ml may be taken at prevaccination timepoint. See Section 5.5.2; ** from the subject and subject's parents/guardians; ***Measles, yellow fever vaccine<br>****1/5th of dose of Mencevax™ AC<br>Note: BCG and OPV vaccines are given at birth (or at least 2 weeks before the subject's first visit) according to the nationally recommended immunization schedule.<br>OPV vaccine can be administered to the subjects at 6, 10 and 14 weeks of age concomitantly with the study vaccines or at any time during the study, whichever is more appropriate.

Table 5-2: List of study procedures for the extended safety follow-up phase

	EXTENDED SAFETY FOLLOW-UP PHASE
Age	***±24 m***
Visit	
Timing	***Month 22.5***
Informed consent	•
Retrospective recording of SAE(s) since Visit 7 up to the time that subjects are aged at least 24 months	•
Study conclusion	•

(Amendment 7: 12 April 2006)

It is the investigator's responsibility to ensure that the intervals between visits/contacts are strictly followed. These intervals determine each subject's evaluability in the according-to-protocol analyses (see Sections 4.4 and 10.4 for details of criteria for evaluability and cohorts to be analyzed).

Table 5-3: Intervals between study visits

Interval	Size of interval
1 (Visit 1→Visit 2)*	28–42 days
2 (Visit 2→Visit 3)*	28–42 days
3 (Visit 3→Visit 4)*	30–42 days
4 (Visit 5)	Approx 9 months of age (range 8-10 months)
5 (Visit 6)	Approx 12 months of age (range 11-13 months)
6 (Visit 6→Visit 7)*	30–42 days

* The date of the previous visit serves as reference date.

5.4 Detailed description of study stages/visits

A description of each stage of the study, as summarized in the schedule of assessments (see Table 5-1) is given hereafter.

**Visit 1: 6 weeks of age: Study Day 0: Vaccination**

- Written informed consent from the subject's parent/guardian.
- Check of inclusion criteria for enrolment.
- Check of exclusion criteria for enrolment.
- Medical history taking.
- Physical examination by the investigator including measurement and recording of pre-vaccination temperature.
- Measurement and recording of height and weight.
- Recording of any concomitant medication administered (See Section 6.8). This includes also the recording of any meningococcal vaccine given to the mother during the year preceding the visit 1.
- Randomization (See Section 6.3).
- Provision of temporary study identification card.
- Collection of blood for serology: it is understood that collection of blood in infants aged 6 weeks may be technically difficult. Therefore a minimum of 2.0 ml of whole blood, which is the minimum volume of blood that will allow the essential serology to be undertaken, has to be taken according to instructions in Appendix D. However, whenever possible a 3.5 ml sample should be collected to provide the 1.5 ml sample of serum that is needed for full serological assessment of the response to the vaccine and for validation assays. See Section 5.5.2.
  Technically skilled and trained individuals will draw blood to avoid unnecessary pain and technical failure as much as possible.
  When materials are provided by GSK Biologicals, it is **MANDATORY** that all clinical samples (including serum samples) will be collected and stored using exclusively those materials in the appropriate manner. The use of other materials could result in the exclusion of the subject from the ATP analysis (See Section 10.4 for definition of study cohorts to be evaluated). The investigator must ensure that his/her personnel and the laboratory(ies) under his/her supervision comply with this requirement. However, when GSK Biologicals does not provide material for collecting and storing clinical samples, then appropriate materials from the investigator's site are to be used. Refer to Appendix D and Appendix E.
- Collection of throat swabs from the subject and subject's parent/guardian
- Vaccination: intramuscular administration of one dose of Tritanrix™-

HepB/Hib-MenAC or Tritanrix™-HepB/Hiberix™ according to the guidelines set out in Section 6.1.6.
The vaccinees will be observed closely for at least 30 minutes, with appropriate medical treatment readily available in case of a rare anaphylactic reaction following the administration of vaccines.

- The field worker will be given a diary card and will visit the subjects at their home to record, on the day of vaccine injection (day 0) and during the 3 subsequent days (days 1-3) or up to resolution of the symptoms, whichever is longer, the subject's body temperature and any local (at the injection site) or general adverse events (see section 8.5.1).
- The field worker will visit the subjects' parents/guardians one month after each vaccination to remind them to go for the next visit.
- The subjects' parents/guardians will be instructed to contact the investigator/study contact person at the health centre immediately should the subject manifest any signs or symptoms they perceive as serious.

Visit 2: 10 weeks of age: Study Month 1 (28-42 days after Visit 1): Vaccination

- Provision of definitive study ID card (plastified ID card including picture of parent/guardian and infant).
- The investigator will verify the diary cards provided by the field workers. He will transcribe the information into the appropriate sections of the case report form (CRF), in English.
- Check of elimination criteria (see Section 4.4).
- Check of any contraindications (see Section 4.5).
- Recording of any concomitant medication administered since the last visit (see Section 6.8).
- Recording of any serious and any unsolicited adverse events which might have occurred since the last visit.
- Physical examination by the investigator including measurement and recording of pre-vaccination temperature.
- Collection of throat swabs from the subject and subject's parent/guardian (any parent/guardian accompanying the child at visits subsequent to the first visit who has not already signed an Informed Consent for this study will have to sign an Informed Consent prior to providing any throat swab).
- Vaccination: intramuscular administration of one dose of Tritanrix™-HepB/Hib-MenAC or Tritanrix™-HepB/Hiberix™ according to the guidelines set out in Section 6.1.6.
  The vaccinees will be observed closely for at least 30 minutes, with appropriate medical treatment readily available in case of a rare

anaphylactic reaction following the administration of vaccines.

- The field worker will be given a diary card and will visit the subjects at their home to record, on the day of vaccine injection (day 0) and during the 3 subsequent days (days 1-3) or up to resolution of the symptoms, whichever is longer, the subject's body temperature and any local (at the injection site) or general adverse events (see section 8.5.1).
- The field worker will visit the subjects' parents/guardians one month after each vaccination to remind them to go for the next visit.
- The subjects' parents/guardians will be instructed to contact the investigator immediately should the subject manifest any signs or symptoms they perceive as serious.

N.B. The parent/guardian of the study subject will be asked to present the study ID card <u>every time</u> they attend the hospital / health centers.

Note: Any parent/guardian accompanying the child at visits subsequent to the first visit who has not already signed an Informed Consent for this study will have to sign an Informed Consent prior to providing a throat swab.

Visit 3: 14 weeks of age: Study Month 2 (28-42 days after Visit 2): Vaccination

- The investigator will verify the diary cards provided by the field workers. He will transcribe the information into the appropriate sections of the CRF, in English.
- Check of elimination criteria (see Section 4.4).
- Check of any contraindications (see Section 4.5).
- Recording of any concomitant medication administered since the last visit (see Section 6.8).
- Recording of any serious and any unsolicited adverse events which might have occurred since the last visit.
- Physical examination by the investigator including measurement and recording of pre-vaccination temperature.
- Vaccination: intramuscular administration of one dose of Tritanrix™-HepB/Hib-MenAC or Tritanrix™-HepB/Hiberix™ according to the guidelines set out in Section 6.1.6.
  The vaccinees will be observed closely for at least 30 minutes, with appropriate medical treatment readily available in case of a rare anaphylactic reaction following the administration of vaccines.
- The field worker will be given a diary card and will visit the subjects at their home to record, on the day of vaccine injection (day 0) and during the 3 subsequent days (days 1-3) or up to resolution of the symptoms, whichever is

longer, the subject's body temperature and any local (at the injection site) or general adverse events (see section 8.5.1).

- The field worker will visit the subjects' parents/guardians one month after each vaccination to remind them to go for the next visit.
- The subjects' parents/guardians will be instructed to contact the investigator/study contact person at the health centre immediately should the subject manifest any signs or symptoms they perceive as serious.

Visit 4: 18 weeks of age: Study Month 3 (30-42 days after Visit 3): Follow-up visit

- The investigator will verify the diary cards provided by the field workers. He will transcribe the information into the appropriate sections of the CRF, in English.
- Recording of any unsolicited adverse events which might have occurred within one month (minimum 30 days) following the previous vaccination (see Section 8.7).
- Recording of any serious adverse events which might have occurred since the last visit.
- Check of elimination criteria (see Section 4.4).
- Recording of any concomitant medication administered since the last visit (see Section 6.8).
- Physical examination by the investigator.
- Collection of throat swabs from the subject and subject's parent/guardian (any parent/guardian accompanying the child at visits subsequent to the first visit who has not already signed an Informed Consent for this study will have to sign an Informed Consent prior to providing any throat swab).
- Collection of blood for serology: a minimum of 3.5 ml of whole blood to provide a minimum of 1.5 ml of serum according to instructions in Appendix D.
  Technically skilled and trained individuals will draw blood to avoid unnecessary pain and technical failure as much as possible.
  When materials are provided by GSK Biologicals, it is **MANDATORY** that all clinical samples (including serum samples) will be collected and stored using exclusively those materials in the appropriate manner. The use of other materials could result in the exclusion of the subject from the ATP analysis (See Section 10.4 for definition of study cohorts to be evaluated). The investigator must ensure that his/her personnel and the laboratory(ies) under his/her supervision comply with this requirement. However, when GSK Biologicals does not provide material for collecting and storing clinical samples, then appropriate materials from the investigator's site are to be used.

Refer to Appendix D and Appendix E.

- The subjects' parents/guardians will be instructed to contact the investigator/study contact person at the health centre immediately should the subject manifest any signs or symptoms they perceive as serious.

Note: Any parent/guardian accompanying the child at visits subsequent to the first visit who has not already signed an Informed Consent for this study will have to sign an Informed Consent prior to providing a throat swab

- Study conclusion of the primary phase

Visit 5: 9 months of age (8-10 months) -Vaccination with Measles and Yellow Fever vaccine

- If at the time of the initiation of the booster phase any parent/guardian declines participation of his/her child, refusal will be documented as instructed in the "subject tracking document" provided by GSK Biologicals. A copy of the completed tracking document will be forwarded to GSK Biologicals' Study Monitor. The information will be entered in the GSK Biologicals' clinical database for use in identification of any safety issue that may have prevented a subject's participation.
- Recording of any serious adverse events which might have occurred since the last visit.
- Check of elimination criteria (see Section 4.4).
- Recording of concomitant medication administered since the last visit (see Section 6.8).
- Physical examination by the investigator including measurement and recording of pre-vaccination temperature.
- Check of contraindications for measles and yellow fever vaccine (this will not be documented in the individual CRF)
- Retrospective recording of any relevant concomitant medication/ vaccination since the last visit according to instructions provided in Section 6.8. Relevant vaccines will include meningococcal vaccines and investigational or non registered vaccines and will be recorded with trade name, route of administration and date(s) of administration. Relevant medications/treatments will include any investigational or non-registered product(s), any immunosuppressant(s) or other immun-modifying drug(s), immunoglobulins and/or any blood products. All such medication/treatment is to be recorded with generic name of the medication (trade names are allowed for combination drugs, i.e. multi-component drugs), medical indication, total daily dose, route of administration, start and end dates of treatment.
- Collection of throat swabs from the subject and subject's parent/guardian (any

parent/guardian accompanying the child at visits subsequent to the first visit who has not already signed an Informed Consent for this study will have to sign an Informed Consent prior to providing any throat swab).

- Vaccination: intramuscular/subcutaneous administration of one dose of measles vaccine and intramuscular administration of one dose of yellow fever vaccine.
  The vaccinees will be observed closely for at least 30 minutes, with appropriate medical treatment readily available in case of a rare anaphylactic reaction following the administration of vaccines.
- The subjects' parents/guardians will be instructed to contact the investigator/study contact person at the health centre immediately should the subject manifest any signs or symptoms they perceive as serious.

Note: Any parent/guardian accompanying the child at visits subsequent to the first visit who has not already signed an Informed Consent for this study will have to sign an Informed Consent prior to providing a throat swab

Visit 6: 12 months of age (11-13 months) - Booster vaccination

- Recording of any serious adverse events which might have occurred since the last visit.
- Check of elimination criteria (see Section 4.4).
- Recording of any concomitant medication administered since the last visit (see Section 6.8).
- Physical examination by the investigator including measurement and recording of pre-vaccination temperature.
- Collection of throat swabs from the subject and subject's parent/guardian (any parent/guardian accompanying the child at visits subsequent to the first visit who has not already signed an Informed Consent for this study will have to sign an Informed Consent prior to providing any throat swab)**.**
- Collection of blood for serology: a minimum of 3.5 ml of whole blood to provide a minimum of 1.5 ml of serum according to instructions in Appendix D.
  Technically skilled and trained individuals will draw blood to avoid unnecessary pain and technical failure as much as possible.
  When materials are provided by GSK Biologicals, it is **MANDATORY** that all clinical samples (including serum samples) will be collected and stored using exclusively those materials in the appropriate manner. The use of other materials could result in the exclusion of the subject from the ATP analysis (See Section 10.4 for definition of study cohorts to be evaluated). The investigator must ensure that his/her personnel and the laboratory(ies) under

his/her supervision comply with this requirement. However, when GSK Biologicals does not provide material for collecting and storing clinical samples, then appropriate materials from the investigator's site are to be used. Refer to Appendix D and Appendix E.

- Check of any contraindications (see Section 4.5).
- Vaccination: intramuscular administration of 1/5th of a dose of Mencevax™ AC according to the guidelines set out in Section 6.1.6.
  The vaccinees will be observed closely for at least 30 minutes, with appropriate medical treatment readily available in case of a rare anaphylactic reaction following the administration of vaccines.
- The field worker will be given a diary card and will visit the subjects at their home to record, on the day of vaccine injection (day 0) and during the 3 subsequent days (days 1-3) or up to resolution of the symptoms, whichever is longer, the subject's body temperature and any local (at the injection site) or general adverse events (see section 8.5.1).
- The field worker will visit the subjects' parents/guardians one month after each vaccination to remind them to go for the next visit.
- The subjects' parents/guardians will be instructed to contact the investigator/study contact person at the health centre immediately should the subject manifest any signs or symptoms they perceive as serious.

Note: Any parent/guardian accompanying the child at visits subsequent to the first visit who has not already signed an Informed Consent for this study will have to sign an Informed Consent prior to providing a throat swab.

Visit 7: Study Month 11.5 (30-42 days after Visit 6): Blood Sampling and Follow-up visit

- The investigator will verify the diary cards provided by the field workers. He will transcribe the information into the appropriate sections of the CRF, in English.
- Recording of any serious adverse events which might have occurred since the last visit.
- Check of elimination criteria (see Section 4.4).
- Recording of any concomitant medication administered since the last visit (see Section 6.8).
- Physical examination by the investigator.
- Collection of blood for serology: a minimum of 3.5 ml of whole blood to provide a minimum of 1.5 ml of serum according to instructions in Appendix D.
  Technically skilled and trained individuals will draw blood to avoid

unnecessary pain and technical failure as much as possible.
When materials are provided by GSK Biologicals, it is **MANDATORY** that all clinical samples (including serum samples) will be collected and stored using exclusively those materials in the appropriate manner. The use of other materials could result in the exclusion of the subject from the ATP analysis (See Section 10.4 for definition of study cohorts to be evaluated). The investigator must ensure that his/her personnel and the laboratory(ies) under his/her supervision comply with this requirement. However, when GSK Biologicals does not provide material for collecting and storing clinical samples, then appropriate materials from the investigator's site are to be used. Refer to Appendix D and Appendix E.

- Study conclusion of the booster phase
- After study end, one dose Mencevax™ ACWY will be offered to the Tritanrix™-HepB/Hiberix™ control group to protect them against meningococcal diseases of these serogroups: Mencevax™ ACWY will be given when the child is two years of age or, if a meningitis epidemic occurs, earlier in the second year of life.

Extended Safety Follow-Up Phase: Retrospective recording of SAE(s)

The parents/guardians of all study subjects will be contacted by the local health staff when their child is at least 24 months of age for a retrospective follow-up of SAE(s) (i.e. occurrence of any SAE(s) since the last study visit [Visit 7] up to the time that subjects are at least 24 months of age). Prior to initiating the study procedures for this safety follow-up phase, an informed consent will be obtained from the parents/guardians of the child after explaining the purpose of this contact.

The study personnel will contact the parents/guardians of the subjects and will question them in a RETROSPECTIVE manner if the subject has suffered any unreported SAE(s) in the period between the last study visit (study conclusion of the booster phase) and this follow-up contact; the study personnel will also consult the child health card whenever available. If SAE(s) had occurred, further investigations will be performed on the subject's hospital/health centre records (if available). Details of these SAE(s) will be reported on the SAE forms. If a death had occurred, a verbal autopsy report will be obtained whenever possible.

As subjects will be at least two years of age at this safety follow-up visit, the study staff will also remind parents/guardians that the children part of the control group and those with sub-optimal response to the MenA and/or MenC component of the vaccines given to the DTPw-HepB/Hib-MenAC group, will be offered a dose of Mencevax™ ACWY vaccine to protect them against meningococcal diseases (serogroups A, C, W and Y).

This will be the study conclusion for the extended safety follow-up phase.

(Amendment 7: 12 April 2006)

5.5 Sample handling and analysis

5.5.1 Treatment and storage of biological samples

See Appendix D of the protocol for details of treatment and storage of biological samples.

See Appendix E for instructions for shipment of biological samples.

See Appendix H for instructions on collection and analysis of throat swabs**.**

5.5.2 Laboratory assays

All serological assays will be performed at GlaxoSmithKline Biologicals' central laboratory or in a validated laboratory designated by GlaxoSmithKline Biologicals using standardized, validated procedures with adequate controls.

For all subjects, a 3.5 ml sample of whole venous blood will be collected at visits 1, 4, 6 and 7 using tubes with serum separator. Since the investigator has suggested that the collection of this amount of blood at Visit 1 may be technically difficult in such small infants, the quantity collected at this visit can be reduced to 2.0 ml, as a strict minimum, however this will not allow for any validation/retesting etc that may be required when the testing is performed. After blood centrifugation and serum separation, samples will be stored at −20°C until collection by the sponsor. The aliquots of serum (approximately 1.5 ml) will be sent to GlaxoSmithKline Biologicals for the following tests according to the serology plan (refer to Section 5.5.3):

1. Functional anti-meningococcal serogroups A and C activity (SBA-MenA and SBA-MenC) will be determined by a serum bactericidal test according to the CDC protocol [27] using rabbit complement. The cut-off of the test is a dilution of 1:8. Titers will be expressed as the reciprocal of the dilution resulting in 50% inhibition. The use of 1:8 and 1:128 as SBA endpoints when using rabbit complement for SBA-MenC has been discussed at a recent WHO conference, they are now being used in the literature to report on the immunogenicity of meningococcal C conjugate vaccines [28] More recently, efficacy data from postlicensure surveillance has been shown to validate the SBA-MenC 1:8 cut-off as a serological correlate of protection for meningococcal serogroup C conjugate vaccines[29].

2. Meningococcal polysaccharide C specific IgG will be measured by ELISA; the assay is based on the CDC protocol [30]. The assay cut-off is 0.30 µg/ml. The use of the ELISA endpoint of 2 µg/ml is based on the correlate of protection proposed for the plain meningococcal C polysaccharide vaccine. The same cut-offs will be employed for meningococcal A polysaccharide.

3. Total antibodies to the Hib polysaccharide PRP will be measured by ELISA using an in-house assay. The cut-off is 0.15 µg/ml.

4. Specific antibodies against diphtheria and tetanus toxoid will be measured by ELISA techniques. The cut-off of the tests is 0.1 IU/ml [31, 32]. Post-vaccination serum samples with ELISA anti-D antibody concentrations <0.1 IU/ml will be re-tested using a VERO-cell neutralization assay [33, 34]. The cut-off of the VERO-cell assay is 0.016 IU/mL.

5. Anti-*B. pertussis* antibody concentrations will be determined by ELISA using the IgG EIA test kit Lab Systems, and expressed in EL.U/ml with an assay cut-off of 15 EL.U/ml.

6. Antibodies to HBs antigen will be measured using a commercially available kit (AUSAB®EIA test, Abbott Laboratories or equivalent). Antibody concentrations = 10 mIU/ml are considered as protective.

Note: Details of assays for throat swabs are available in Appendix H.

Table 5-4: Laboratory Assays

Antigen	Assay method	Test Kit/ Manufacturer	Assay unit	Assay cut-off	Laboratory
SBA-MenA	Bactericidal assay	in house	dilution for 50% killing	1:8	Rixensart
SBA-MenC	Bactericidal assay	in house	dilution for 50% killing	1:8	Rixensart
PRP	ELISA	in-house	µg/ml	0.15	Rixensart*
PSA	ELISA	in-house	µg/ml	0.3	Rixensart
PSC	ELISA	in-house	µg/ml	0.3	Rixensart
Diphtheria	ELISA	in-house	IU/ml	0.1	Rixensart*
Tetanus	ELISA	in-house	IU/ml	0.1	Rixensart*
BP	ELISA	commercial	EL.U/ml	15	Rixensart*
HBs	ELISA	commercial	mIU/ml	10	Rixensart*
Diphtheria	Vero-cell	in house	IU/ml	0.016	Rixensart

* or in a validated laboratory designated by GlaxoSmithKline Biologicals

5.5.3 Serology plan

Pre-vaccination (Day 0) serum samples will be tested in all subjects for antibodies against SBA-MenA, SBA-MenC, anti-PSA, anti-PSC, diphtheria toxoid, pertussis antigen and recombinant DNA hepatitis B surface antigen (no pre-vaccination testing of anti-TT and anti-PRP is foreseen as immunogenicity results of the previous clinical study were highest for these two antibodies).

Post-vaccination III and pre-booster serum samples will be tested in all subjects for antibodies against all vaccine antigens (SBA-MenA, SBA-MenC, anti-PRP, anti-PSA, anti-PSC, diphtheria and tetanus toxoids, pertussis antigen and recombinant DNA hepatitis B surface antigen).

The post-booster serum sample taken from all children will be tested for SBA-MenA and SBA MenC, anti-PSA and anti-PSC antibodies.

In case of insufficient blood sample volume to perform assays for all antibodies, they will be analyzed according to the following priority ranking:

- SBA-MenA
- SBA-MenC
- anti-PRP (only for Post vacc III and pre-booster)
- anti-diphtheria (Vero-cell assay only when required for Post vacc III and Pre-booster)
- anti-HBs
- anti-tetanus, (only for Post vacc III and pre-booster)
- anti-BP
- anti-PSA
- anti-PSC

Any additional serology on antigens contained in the study vaccines may be performed if deemed necessary by GlaxoSmithKline Biologicals, or if any findings in the present study or in other studies necessitate investigation of the immunogenicity of the vaccine. In this case, the ranking above may also be changed.

5.5.4 Endpoints for suboptimal response

Suboptimal response will be defined as either of the following:

SBA-MenA	< 1:8
SBA-MenC	< 1:8
anti-PRP concentration	< 0.15 µg/ml
anti-HBs concentration	< 10 mIU/ml
anti-BP concentration	< 15 EL.U/ml
anti-diphtheria concentration	< 0.016 (Vero-cell assay)
anti-tetanus concentration	< 0.1 IU/ml

Subjects who demonstrate a suboptimal response to one or more of the following antigen(s) – D, T, Pw, HBV or Hib one month after the primary series will receive a booster dose of licensed Tritanrix™-HepB/Hiberix™ vaccine in the second year of life.

Subjects who demonstrate a suboptimal response to the MenA or MenC antigens will receive a dose of Mencevax™ ACWY to be provided by GSK Biologicals. Mencevax™ ACWY will be given when the child is two years of age or, if a meningitis epidemic occurs, earlier in the second year of life.

6 Investigational Products and Administration

6.1 Study Vaccines

All candidate vaccines to be used have been developed and manufactured by GSK Biologicals.

The Quality Control Standards and Requirements for each candidate vaccine are described in separate release protocols and the required approvals have been obtained.

Commercial vaccines are assumed to comply with the specifications given in the manufacturer's Summary of Product Characteristics.

6.1.1 GSK Biologicals' *Haemophilus influenzae* type b meningococcal AC-TT (Hib-MenAC) conjugate vaccine

One lot of the vaccine will be used. The vaccine will be supplied as a white freeze dried pellet in monodose vials to be reconstituted with Tritanrix™-HepB before use. Each vial of the Hib-MenAC (5/5/5) vaccine needs to be reconstituted with 2 vials of the Tritanrix™-HepB before use (0.5 ml per vial). Only half of the dose (i.e. 0.5 ml will be administered. For details of the dose dilution see Section 6.1.7. One dose (0.5 ml) of the reconstituted vaccine contains the following components:

Haemophilus influenzae type b capsular polysaccharide (PRP) conjugated to tetanus toxoid	:	2.5 µg
Neisseria meningitidis A capsular polysaccharide conjugated to tetanus toxoid	:	2.5 µg
Neisseria meningitidis C capsular polysaccharide conjugated to tetanus toxoid	:	2.5 µg

6.1.2 GSK Biologicals' combined diphtheria-tetanus-whole cell *Bordetella pertussis*-hepatitis B (DTPw-HBV) vaccine: Tritanrix™-HepB

The vaccine will be supplied as a whitish liquid in monodose vials. One dose (0.5 ml) contains the following components:

Diphtheria toxoid	:	not less than 30 IU (7.5 Lf)
Tetanus toxoid	:	not less than 60 IU (3.25 Lf)
Inactivated *Bordetella pertussis* (whole cell)	:	not less than 4 IU (15 OU)
Recombinant HbsAg protein	:	10 µg
Aluminium (as salts)	:	0.63 mg
2-phenoxyethanol (as preservative)	:	0.50 mg
Thiomersal	:	0.025 mg
in a sterile saline solution		

6.1.3 GSK Biologicals' *Haemophilus influenzae* type b conjugate vaccine: Hiberix™

The vaccine will be supplied as a white freeze-dried pellet in monodose vials to be reconstituted with one 0.5 ml vial of Tritanrix™-HepB before use. One dose (0.5 ml) of the reconstituted vaccine contains the following components:

Haemophilus influenzae type b capsular polysaccharide conjugated to tetanus toxoid	:	10 µg

Refer to Appendix G for details of vaccine supplies.

6.1.4 GSK Biologicals' Mencevax™ AC vaccine, used as booster vaccine

Mencevax™ AC is a commercial vaccine which is manufactured by GSK Biologicals. The vaccine will be supplied as a freeze dried pellet to be reconstituted before use with 1.0 ml of the appropriate diluent, supplied in 2 separate vials or ampoules each containing 0.5 ml of diluent. One dose (1 ml) of the reconstituted vaccine will contain:

Components	Content
Polysaccharide A	50 µg
Polysaccharide C	50 µg

Note: Only one fifth of a dose of Mencevax™ AC (0.2 ml) will be administered to each subject as detailed below:

After reconstitution, 0.8 ml (four fifths) of a dose will be discarded and the remaining 0.2 ml, i.e., one fifth (1/5) of a dose, will be administered.

6.1.5 Commercial Measles and Yellow Fever vaccines

The Measles and Yellow Fever vaccines used will be those available commercially in Ghana at the time of the study.

6.1.6 Dosage and administration

An overview of the vaccines to be administered during the study is provided in Table 6-1.

Table 6-1: Dosage and Administration

Visit	Vaccination	Dose	Study Group	Vaccine	Route	Site	Side	Location
1, 2, 3	Diphtheria, Tetanus, *Bordetella pertussis*, hepatitis B, Hib *N.meningitidis* of serogroups A and C	3	Tritanrix™-HepB/ Hib-MenAC	Tritanrix™-HepB/ Hib-MenAC	IM	T	L	U
1, 2, 3	Diphtheria, Tetanus, *Bordetella pertussis*, hepatitis B, Hib	3	Tritanrix™-HepB/ Hiberix™	Tritanrix™-HepB/ Hiberix™	IM	T	L	U
5	Measles,<br>Yellow fever	1	All Subjects	Commercially available vaccines	IM/SC<br>IM	D<br>D	L/R<br>R	U<br>U
6	Antibody persistence/ immune memory	1	All Subjects	1/5th of dose of Mencevax™ AC	IM	T	L	U

Intramuscular (IM)
Thigh (T)
Left (L)
Right (R)
Deltoid (D)
Upper (U)
Subcutaneous (SC)

Injection technique

In order to ensure proper intramuscular injection of the study vaccines, a needle of at least 1 inch (2.54 cm) length will be used. The following injection technique is recommended[35]:

The needle should be inserted in the upper lateral quadrant of the thigh, directed inferiorly at an angle of 45 degrees with the long axis of the leg, and posteriorly at a 45-degree angle to the tabletop, with the subject supine. During the injection, the tissues of the injection site are compressed with the free hand, increasing the penetrable muscle mass and stabilising the extremity.

The vaccinees will be observed closely for at least 30 minutes following the administration of vaccines, with appropriate medical treatment readily available in case of a rare anaphylactic reaction.

6.1.7 Extemporaneous mixing of Tritanrix™-HepB and Hib-MenAC vaccines or Hiberix™

Tritanrix™-HepB and Hiberix™

The liquid Tritanrix™-HepB vaccine should always be shaken before use. The full content of the Tritanrix™-HepB vaccine vial should be extracted and injected into the vial containing the lyophilized Hiberix™ vaccine. The vial should be agitated until the lyophilized vaccine pellet has completely dissolved. The mixed vaccines will appear white. The reconstituted mixed vaccines should be used promptly after reconstitution (within 30 minutes):

- withdraw **one dose of 0.5 ml** of the mixed vaccines from the vial into the syringe;
- a new needle should be used for injection;

One dose (0.5 ml) of the mixed vaccines should be administered by intramuscular injection into the anterolateral quadrant of the left thigh.

Tritanrix™-HepB and Hib-MenAC : dose dilution to provide Hib-MenAC (2.5/2.5/5.5)

The Hib-MenAC (2.5/2.5/2.5) formulation containing 2.5 µg of each polysaccharide will be used in this study. This will be obtained by dilution of the supplied Hib-MenAC (5/5/5) vaccine that contains 5µg of each polysaccharide. Each vial of the Hib-MenAC (5/5/5) vaccine needs to be reconstituted with 2 vials of the Tritanrix™-HepB before use. The liquid Tritanrix™-HepB vaccine should always be shaken before use. The full content of <u>two monodose vials of Tritanrix™-HepB</u> vaccine should be extracted and injected into the vial containing the lyophilized Hib-MenAC (5/5/5) vaccine. The vial

should be agitated until the lyophilized vaccine pellet has completely dissolved. The mixed vaccines will appear white. The reconstituted mixed vaccines should be used promptly after reconstitution (within 30 minutes):

- withdraw one dose of 0.5 ml of the mixed vaccines from the vial;
- a new needle should be used for injection;

One dose (0.5 ml) of the mixed vaccines should be administered by intramuscular injection into the anterolateral quadrant of the left thigh.

Note: The reconstituted vaccine remaining in the vial after 1 dose (0.5 ml) has been withdrawn is to be retained by the investigator for purposes of vaccine accountability and should not be reused. See Appendix G for more details.

6.2 Storage

All vaccines must be stored in a safe and locked place with no access for unauthorized personnel. They must be kept in the refrigerator (+2°C to +8°C/ 36°F to 46°F) and must not be frozen. Storage temperature should be monitored and documented **at least once per day**. It is advisable to have a back-up refrigerator/ freezer in case of power failure/ breakdown. Procedures must be in place to ensure that the vaccine is kept at the indicated temperature range at all times.

The study monitor must be contacted if the vaccines become frozen or if refrigeration fails.

Storage conditions for transport of vaccines from country medical department or dispatch center to study sites or between sites are described in Appendix G.

6.3 Treatment allocation and randomization

The target sample size is 280 enrolled subjects (220 evaluable for immunogenicity analysis, with 110 subjects in each group).

The randomization will be performed in GlaxoSmithKline Biologicals, Rixensart, using a standard Statistical Analysis System (SAS®) program.

A randomization list will be generated using a randomization blocking scheme (1:1 ratio). This list will be used to ensure balance between treatments and will be used to number the vaccines: a randomization number will uniquely identify the vaccine doses to be administered to the same subject.

The vaccine doses will be distributed to the study center while respecting the randomization block size. On Day 0 (Month 0), subjects will be administered the vaccine dose with the lowest number still available at the study center. The vaccine number will also be used as subject identification for all data collected under the study.

6.4 Method of blinding and breaking the study blind

The study will be double blind. The study vaccine and the control vaccine will have the same whitish appearance after reconstitution. However, since the vaccines used in this study are different in their packaging volumes (Tritanrix™-HepB and Hiberix™ = 1 vial of 0.5ml of each vaccine, Tritanrix™-HepB and Hib-MenAC = 2 vials of 0.5 ml of Tritanrix™-HepB and 1 vial of 0.5 ml of Hib-MenAC), special precautions will be taken to ensure blinding. For each vaccination during the course of the study, a Syringe Filler and Vaccinator will work as a vaccination team: the Syringe Filler will prepare the vaccine for administration to a specific subject and the Vaccinator will administer the vaccine to each subject, according to the investigator's SOP. To ensure blinding, a vaccination team will perform no other function in the study. The subject's parent/guardian as well as those responsible for the evaluation of safety or immunogenicity study parameters will all be unaware which vaccine preparation was administered to a particular subject.

A set of individual codes will be held at the GSK Biologicals' Central Safety Office and by the Local Safety Monitor. The code will be broken by the GSK Clinical Safety physician or Local Safety Monitor only in the case of medical events that the investigator/physician in charge of the subject feels cannot be treated without knowing the identity of the study vaccine.

GSK Biologicals' policy (incorporating ICH E2A guidance, EU Clinical Trial Directive and Federal Regulations) is to unblind any serious adverse event (SAE) report associated with the use of the investigational product, which is unexpected and attributable/suspected, prior to regulatory reporting. The Clinical Safety physician is responsible for unblinding the treatment assignment in accordance with specified time frames for expedited reporting of SAEs (Refer to Section 8.9).

6.5 Replacement of unusable vaccine doses

Additional vaccine doses will be provided to replace those that are unusable (see Appendix G for details of supplies).

In addition to the vaccine doses provided for the planned number of subjects, at least 5% additional doses of Tritanrix™-HepB and Hiberix™ will be supplied. In case a vaccine dose is broken or unusable, the investigator should replace it with a replacement vaccine dose. Although GlaxoSmithKline Biologicals need not be notified immediately in these cases, documentation of the use of the replacement vaccine must be recorded by the investigator on the vaccine administration page of the CRF and on the vaccine accountability form.

6.6 Packaging

See Appendix G.

6.7 Vaccine accountability

See Appendix G.

6.8 Concomitant medication/treatment

6.8.1 Recording of concomitant medication/treatment during the primary phase of the study

At each study visit/contact, the investigator should question the subject's parents/guardian about any medication(s) taken.

All concomitant medication, with the exception of vitamins and/or dietary supplements, administered at ANY time during the period starting with administration of each dose and ending one month (minimum 30 days) after each dose are to be recorded with generic name of the medication (trade names are allowed for combination drugs, i.e., multi-component drugs), medical indication, total daily dose, route of administration, start and end dates of treatment.

Any treatments and/or medications specifically contraindicated, e.g., any immunoglobulins, other blood products and any immune modifying drugs administered since birth or at any time during the study period are to be recorded with generic name of the medication (trade names are allowed for combination drugs only), medical indication, total daily dose, route of administration, start and end dates of treatment. Refer to Sections 4.3 and 4.4.

Any meningococcal vaccine given **to the mother** of the child during the year preceding the first vaccine dose given to the child should be recorded in the CRF with trade name, route of administration and date(s) of administration.

Any vaccine not foreseen in the study protocol administered since birth and throughout the study period are to be recorded with trade name, route of administration and date(s) of administration, with the exception of OPV. Refer to Sections 4.3 and 4.4.

Any concomitant medication administered prophylactically in anticipation of reaction to the vaccination must be recorded in the CRF with generic name of the medication (trade names are allowed for combination drugs only), total daily dose, route of administration, start and end dates of treatment and coded as ‘Prophylactic’.

Concomitant medication administered for the treatment of an AE or SAE must be recorded in the CRF with generic name of the medication (trade names are allowed for combination drugs only), medical indication (including which AE/SAE), total daily dose, route of administration, start and end dates of treatment. Refer to Section 8.2 for definition of SAE.

6.8.2 Recording of concomitant medication/treatment during the booster phase of the study

At Visit 5, the investigator will question the parents/guardians in a RETROSPECTIVE manner if the subject received any relevant medication/treatment or vaccine in the time period since the last study visit of the primary phase of the study (Visit 4). Relevant vaccines will include meningococcal vaccines and investigational or non registered vaccines and will be recorded with trade name, route of administration and date(s) of administration. Relevant medications/ treatments will include any investigational or non-registered product(s), any immunosuppressant(s) or other immune-modifying drug(s), immunoglobulins and/or any blood products. All such medication/treatment is to be recorded with generic name of the medication (trade named are allowed for combination drugs, i.e. multi-component drugs), medical indication, total daily dose, route of administration, start and end dates of treatment.

In addition, all concomitant medication, with the exception of vitamins and/or dietary supplements, administered at ANY time during the period starting with administration of each dose and ending one month (minimum 30 days) after each dose are to be recorded with generic name of the medication (trade names are allowed for combination drugs, i.e., multi-component drugs), medical indication, total daily dose, route of administration, start and end dates of treatment.

Any treatments and/or medications specifically contraindicated, e.g., any immunoglobulins, other blood products and any immune modifying drugs administered at any time during the study period are to be recorded with generic name of the medication (trade names are allowed for combination drugs only), medical indication, total daily dose, route of administration, start and end dates of treatment. Refer to Section 4.4.

Any vaccine not foreseen in the study protocol administered throughout the study period are to be recorded with trade name, route of administration and date(s) of administration, with the exception of OPV that can be given throughout the study. Refer to Section 4.4.

Any concomitant medication administered prophylactically in anticipation of reaction to the vaccination must be recorded in the CRF with generic name of the medication (trade names are allowed for combination drugs only), total daily dose, route of administration, start and end dates of treatment and coded as 'Prophylactic'.

Concomitant medication administered for the treatment of an AE or SAE must be recorded in the CRF with generic name of the medication (trade names are allowed for combination drugs only), medical indication (including which AE/SAE), total daily dose, route of administration, start and end dates of treatment. Refer to Section 8.2 for definition of SAE.

7 Health Economics

Not applicable.

8 Adverse Events, Serious Adverse Events and vaccine failure

The investigator is responsible for the detection and documentation of events meeting the criteria and definition of an adverse event (AE) or serious adverse event (SAE) as provided in this protocol. During the study, when there is a safety evaluation, the investigator or site staff will be responsible for detecting AEs and SAEs, as detailed in this section of the protocol.

Each subject's parents/guardians will be instructed to contact the investigator immediately should the subject manifest any signs or symptoms they perceive as serious.

8.1 Definition of an adverse event

An AE is any untoward medical occurrence in a clinical investigation subject, temporally associated with the use of a medicinal product, whether or not considered related to the medicinal product.

An AE can therefore be any unfavourable and unintended sign (including an abnormal laboratory finding), symptom, or disease (new or exacerbated) temporally associated with the use of a medicinal product. For marketed medicinal products, this also includes failure to produce expected benefits (i.e., lack of efficacy), abuse or misuse.

Examples of an AE include:

- Significant or unexpected worsening or exacerbation of the condition/indication under study. See Section 8.3 'Lack of Efficacy' for additional information.
- Exacerbation of a chronic or intermittent pre-existing condition including either an increase in frequency and/or intensity of the condition.
- New conditions detected or diagnosed after investigational product administration even though it may have been present prior to the start of the study.
- Signs, symptoms, or the clinical sequelae of a suspected interaction.
- Signs, symptoms, or the clinical sequelae of a suspected overdose of either investigational product or a concurrent medication (overdose per se should not be reported as an AE/SAE).
- Signs, symptoms temporally associated with vaccine administration.

Examples of an AE DO NOT include:

- Medical or surgical procedure (e.g., endoscopy, appendectomy); the condition that leads to the procedure is an AE.
- Situations where an untoward medical occurrence did not occur (social and/or convenience admission to a hospital).
- Anticipated day-to-day fluctuations of pre-existing disease(s) or condition(s) present or detected at the start of the study that do not worsen.
- The disease/disorder being studied, or expected progression, signs, or symptoms of the disease/disorder being studied, unless more severe than expected for the subject's condition.

AEs may include pre- or post-treatment events that occur as a result of protocol-mandated procedures (i.e., invasive procedures, modification of subject's previous therapeutic regimen).

N.B. AEs to be recorded as endpoints (solicited events) are described in Section 8.5.1. All other AEs will be recorded as UNSOLICITED AEs.

Example of events to be recorded in the medical history section of the CRF:

- Pre-existing conditions or signs and/or symptoms present in a subject prior to the start of the study (i.e. prior to the first study procedure) should be recorded in the medical history section of the subject's CRF as instructed by the GSK Biologicals' Study Monitor.

8.2 Definition of a serious adverse event

A serious adverse event (SAE) is any untoward medical occurrence that:

a) results in death,

b) is life-threatening,

NOTE: The term 'life-threatening' in the definition of 'serious' refers to an event in which the subject was at risk of death at the time of the event. It does not refer to an event, which hypothetically might have caused death, if it were more severe.

c) requires hospitalization or prolongation of existing hospitalization,

NOTE: In general, hospitalization signifies that the subject has been detained (usually involving at least an overnight stay) at the hospital or emergency ward for observation and/or treatment that would not have been appropriate in the physician's office or out-patient setting. Complications that occur during hospitalization are AEs. If a complication prolongs hospitalization or fulfils any other serious criteria, the event is serious. When in doubt as to whether "hospitalization" occurred or was necessary, the AE should be considered serious.

Hospitalization for elective treatment of a pre-existing condition that did not worsen from baseline is not considered an AE.

d) results in disability/incapacity,

NOTE: The term disability means a substantial disruption of a person's ability to conduct normal life functions. This definition is not intended to include experiences of relatively minor medical significance such as uncomplicated headache, nausea, vomiting,

diarrhoea, influenza, and accidental trauma (e.g. sprained ankle) which may interfere or prevent everyday life functions but do not constitute a substantial disruption.

e) is a congenital anomaly/birth defect in the offspring of a study subject,

f) medical or scientific judgement should be exercised in deciding whether reporting is appropriate in other situations, such as important medical events that may not be immediately life-threatening or result in death or hospitalization but may jeopardize the subject or may require medical or surgical intervention to prevent one of the other outcomes listed in the above definition. These should also be considered serious. Examples of such events are invasive or malignant cancers, intensive treatment in an emergency room or at home for allergic bronchospasm, blood dyscrasias or convulsions that do not result in hospitalization.

8.3 Lack of efficacy

"Lack of efficacy" per se will not be reported as an AE. The signs and symptoms or clinical sequelae resulting from lack of efficacy will be reported if they fulfil the AE or SAE definition (including clarifications). Any case of diphtheria, tetanus, pertussis, hepatitis B, invasive diseases due to Haemophilus influenzae type b, measles, yellow fever or Neisseria meningitidis of serogroup A and C will be reported to the Independent Data Monitoring Committee (IDMC).

8.4 Clinical laboratory parameters and other abnormal assessments qualifying as adverse events and serious adverse events

Abnormal laboratory findings (e.g., clinical chemistry, haematology, urinalysis) or other abnormal assessments (e.g., ECGs, X-rays, vital signs, etc.) that are judged by the investigator to be clinically significant will be recorded as AEs or SAEs if they meet the definition of an AE, as defined in Section 8.1 or SAE, as defined in Section 8.2. Clinically significant abnormal laboratory findings or other abnormal assessments that are detected during the study or are present at baseline and significantly worsen following the start of the study will be reported as AEs or SAEs. However, clinically significant abnormal laboratory findings or other abnormal assessments that are associated with the disease being studied, unless judged by the investigator as more severe than expected for the subject's condition, or that are present or detected at the start of the study and do not worsen, will not be reported as AEs or SAEs.

The investigator will exercise his or her medical and scientific judgement in deciding whether an abnormal laboratory finding or other abnormal assessment is clinically significant.

8.5 Time period, frequency, and method of detecting adverse events and serious adverse events

All AEs occurring within one month (minimum 30 days) following administration of each dose of vaccine/ comparator must be recorded on the Adverse Event form in the subject's CRF, irrespective of severity or whether or not they are considered vaccination-related.

The standard time period for collecting and recording SAEs will begin at randomization or the first receipt of vaccine/ comparator and will end one month (minimum 30 days) following administration of the last dose of study vaccine/ comparator for each subject. See Section 8.8 for instructions for reporting and recording SAEs.

Additionally, in order to fulfil international reporting obligations, SAEs that are related to study participation (e.g. procedures, invasive tests, a change from existing therapy) or are related to a concurrent medication will be collected and recorded from the time the subject consents to participate in the study until she/he is discharged.

- The investigator will inquire about the occurrence of AEs/SAEs at every visit/contact during the study.

All AEs either observed by the investigator or one of his clinical collaborators or reported by the subject’s parent/guardian spontaneously or in response to a direct question will be evaluated by the investigator. AEs not previously documented in the study will be recorded in the Adverse Event form within the subject's CRF. The nature of each event, date and time (where appropriate) of onset, outcome, intensity and relationship to vaccination should be established. Details of any corrective treatment should be recorded on the appropriate page of the CRF. Refer to Section 6.8.

As a consistent method of soliciting AEs, the subject’s parent/guardian should be asked a non-leading question such as:

"Has your child acted differently or felt different in any way since receiving the vaccine or since the last visit?"

N.B. The investigator should record only those AEs having occurred within the time frame defined above.

AEs already documented in the CRF, i.e. at a previous assessment, and designated as ‘ongoing’ should be reviewed at subsequent visits, as necessary. If these have resolved, the documentation in the CRF should be completed.

N.B. If an AE changes in frequency or intensity during the specified reporting period, a new record of the event will be entered.

When an AE/SAE occurs, it is the responsibility of the investigator to review all documentation (e.g., hospital progress notes, laboratory, and diagnostics reports) relative to the event. The investigator will then record all relevant information regarding an AE/SAE on the CRF or SAE Report Form as applicable. It is not acceptable for the investigator to send photocopies of the subject’s medical records to GSK Biologicals in lieu of the appropriate completed AE/SAE pages. However, there may be instances when copies of medical records for certain cases are requested by GSK Biologicals. In this instance, all subject identifiers will be blinded on the copies of the medical records prior to submission to GSK Biologicals.

The investigator will attempt to establish a diagnosis of the event based on signs, symptoms, and/or other clinical information. In such cases, the diagnosis should be documented as the AE/SAE and not the individual signs/symptoms.

There will be a retrospective follow-up of SAE(s) (i.e. occurrence of any SAE(s) since the last study visit [Visit 7] up to the time that subjects are at least 24 months of age).

(Amendment 7: 12 April 2006)

8.5.1 Solicited adverse events

Local (injection site) adverse events

A four-day follow-up (day 0-3) of solicited local adverse events at each injection site will be performed after vaccination. Data concerning the following adverse events will be solicited using diary cards provided by the sponsor.

Table 8-1: Solicited local adverse events

Pain at injection site
Swelling at injection site

General (systemic) adverse events
A four-day follow-up (day 0-3) of solicited general adverse events will be performed after vaccination. Data concerning the following adverse events will be solicited using diary cards provided by the sponsor.

Table 8-2: Solicited general adverse events

Drowsiness
Fever
Irritability/ Fussiness
Loss of appetite

N.B. Temperature will be recorded by axillary route by the field worker during the home visits. Should temperature measurement additionally be performed at another time of day, the highest temperature will be recorded.

8.6 Evaluating adverse events and serious adverse events

Field workers will be trained on the definition of adverse events including SAEs as per protocol definition, under the supervision of the principal investigator.

8.6.1 Assessment of intensity

Intensity of the following AEs will be assessed as described:

Table 8-3: Intensity scales for solicited symptoms

Adverse Event	Intensity grade	Parameter
Pain at injection site	0	Absent
	1	Minor reaction to touch
	2	Cries/protests on touch
	3	Cries when limb is moved/spontaneously painful
Swelling at injection site		Record greatest surface diameter in mm
Fever*		Record temperature in °C
Irritability/Fussiness	0	Behavior as usual
	1	Crying more than usual/ no effect on normal activity
	2	Crying more than usual/ interferes with normal activity
	3	Crying that cannot be comforted/ prevents normal activity
Drowsiness	0	Behavior as usual
	1	Drowsiness easily tolerated
	2	Drowsiness that interferes with normal activity
	3	Drowsiness that prevents normal activity
Loss of appetite	0	Normal
	1	Eating less than usual/ no effect on normal activity
	2	Eating less than usual/ interferes with normal activity
	3	Not eating at all

*Fever is defined as axillary temperature ≥ 37.5°C.

The maximum intensity of local injection site swelling will be scored at GSK Biologicals as follows:

**0**	**:**	none
**1**	**:**	>0 - ≤ 10 mm
**2**	**:**	>10 - ≤ 30 mm
**3**	**:**	>30 mm

The maximum intensity of fever* will be scored at GSK Biologicals as follows:

		**Axillary**
**0**	**:**	<37.5°C
**1**	**:**	≥37.5°C - ≤38.5°C
**2**	**:**	>38.5°C - ≤39.5°C
**3**	**:**	>39.5°C

* Temperature will be measured by the axillary route.

For each solicited symptom, the subjects’ parents/guardians will be asked if they sought medical advice (i.e., contact with a member of medical personnel) for this symptom.

The investigator will make an assessment of intensity for all other AEs, i.e. unsolicited symptoms, including SAEs reported during the study. The assessment will be based on the investigator’s clinical judgement. The intensity of each AE and SAE recorded in the CRF or SAE Report Form, as applicable, should be assigned to one of the following categories:

1 (mild)	=	An AE which is easily tolerated by the subject, causing minimal discomfort and not interfering with everyday activities.
2 (moderate)	=	An AE which is sufficiently discomforting to interfere with normal everyday activities.
3 (severe)	=	An AE which prevents normal, everyday activities. (In a young child, such an AE would, for example, prevent attendance at school/ kindergarten/ a day-care center and would cause the parents/ guardians to seek medical advice.)

An AE that is assessed as Grade 3 (severe) should not be confused with a SAE. Grade 3 is a category utilized for rating the intensity of an event; and both AEs and SAEs can be assessed as Grade 3. An event is defined as 'serious' when it meets one of the pre-defined outcomes as described in Section 8.2.

8.6.2 Assessment of causality

The investigator is obligated to assess the relationship between investigational product and the occurrence of each AE/SAE. The investigator will use clinical judgement to determine the relationship. Alternative causes, such as natural history of the underlying diseases, concomitant therapy, other risk factors and the temporal relationship of the event to the investigational product will be considered and investigated. The investigator will also consult the Investigator Brochure and/or Product Information, for marketed products, in the determination of his/her assessment.

There may be situations when a SAE has occurred and the investigator has minimal information to include in the initial report to GSK Biologicals. However, it is very important that the investigator always makes an assessment of causality for every event prior to transmission of the SAE Report Form to GSK Biologicals. The investigator may change his/her opinion of causality in light of follow-up information, amending the SAE Report Form accordingly. The causality assessment is one of the criteria used when determining regulatory reporting requirements.

In case of concomitant administration of multiple vaccines, it may not be possible to determine the causal relationship of general AEs to the individual vaccines administered. The investigator should, therefore, assess whether the AE could be causally related to vaccination rather than to the individual vaccines.

All solicited local (injection site) reactions will be considered causally related to vaccination. Causality of all other AEs should be assessed by the investigator using the following question:

In your opinion, did the vaccine(s) possibly contribute to the adverse event?

NO : The AE is not causally related to administration of the study vaccine(s). There are other, more likely causes and administration of the study vaccine(s) is not suspected to have contributed to the AE.

YES : There is a reasonable possibility that the vaccine contributed to the AE.

Non-serious and serious AEs will be evaluated as two distinct events. If an event meets the criteria to be determined “serious” (see Section 8.2 for definition of serious adverse event), it will be examined by the investigator to the extent to be able to determine ALL contributing factors applicable to each serious adverse event.

Other possible contributors include:
- Medical history
- Other medication
- Protocol required procedure
- Other procedure not required by the protocol
- Lack of efficacy of the vaccine
- Erroneous administration
- Other cause (specify)

8.7 Follow-up of adverse events and serious adverse events and assessment of outcome

After the initial AE/SAE report, the investigator is required to proactively follow each subject and provide further information to GSK Biologicals on the subject’s condition.

All AEs and SAEs documented at a previous visit/contact and designated as not recovered/not resolved or recovering/resolving will be reviewed at subsequent visits/contacts.

Investigators will follow-up subjects:

(1) with SAEs or subjects withdrawn from the study as a result of an AE, until the event has resolved, subsided, stabilized, disappeared, the event is otherwise explained, or the subject is lost to follow-up;

(2) or, in the case of other non-serious AEs, until they complete the study or they are lost to follow-up.

Clinically significant laboratory abnormalities will be followed up until they have returned to normal, or a satisfactory explanation has been provided. Reports relative to the subsequent course of an AE noted for any subject must be submitted to the Study Monitor.

GSK Biologicals may request that the investigator perform or arrange for the conduct of supplemental measurements and/or evaluations to elucidate as fully as possible the nature and/or causality of the AE or SAE. The investigator is obliged to assist. If a subject dies during participation in the study or during a recognized follow-up period, GSK Biologicals will be provided with a copy of any available post-mortem findings, including histopathology. In the case of a death, supplementary information will be gained using the verbal autopsy technique. This will be conducted according to previously published methods and detailed in the SOPs on file with the investigators. The Verbal Autopsy Questionnaire will be completed and transmitted by the investigator to GSK Biologicals, WHO and the Local Safety Monitor, in addition to the SAE report, irrespective of relationship to vaccination and whether a written autopsy was performed or not. The Standard Verbal Autopsy Questionnaire will not replace a written autopsy.

New or updated information will be recorded on the originally completed SAE Report Form, with all changes signed and dated by the investigator. The updated SAE report form should be resent to GSK Biologicals within 24 hours of receipt of the follow-up information as outlined in Section 8.8.1.

All SAEs will be reported within 24 hours by the investigator to GSK Biologicals safety department, to the local safety monitor and to the WHO study contact for SAE reporting. GSK Biologicals will transmit the information to the IDMC according to the operating procedures of the IDMC.

In addition, the investigator will also provide a listing of all systemic grade 3 symptoms suspected to be related to vaccination and any case of diphtheria, tetanus, pertussis, hepatitis B, invasive diseases due to Haemophilus influenzae type b, measles, yellow fever or Neisseria meningitidis of serogroup A and C to the LSM. The Local Safety Monitor will send the listing and information related to the non-serious grade 3 systemic symptoms related with vaccination and non-serious adverse events suggesting a lack of efficacy of the vaccine to the IDMC and the GSK medical monitor (the GSK medical monitor will forward the information to the other members of the Steering Committee) according to the Charter for the IDMC.

Outcome of any non-serious AE occurring within 30 days post-vaccination (i.e. unsolicited AE) or any SAE reported during the entire study will be assessed as:

1 : Recovered/resolved
2 : Not recovered/not resolved
3 : Recovering/resolving
4 : Recovered/resolved with sequelae

8.8 Prompt reporting of serious adverse events to GSK Biologicals, WHO and the local safety monitor

8.8.1 Time frames for submitting serious adverse event reports to GSK Biologicals, WHO and the local safety monitor

SAEs will be reported promptly to the LSM, GSK Biologicals and WHO once the investigator determines that the event meets the protocol definition of an SAE. The investigator or designate will transmit the SAE reports to GSK Biologicals' Study Contact for Serious Adverse Event Reporting and to the WHO study contact for SAE reporting within 24 hours of his/her becoming aware of these events. Additional or follow-up information relating to the initial SAE report, including completed verbal autopsy questionnaire in case of death, is also to be reported to the GSK Biologicals' Study Contact for Serious Adverse Event Reporting and to the WHO study contact for SAE reporting within 24 hours.

GSK Biologicals will transmit the SAEs to the IDMC on a regular basis according to the Charter for the IDMC (see section 5.1.3).

8.8.2 Completion and transmission of serious adverse event reports to GSK Biologicals, WHO and the local safety monitor

Once an investigator becomes aware that a SAE has occurred in a study subject, she/he will report the information to GSK Biologicals and WHO within 24 hours and to the local safety monitor as outlined in Section 8.8.1. The SAE Report Form will always be completed as thoroughly as possible with all available details of the event, signed by the investigator (or designee), and forwarded to GSK Biologicals and WHO within the designated time frames. If the investigator does not have all information regarding an SAE, he/she will not wait to receive additional information before notifying GSK Biologicals and WHO of the event and completing the form. The form will be updated when additional information is received and forwarded to GSK Biologicals and WHO within 24 hours as outlined in Section 8.8.1.

The investigator will always provide an assessment of causality at the time of the initial report as described in Section 8.6.2.

Facsimile (Fax) transmission of the SAE Report Form is the preferred method to transmit this information to the Study Contact for Reporting SAEs. In rare circumstances and in the absence of facsimile equipment, notification by telephone is acceptable, with a copy of the SAE Report Form sent by overnight mail. Initial notification via the telephone does not replace the need for the investigator to complete and sign the SAE Report Form within 24 hours as outlined in Section 8.8.1.

In the event of a death determined by the investigator to be related to vaccination, sending of the fax must be accompanied by telephone call to the Study Contact for Reporting SAEs.

**Study Contacts for Reporting SAEs**
**Medical Monitor at GSK Biologicals**<br>Dr. Dominique Boutriau,<br>***Director, Clinical Research & Development, Meningitis vaccines***<br>GlaxoSmithKline Biologicals,<br>Rue de l'Institut 89, 1330 Rixensart, Belgium<br>Tel: +32.2.656.9120<br>Fax: +32.2.656. ***8044***<br>e-mail: dominique.boutriau@gskbio.com<br><br>**(Amendment 7: 12 April 2006)**<br>**Local Safety Monitor**<br>Dr. John Williams,<br>Navrongo Health Research Center,<br>Ministry of Health, PO Box 114, Navrongo, Ghana<br>Tel: +233 742 22310/ 22380<br>Fax: + 233 742 22320<br>e-mail: jwilliams@navrongo.mimcom.net<br>**Local Safety Monitor Back-up**<br>Dr. Frank Baiden<br>Navrongo Health Research Center,<br>Ministry of Health, PO Box 114, Navrongo, Ghana<br>Tel: + 233 742 22310/ 22380<br>Fax: + 233 742 22320<br>e-mail: fbaiden@navrongo.mimcom.net<br><br>**Study Contact for SAE Reporting at WHO**<br>Dr. Zarifah Reed<br>WHO/IVR/POP<br>WHO/IVB/IVR/POP<br>20 Avenue Appia<br>CH-1211 GENEVE 27<br>Switzerland<br>Tel. +41 22 791 4760<br>Fax. +41 22 791 4860<br>Email: reedz@who.int
Continued on next page

**Study Contacts for Reporting SAEs**
**Back-up Study Contact for Reporting SAEs at GSK Biologicals**<br>***GSK Biologicals Clinical Safety Physician***<br>Tel: +32-2-656 ***88 50***<br>Fax: +32-2-656 80 09***/5116***<br>Mobile phone for 7/7 day availability: +32 477 404 713 ***or 32-(0) 474 53 48 68***<br>**24/24 hour and 7/7 day availability**

(Amendment 7: 12 April 2006)

8.9 Regulatory reporting requirements for serious adverse events

The investigator will promptly report all SAEs to GSK Biologicals in accordance with the procedures detailed in Section 8.8. GSK Biologicals has a legal responsibility to promptly notify, as appropriate, both the local regulatory authority and other regulatory agencies about the safety of a product under clinical investigation. Prompt notification of SAEs by the investigator to the Study Contact for Reporting SAEs is essential so that legal obligations and ethical responsibilities towards the safety of other subjects are met.

The investigator, or responsible person according to local requirements, will comply with the applicable local regulatory requirements related to the reporting of SAEs to regulatory authorities and the IRB/IEC.

Expedited Investigator Safety Reports (EISR) are prepared according to GSK Biologicals policy and are forwarded to investigators as necessary. An EISR is prepared for a SAE that is both attributable to the investigational product and unexpected. The purpose of the EISR is to fulfil specific regulatory and Good Clinical Practice (GCP) requirements, regarding the product under investigation.

An investigator who receives an EISR describing a SAE or other specific safety information from GSK Biologicals will file it with the Investigator Brochure and will notify the IRB/IEC, if appropriate according to local requirements.

8.10 Post study adverse events and serious adverse events

A post-study AE/SAE is defined as any event that occurs outside of the AE/SAE detection period defined in Section 8.5. Investigators are not obligated to actively seek AEs or SAEs in former study participants.

However, if the investigator learns of any SAE, including a death, at any time after a subject has been discharged from the study, and he/she considers the event reasonably related to the investigational product, the investigator will promptly notify the Study Contact for Reporting SAEs.

8.11 Treatment of adverse events

Treatment of any adverse event is at the sole discretion of the investigator. Any AE will be treated for free according to good medical practice and standard of care in Ghana. Standard treatment of the common diseases in the study sites will be available in each facility (hospital, health centres) where the study will take place. Any medication administered for the treatment of an AE should be recorded in the subject's CRF. Refer to Section 6.8.

9 Subject Completion and Withdrawal

9.1 Subject completion

A subject who returns for the concluding visit/is available for the concluding contact foreseen in the protocol is considered to have completed the study.

9.2 Subject withdrawal

Subjects who are withdrawn for AEs must be clearly distinguished from subjects who are withdrawn for other reasons. Investigators will follow subjects who are withdrawn as result of a SAE/AE until resolution of the event (see Section 8.7).

Withdrawals will not be replaced.

9.2.1 Subject withdrawal from the study

From an analysis perspective, a 'withdrawal' from the study is any subject who did not come back for the concluding visit foreseen in the protocol.

A subject qualifies as a 'withdrawal' from the study when no study procedure has occurred, no follow-up has been performed and no further information has been collected for this subject from the date of withdrawal/last contact.

Investigators will make an attempt to contact those subjects who do not return for scheduled visits or follow-up.

Information relative to the withdrawal will be documented on the Study Conclusion page of the CRF. It will be specified which of the following possible reasons were responsible for withdrawal:

- serious adverse event
- non-serious adverse event
- protocol violation (specify)
- consent withdrawal, not due to an adverse event
- moved from the study area
- lost to follow-up
- other (specify)

9.2.2 Subject withdrawal from investigational product

A 'withdrawal' from the investigational product is any subject who does not receive the complete treatment, i.e. when no further planned dose is administered from the date of withdrawal. A subject withdrawn from the investigational product may not necessarily be withdrawn from the study as further study procedures or follow-up may be performed (safety or immunogenicity) if planned in the study protocol.

Information relative to premature discontinuation of the investigational product will be documented on the Vaccine Administration page of the CRF. The investigator will document whether the decision to discontinue further vaccination was made by the the subject's parent or guardian or the investigator and which of the following possible reasons was responsible for withdrawal:

- serious adverse event,
- non-serious adverse event,
- other (specify).

9.3 Extension study

There will be a retrospective follow-up of SAE(s) that occurred since the last study visit [Visit 7] up to the time that subjects are at least 24 months of age.

(Amendment 7: 12 April 2006)

9.4 Screen and baseline failures

Not applicable.

10 Data Evaluation: Criteria for Evaluation of Objectives

Note: Data collected for the ancillary study will be provided to the investigators of the ancillary study for evaluation (refer to Appendix H and Section 5.2).

10.1 Co-primary endpoints

Immunogenicity: post-primary vaccination
In all subjects, one month after the 3rd dose of the primary vaccination:

- Serum bactericidal antibody titer (MenC) ≥1:8 (seropositivity)
- Serum bactericidal antibody titer (MenA) ≥1:8 (seropositivity)
- Anti-PRP concentration ≥ 1 µg/ml (seroprotection)
- Anti-HBs concentration ≥10 mIU/ml (seroprotection)
- Anti-tetanus concentration ≥ 0.1 IU/ml (seroprotection)
- Anti-diphtheria concentration ≥ 0.1 IU/ml by ELISA and, if negative, ≥ 0.016 IU/ml by Vero-cell (seroprotection)
- Concentration of anti-BP antibodies

10.2 Secondary endpoints

Immunogenicity

Pre-primary vaccination
In all subjects, just prior to the administration of the 1st dose of the primary vaccination

- SBA-MenA titer ≥ 1:8 (seropositivity)
- SBA-MenC titer ≥ 1:8 (seropositivity)
- Anti-PSA concentration ≥ 0.3 µg/ml (seropositivity)
- Anti-PSC concentration ≥ 0.3 µg/ml (seropositivity)
- Anti-PSA concentration ≥ 2 µg/ml
- Anti-PSC concentration ≥ 2 µg/ml
- Anti-diphtheria concentration ≥ 0.1 IU/ml (seroprotection)
- Anti-HBs concentration ≥10 mIU/ml (seroprotection)
- Anti-BP concentration ≥ 15 EL.U/ml (seropositivity)
- Concentration/titers of SBA-MenA, SBA-MenC, anti-PSA, anti-PSC, anti-HBs, anti-diphtheria and anti-BP antibodies

Post-primary vaccination:
In all subjects, one month after the 3rd dose of the primary vaccination:
- Anti-PSA concentration ≥ 0.3 µg/ml (seropositivity)
- Anti-PSC concentration ≥ 0.3 µg/ml (seropositivity)
- Anti-PSA concentration ≥ 2 µg/ml
- Anti-PSC concentration ≥ 2 µg/ml
- Anti-PRP concentration ≥ 0.15 µg/ml (seroprotection)
- Anti-BP concentration ≥15 EL.U/ml

Titers/Concentrations of all antibodies but anti-BP (see co-primary endpoints)

Antibody persistence:
In all subjects, at twelve months of age, just prior to the administration of 1/5 of a dose of Mencevax™ AC:
- SBA-MenA titer ≥ 1:8 (seropositivity)
- SBA-MenC titer ≥ 1:8 (seropositivity)
- Anti-PSA concentration ≥ 0.3 µg/ml (seropositivity)
- Anti-PSC concentration ≥ 0.3 µg/ml (seropositivity)
- Anti-PSA concentration ≥ 2 µg/ml
- Anti-PSC concentration ≥ 2 µg/ml
- Anti-PRP concentration ≥ 0.15 µg/ml (seroprotection)
- Anti-PRP concentration ≥ 1 µg/ml (seroprotection)
- Anti-HBs concentration ≥10 mIU/ml (seroprotection)
- Anti-tetanus concentration ≥ 0.1 IU/ml (seroprotection)
- Anti-diphtheria concentration ≥ 0.1 IU/ml by ELISA and, if negative, ≥ 0.016 IU/ml by Vero-cell (seroprotection)
- Anti-BP concentration ≥ 15 EL.U/ml (seropositivity)
- Concentration/titers of SBA-MenA, SBA-MenC, anti-PSA, anti-PSC, anti-PRP, anti-HBs, anti-tetanus, anti-diphtheria and anti-BP antibodies.

Immune memory:
In all subjects, one month after the administration of 1/5 of a dose of Mencevax™ AC:
- SBA-MenA titer ≥ 1:8 (seropositivity)
- SBA-MenC titer ≥ 1:8 (seropositivity)
- Anti-PSA concentration ≥ 0.3 µg/ml (seropositivity)
- Anti-PSC concentration ≥ 0.3 µg/ml (seropositivity)
- Anti-PSA concentration ≥ 2 µg/ml
- Anti-PSC concentration ≥ 2 µg/ml
- Concentration/titers of SBA-MenA, SBA-MenC, anti-PSA and anti-PSC antibodies.

Reactogenicity and safety:
After each vaccine dose:

- Occurrence of solicited symptoms (during the 4-day follow-up period)
  Local: Pain and swelling at the injection site
  General: Drowsiness, Fever, Irritability/ Fussiness, Loss of appetite
- Occurrence of unsolicited symptoms (during the 30-day follow-up period)

Over the full course of the study:

- Occurrence of serious adverse events

10.3 Estimated sample size

The target sample size is 280 enrolled subjects to reach 220 evaluable subjects in the ATP cohort for immunogenicity (110 evaluable subjects in each group). Refer to Section 10.4 for definition of study cohorts.

The co-primary objectives of the study are:
One month after primary vaccination:

- To demonstrate the immunogenicity of Tritanrix™-HepB/Hib-MenAC with respect to SBA-MenA and SBA-MenC.
- To demonstrate that Tritanrix™-HepB/Hib-MenAC is non-inferior to the control vaccine Tritanrix™-HepB/ Hiberix™ with respect to the immunogenicity of all common antigens (anti-PRP, anti-Diphtheria, anti-Tetanus, anti-BP, and anti-HBs).

Criteria to achieve the co-primary objectives:

1. Immunogenicity of SBA-MenA and SBA-MenC: lower limit of the 95% CI for the percentage of subjects with SBA-MenA titers (respectively SBA-MenC titers) ≥ 1:8 is greater than 80%.
2. Non-inferiority for all common antigens: upper limit of the 95% CI for the group difference in terms of the percentage of subjects with anti-PRP concentration ≥ 1 µg/ml (respectively, anti-diphtheria concentration ≥ 0.1 IU/ml, anti-tetanus concentration ≥ 0.1 IU/ml, anti-HBs concentration ≥ 10 mIU/ml) is below 10% and upper limit of the 95% CI for the group GMC ratio in anti-BP is below 1.5.

In order to cope with the multiplicity of the objectives, the criteria of the objectives will be assessed sequentially and will lead to a conclusion only if all previous primary criteria have been met (refer to Section 10.6.2). **Table 10-1** and **Table 10-2** present the order in which the criteria will be assessed (see Rank), the power to meet each criterion and the power to meet all previous criteria (see cumulative power). For instance, the power to meet the first primary objective and non-inferiority for anti-PRP will be 78.6% assuming that the 2 vaccine groups are identical with respect to the primary endpoints.

Table 10-1: *Power for criterion 1 (one-sided test on one proportion, alpha=0.025, N=110/group, power for H_0: p = 80% under the alternative hypothesis H_a: p = 94%, power calculated using PASS 2000)*

Rank	Endpoint	Reference	Individual Power	Cumulative power
1	SBA Men C = 1:8	94%	99.4%	99.4%
2	SBA Men A = 1:8	94%	99.4%	98.8%

Reference: MenAC-Hib-001

Table 10-2: *Power for criterion 2 (For all antigens except anti-BP: one-sided test of equivalence of two proportions, alpha=0.025, N=110/group, power calculated using PASS 2000, assuming identical seroprotection rates for the two groups; for anti-BP: one-sided t-test of equivalence of two means, alpha = 0.025, N = 110/group, power calculated using nQuery 4 and assuming identical GMCs for the two groups.)*

Rank	Endpoint	Reference	Individual Power	Cumulative power
3	Anti-PRP = 1 µg/ml	94%	79.8%	78.6%
4	Anti-tetanus = 0.1 IU/ml	98%	97.9%	76.5%
5	Anti-Diphtheria = 0.1 IU/ml by ELISA and, if negative, ≥ 0.016 IU/ml by Vero-cell	96%	89.1%	65.6%
6	GMC of anti-BP	0.298*	99.0%	64.9%
7	Anti-HBs = 10 mIU/ml	95%	84.4%	49.0%

*standard deviation of log10 (concentration)
References: MenAC-Hib-001 for % of anti-PRP, Hib-065 for % of anti-tetanus, anti-diphtheria (by ELISA only) and anti-HBs, DTPw-HBV-033 for s.d. of log10 (concentration) for anti-BP.

It is acknowledged that the power to meet all primary objectives is low (at worst 49.0%). However, considering this is a Phase II feasibility study, the low power is deemed acceptable.

10.4 Study cohorts to be evaluated

A total of 6 cohorts are defined for the purpose of analysis.

- Three cohorts that will be used for the analysis of the primary vaccination course:
  - the Total vaccinated cohort
  - the ATP cohort for safety
  - the ATP cohort for immunogenicity (used for the analysis of the co-primary objectives of immunogenicity)

- Three cohorts that will be used for the analysis of the booster phase:
  - the Total vaccinated cohort

- the ATP cohort for safety
- the ATP cohort for immune memory

The Total vaccinated cohort (Primary vaccination)

The Total vaccinated cohort will include all subjects vaccinated in the primary phase of the study. For the Total analysis of safety, this will include all subjects vaccinated in the primary phase of the study with at least one vaccine administration documented in the primary phase. For the Total analysis of immunogenicity / efficacy, this will include subjects vaccinated in the primary phase of the study for whom data concerning immunogenicity/efficacy endpoint measures are available. The Total vaccinated cohort analysis will be performed per treatment actually administered.

According to protocol (ATP) cohort for safety (Primary vaccination)

The According to protocol (ATP) cohort for safety will include all subjects:

- who have received at least one dose of study vaccine/control according to their random assignment,
- with sufficient data to perform an analysis of safety,
- for whom the administration route of study vaccine/control is known and correct,
- who have not received a vaccine not specified or forbidden in the protocol,
- who did not receive a replacement vaccine,

during the primary vaccination course.

According to protocol (ATP) cohort for immunogenicity (Primary vaccination)

The According to protocol (ATP) cohort for immunogenicity will include all evaluable subjects (i.e. those meeting all eligibility criteria, complying with the procedures defined in the protocol, and with no elimination criteria during the study) from the ATP cohort for safety for whom assay results are available for antibodies against at least one study vaccine antigen component at Visit 4.

The Total vaccinated cohort (Booster vaccination)

The Total vaccinated cohort will include all subjects vaccinated in the booster phase of the study. For the Total analysis of safety, this will include all subjects vaccinated in the booster phase of the study with at least one vaccine administration documented in the booster phase. For the Total analysis of immunogenicity/efficacity, this will include subjects vaccinated in the booster phase of the study for whom data concerning immunogenicity / efficacy endpoint measures are available. The Total vaccinated cohort analysis will be performed per treatment actually administered.

According To Protocol (ATP) cohort for safety (Booster vaccination)

The ATP cohort for safety will include all subjects from the ATP cohort (Primary vaccination) for safety

- who have received the 3 doses in the primary vaccination course,
- who have received the booster dose,
- with documented safety follow-up,
- for whom administration route of Mencevax™ AC is known,
- who have not received a vaccine not specified or forbidden in the protocol.

This cohort will also be used for the analysis of persistence of the immune response.

According To Protocol (ATP) cohort for immune memory

The ATP cohort for immune memory will include all evaluable subjects (i.e., those meeting all eligibility criteria, complying with the procedures defined in the protocol, with no elimination criteria during the study) from the Booster ATP cohort for safety for whom assay results are available for antibodies against at least one study vaccine antigen component at Visit 7.

10.5 Derived and transformed data

Immunogenicity

For a given subject cohort and the analysis of a given measurement, missing or unevaluable measurements will not be replaced. Therefore an analysis will exclude subjects with missing or unevaluable measurements.

- The cut-off value is defined by the laboratory before the analysis and is described in Section 5.5.2.
- A seronegative subject is a subject whose titer or concentration is below the cut-off value.
- A seropositive subject is a subject whose titer or concentration is greater than or equal to the cut-off value.
- Antibody concentrations or titers below the cut-off of the assay will be given an arbitrary value of half the cut-off for the purpose of GMC or GMT calculation.

Other

Subjects who missed reporting symptoms (solicited or unsolicited or concomitant medications) will be treated as subjects without symptoms (solicited or unsolicited or concomitant medications, respectively).

10.6 Final analyses for the primary vaccination course

10.6.1 Analysis of demographics/baseline characteristics

Demographic characteristics (age in weeks, gender, race) of each study cohort will be tabulated.

The mean age (plus range and standard deviation) by gender of the enrolled subjects, as a whole, and per group, will be calculated.

The distribution of subjects enrolled among the study sites will be tabulated as a whole and per group.

10.6.2 Analysis of Immunogenicity

The analysis of immunogenicity will be based on the ATP cohort for immunogenicity. If, for any vaccine group, the percentage of enrolled subjects with serological results excluded from this ATP cohort is more than 5%, a second analysis based on the Total vaccinated cohort will be performed to complement the ATP analysis.

Analysis of immunogenicity will be as follows:

Descriptive analysis

For each treatment group, one month after the third dose of the primary vaccination course, and in the mother prior to her child's vaccination, for each serological assay done:

- Geometric Mean antibody Concentrations/Titers (GMCs/GMTs) with 95% confidence intervals (CIs) will be tabulated for each antibody. Calculation of the GMCs/GMTs is performed by taking the anti-log of the mean of the log concentration/titer transformations.

- Seropositivity / seroprotection rates with exact 95% CIs will be calculated.

- Antibody concentrations/titers post-vaccination will also be investigated using reverse cumulative curves for each antigen and serogroup.

Inferential analysis

The inferential analysis will be done sequentially.

1. If the lower limit of the 95% CI for the percentage of subjects with SBA-MenC is above 80%, one will conclude that Tritanrix™-HepB/Hib-MenAC is immunogenic for MenC.

2. If, in addition, the lower limit of the 95% CI of subjects with SBA-MenA is above 80%, one will conclude that Tritanrix™-HepB/Hib-MenAC is immunogenic for MenA.

3. If, in addition, the upper limit of the 95% CI for the group difference for anti-PRP is below 10% one will conclude non-inferiority with respect to anti-PRP.

4. If, in addition, the upper limit of the 95% CI for the group difference for anti-Tetanus is below 10% one will conclude non-inferiority with respect to anti-Tetanus.

5. If, in addition, the upper limit of the 95% CI for the group difference for anti-Diphtheria is below 10% one will conclude non-inferiority with respect to anti-Diphtheria.

6. If, in addition, the upper limit of the 95% CI for the anti-BP GMC ratio (control vaccine / new vaccine) is below 1.5 one will conclude non-inferiority with respect to anti-BP.

7. If, in addition, the upper limit of the 95% CI for the group difference for anti-HBs is below 10% one will conclude non-inferiority with respect to anti-HBs.

10.6.3 Analysis of Safety

The primary analysis will be based on the Total vaccinated cohort. If the percentage of enrolled subjects excluded from the ATP cohort for safety is more than 5%, a second analysis based on this ATP cohort will be performed to complement the Total vaccinated cohort analysis.

The percentage of subjects with at least one local adverse event (solicited and unsolicited), with at least one general adverse event (solicited and unsolicited) and with any adverse event during the 4-day solicited follow-up period will be tabulated with exact

95% CI after each vaccine dose and overall. The percentage of doses followed by at least one local adverse event (solicited and unsolicited), by at least one general adverse event (solicited and unsolicited) and by any adverse event will be tabulated, overall vaccination course, with exact 95% CI.

The percentage of subjects reporting each individual solicited local and general adverse event during the 4-day solicited follow-up period will be tabulated with exact 95% CI. The percentage of doses followed by each individual solicited local and general adverse event will be tabulated, overall vaccination course, with exact 95% CI.

The same tabulation will be performed for grade 3 adverse events and for adverse events with relationship to vaccination.

The proportion of subjects with at least one report of unsolicited adverse event classified by the Medical Dictionary for Regulatory Activities (MedDRA), and reported up to 30 days after vaccination will be tabulated with exact 95% CI. The same tabulation will be performed for grade 3 unsolicited adverse events and for unsolicited adverse events with a relationship to vaccination.

Serious adverse events and withdrawal due to adverse event(s) will be described in detail.

10.7 Final Analyses for the booster vaccination

10.7.1 Analysis of immunogenicity

The analysis of antibody persistence and the analysis of immune memory will be respectively based on the ATP cohort for safety and the ATP cohort for immune memory. If, for any vaccine group, the percentage of enrolled subjects with serological results excluded from an ATP cohort is more than 5%, a second analysis based on the Total vaccinated cohort will be performed to complement the ATP analysis.

The analysis of immunogenicity will be as follows:

Analysis of antibody persistence

For each treatment group, one month after the third dose of the primary vaccination course and just before the administration of one fifth of a dose of Mencevax™ AC:

- Geometric Mean antibody Concentrations/Titers (GMCs/GMTs) with 95% confidence intervals (CIs) will be tabulated for each antibody. Calculation of the

GMCs/GMTs is performed by taking the anti-log of the mean of the log concentration/titer transformations.

- Seropositivity / seroprotection rates with exact 95% CIs will be calculated.
- Antibody concentrations/titers pre-booster will also be investigated using reverse cumulative curves for each antigen and serotype.

Analysis of immune memory

For each treatment group, just before and one month after the administration of one fifth of a dose of Mencevax™ AC:

- Geometric Mean antibody Concentrations/Titers (GMCs/GMTs) with 95% confidence intervals (CIs) will be tabulated for each antibody. Calculation of the GMCs/GMTs is performed by taking the anti-log of the mean of the log concentration/titer transformations.
- Seropositivity / seroprotection rates with exact 95% CIs will be calculated.
- Antibody concentrations/titers post-booster will also be investigated using reverse cumulative curves for each antigen and serotype.

The ratio of the GMCs/GMTs between the Tritanrix™-HepB/Hib-MenAC group and the Tritanrix™-HepB/Hiberix™ group one month after the administration of one fifth of a dose of Mencevax™ AC, will be computed with its 95% CI. The ratio and its 95% CI will be computed using an ANOVA model on the log-transformed concentration/titer with group effect as regressor.

10.7.2 Analysis of safety

All analyses will be based on the Total vaccinated cohort. If the percentage of enrolled subjects excluded from the ATP cohort for safety is more than 5%, a second analysis based on this ATP cohort will be performed to complement the Total vaccinated cohort analysis.

The percentage of subjects with at least one local adverse event (solicited and unsolicited), with at least one general adverse event (solicited and unsolicited) and with any adverse event during the 4-day solicited follow-up period will be tabulated with exact 95% CI after each vaccine dose and overall.

The percentage of subjects reporting each individual solicited local and general adverse event during the 4-day solicited follow-up period will be tabulated with exact 95% CI.

The same tabulation will be performed for grade 3 adverse events and for adverse events with relationship to vaccination.

The proportion of subjects with at least one report of unsolicited adverse event classified by the Medical Dictionary for Regulatory Activities (MedDRA), and reported up to 30 days after vaccination will be tabulated with exact 95% CI. The same tabulation will be performed for grade 3 unsolicited adverse events and for unsolicited adverse events with a relationship to vaccination.

Serious adverse events and withdrawal due to adverse event(s) will be described in detail.

10.8 Reporting of final analyses

A final analysis for the primary vaccination course will be carried out as soon as all data up to Visit 4 have been collected in order to analyse the immunogenicity endpoints (including the co-primary endpoints) and the analysis of safety/reactogenicity for the primary vaccination course (refer to Section 10.6 for details about this analysis). A report based on this analysis will be written and will be made available to the Principal Investigator and the sponsors involved in this study. The access to the individual treatment code will be limited to the statistician in charge of this analysis.

A final analysis for the booster vaccination will be carried out based on all data collected up to one month after the administration of one fifth of a dose of Mencevax™ AC. An annex report based on the final analysis for the booster vaccination will be provided.

Serious adverse events reported during the extended follow-up period (i.e. from visit 7 up to 24 months of age) will be reported in a separate annex report.

(Amendment 7: 12 April 2006)

10.9 Planned interim analysis

No interim analysis is planned.

11 Administrative Matters

To comply with Good Clinical Practice important administrative obligations relating to investigator responsibilities, monitoring, archiving data, audits, confidentiality and publications must be fulfilled. See Appendix B for details.

References

1 Kirsch EA, Barton RP, Kitchen L, Giroir BP. Pathophysiology, treatment and outcome of meningococcemia: a review and recent experience. Pediatr Infect Dis 1996;15:967–979.

2 Anderson MS, Glodé MP, Smith AL. Meningococcal disease. In Feigin RD, Cherry JD, editors. Textbook of Pediatric Infectious Diseases. Philadelphia. WB Saunders. 1998:1143–1156.

3 Anonymous. Serogroup B meningococcal disease: Oregon, 1994. MMWR 1995;44:121–124.

4 Whalen, CM, Hockin JC, Ryan A et al. The changing epidemiology of invasive meningococcal disease in Canada, 1985 through 1992: emergence of a virulent clone of Neisseria meningitidis. JAMA. 1995;273:390–394.

5 Connolly M, Noah N. Is group C meningococcal disease increasing in Europe? A report of surveillance of meningococcal infection in Europe 1993–6. Epidemiol Infect 1999;122(1):41–49.

6 Lingappa J, Rosenstein N, Zell E, Stat M, Perkins B, Schuchat A, CDC - Surveillance for meningococcal disease and strategies for use of conjugate meningococcal vaccines in the United States, IDSA 1999, abstract 644.

7 Greenwood BM. Meningococcal meningitis in Africa.Trans R SOC Trop Med Hyg 1999, 93: 341–353.

8 Black S, Shinefield HR, Fireman B, Hiatt R, Polen M, Vittinghoff E, et al. Efficacy in infancy of oligosaccharide conjugate *Haemophilus influenzae* type b (HbOC) vaccine in a United States population of 61080 children. Pediatric Infectious Disease Journal 1991;10: 97–104.

9 Booy R, Moxon ER, MacFarlane JA, Mayon-White RT, Slack MP. Efficacy of *Haemophilus influenzae* type b conjugate vaccines in the Oxford region. Lancet 1992;340: 847.

10 Eskola J, Peltola H, Käythty H, Takala AK, Mäkelä. Finnish efficacy trials with *Haemophilus influenzae* type b vaccines. Journal Infectious Diseases 1992;165 (Suppl 1): S137–8.

11 Zepp F, Schmitt HJ, Kaufhold A, Schuind A, Knuf M, Habermehl P, Meyer C, Bogaerts H, Slaoui M, Clemens R. Evidence for induction of polysaccharide specific B-cell-memory in the 1st year of life: plain Haemophilus influenzae type b- PRP (Hib) boosters children primed with tetanus-conjugate Hib-DTPa-HBV combined vaccine. European Journal of Pediatrics 1997;156:18–24.

12 Ramsey ME, Andrews N, Kaczmarski AB, Miller E. Efficacy of meningococcal serogroup C conjugate vaccine in teenagers and toddlers in England. The Lancet 2001;357:195–196.

13 Miller E, Salisbury D, Ramsay M. Planning, registration, and implementation of an immunization campaign against meningococcal serogroup C disease in the UK: a success story. Vaccine 2002;20:S58-S67

14 Borrow R, Kaczmarski E, Andrews N, Gungabissoon U, Campbell H, Ramsay M, Miller E. Update on meningococcal C conjugate vaccination programme in England and Wales: vaccine failures, vaccine efficacy, herd immunity and characterization of *N. meningitidis* isolates. ICAAC 2001, abstract UL-16

15 Leach A, Twumasi PA, Kumah S, Banya WS, Jaffar S, Forrest BD, Granoff DM, LiButti DE, Carlone GM, Pais LB, Broome CV, Greenwood BM. Induction of immunologic memory in Gambian children by vaccination in infancy with a group A plus group C meningococcal polysaccharide-protein conjugate vaccine. Journal Infectious Diseases 1997;175:200-4.

16 Campagne G, Garba A, Fabre P, Schuchat A, Ryall R, Boulanger D, Bybel M, Carlone G, Briantais P, Ivanoff B, Xerri B, Chippaux JP. Safety and immunogenicity of three doses of a Neisseria meningitidis A + C diphtheria conjugate vaccine in infants from Niger. Pediatric Infectious Disease Journal 2000;19(2):144-50.

17 Richmond P, Borrow R, Miller E, Clarck S, Sadler F, Fox A, Begg N, Morris R, Cartwright K. Meningococcal serogroup C conjugate vaccine is immunogenic in infancy and primes for memory. Journal Infectious Diseases 1999; 179: 1569-72.

18 Borrow R, Fox AJ, Richmond PC, Clarck S, Sadler F, Findlow J, Morris R, Begg NT, Cartwright K. Induction of immunological memory in UK infants by a meningococcal A/C conjugate vaccine. Epidemiol. Infect 2000; 124: 427-432.

19 Binka FN, Ngom P, Phillips JF, Adazu K, MacLeod BB. Assessing population dynamics in a rural African society: the Navrongo Demographic Surveillance System. J Biosoc Sci 1999;31:375-391.

20 Gagneux SP, Hodgson A, Smith TA, Wirth T, Ehrhard I, Morelli G, Genton B, Binka FN, Achtman M, Pluschke G. Prospective study of a serogroup X *Neisseria meningitidis* outbreak in northern Ghana. J Infect Dis 2002a;185(5):618-626.

21 Gagneux S, Wirth T, Hodgson A, Ehrhard I, Morelli G, Kriz P, Genton B, Smith T, Binka F, Pluschke G, Achtman M. Clonal groupings in serogroup X *Neisseria meningitidis*. Emerg Infect Dis 2002b;8(5):462-466.

22 Hodgson A, Smith T, Gagneux S, Adjuik M, Pluschke G, Mensah NK, Binka F, Genton B. Risk factors for meningococcal meningitis in northern Ghana. Trans R Soc Trop Med Hyg 2001a; 95(5):477-480.

23 Hodgson A**,** Smith T, Gagneux S, Akumah I, Adjuik M, Pluschke G, Binka F, Genton B. Survival and sequelae of meningococcal meningitis in Ghana. Int J Epidemiol 2001b;30(6):1440-1446.

24 Gagneux S, Hodgson A, Ehrhard I, Morelli G, Genton B, Smith T, Tanner M, Binka F, Achtman M, Pluschke G. Microheterogeneity of serogroup A (subgroup III) *Neisseria*

meningitidis during an outbreak in northern Ghana. Trop Med Int Health 2000;5(4):280-287.

25 Nyarko, P., Wontuo, P., Wak, G., Chirawurah, D., Welaga, P., Nchor, S., Hodgson, A., and Binka, F. The Navrongo Demographic Surveillance System; 2001 Report to the Rockefeller Foundation. Community Health and Family Planning Project Documentation Note Number 45

26 Enos K. Cerebrospinal meningitis in northern Ghana: the experience of the War Memorial Hospital, Navrongo [Report]. Ghana: Ministry of Health. 1997.

27 Maslanka SE, Gheesling LL, Libutti D.E et al. Standardization and a multilaboratory comparison of Neisseria meningitidis serogroup A and C serum bactericidal assays. Clin. Diagn. Lab. Immunol. 1997;4(2):156–167.

28 Borrow R, Andrews N, Goldblatt D, Miller E. Serological basis for use of meningococcal serogroup C conjugate vaccines in the United Kingdom: reevaluation of correlates of protection. Infect Immun 2001;69:1568–1573.

29 Andrews N, Borrow R, Miller E. Validation of serological correlate of protection for meningococcal C conjugate vaccine by using efficacy estimates from post licensure surveillance in England. Clin Diagn Lab Immunol 2003;10:780-786.

30 Gheesling LL, Carlone GM, Pais LB et al. Multicenter comparison of Neisseria meningitidis serogroup C anti-capsular polysaccharide antibody levels measured by a standardized Enzyme-Linked Immunosorbent Assay. J Clin Microbiol 1994;32:1475–1482.

31 Camargo ME, et al. Immunoenzymatic assay of anti-diphtheric toxin antibodies in human serum. J Clin Microbiol 1984; 20 (4): 772–774.

32 Melville-Smith ME, et al. A comparison of enzyme-linked immunosorbent assay (ELISA) with the toxin neutralization test in mice as a method for the estimation of tetanus antitoxin in human sera. J Biol Standard 1983; 11 (2): 137–144.

33 Miyamura K, Nisho, S Ito A, Murata R, Kono R. Micro cell culture method for determination of diphtheria toxin and antitoxin concentrations using VERO cells: I. Studies on factors affecting the toxin and antitoxin titration. J Biol Stand 1974;2:189-201.

34 Miyamura K, Nisho ,S Ito A, Murata R, Kono R. Micro cell culture method for determination of diphtheria toxin and antitoxin concentrations using VERO cells: ***II.*** Comparison with rabbit skin method and practical application for seroepidemiological studies. J. Biol Stand 1974;2:203-209.

35 Bergson PS, Singer SA, Kaplan AR. Intramuscular Injections in Children. Pediatrics 1982; 70 (6): 944

Appendix A: World Medical Association Declaration of Helsinki

Recommendations guiding physicians
in biomedical research involving human subjects

Adopted by the 18th World Medical Assembly
Helsinki, Finland, June 1964

and amended by the
29th World Medical Assembly
Tokyo, Japan, October 1975
35th World Medical Assembly
Venice, Italy, October 1983
41st World Medical Assembly
Hong Kong, September 1989
and the
48th General Assembly
Somerset West, Republic of South Africa, October 1996

INTRODUCTION

It is the mission of the physician to safeguard the health of the people. His or her knowledge and conscience are dedicated to the fulfilment of this mission.

The Declaration of Geneva of the World Medical Association binds the physician with the words, "The health of my patient will be my first consideration," and the International Code of Medical Ethics declares that, "A physician shall act only in the patient's interest when providing medical care which might have the effect of weakening the physical and mental condition of the patient."

The purpose of biomedical research involving human subjects must be to improve diagnostic, therapeutic and prophylactic procedures and the understanding of the etiology and pathogenesis of disease.

In current medical practice most diagnostic, therapeutic or prophylactic procedures involve hazards. This applies especially to biomedical research.

Medical progress is based on research which ultimately must rest in part on experimentation involving human subjects.

In the field of biomedical research a fundamental distinction must be recognized between medical research in which the aim is essentially diagnostic or therapeutic for a patient, and medical research, the essential object of which is purely scientific and without implying direct diagnostic or therapeutic value to the person subjected to the research.

Special caution must be exercised in the conduct of research which may affect the environment, and the welfare of animals used for research must be respected.

Because it is essential that the results of laboratory experiments be applied to human beings to further scientific knowledge and to help suffering humanity, the World Medical Association has prepared the following recommendations as a guide to every physician in biomedical research involving human subjects. They should be kept under review in the future. It must be stressed that the standards as drafted are only a guide to physicians all over the world. Physicians are not relieved from criminal, civil and ethical responsibilities under the laws of their own countries.

I. BASIC PRINCIPLES

1. Biomedical research involving human subjects must conform to generally accepted scientific principles and should be based on adequately performed laboratory and animal experimentation and on a thorough knowledge of the scientific literature.

2. The design and performance of each experimental procedure involving human subjects should be clearly formulated in an experimental protocol which should be transmitted for consideration, comment and guidance to a specially appointed committee independent of the investigator and the sponsor provided that this independent committee is in conformity with the laws and regulations of the country in which the research experiment is performed.

3. Biomedical research involving human subjects should be conducted only by scientifically qualified persons and under the supervision of a clinically competent medical person. The responsibility for the human subject must always rest with a medically qualified person and never rest on the subject of research, even though the subject has given his or her consent.

4. Biomedical research involving human subjects cannot legitimately be carried out unless the importance of the objective is in proportion to the inherent risk to the subject.

5. Every biomedical research project involving human subjects should be preceded by careful assessment of predictable risks in comparison with foreseeable benefits to the

subject or to others. Concern for the interests of the subject must always prevail over the interests of science and society.

6. The right of the research subject to safeguard his or her integrity must always be respected. Every precaution should be taken to respect the privacy of the subject and to minimize the impact of the study on the subject's physical and mental integrity and on the personality of the subject.

7. Physicians should abstain from engaging in research projects involving human subjects unless they are satisfied that the hazards involved are believed to be predictable. Physicians should cease any investigation if the hazards are found to outweigh the potential benefits.

8. In publication of the results of his or her research, the physician is obliged to preserve the accuracy of the results. Reports of experimentation not in accordance with the principles laid down in this Declaration should not be accepted for publication.

9. In any research on human beings, each potential subject must be adequately informed of the aims, methods, anticipated benefits and potential hazards of the study and the discomfort it may entail. He or she should be informed that he or she is at liberty to abstain from participation in the study and that he or she is free to withdraw his or her consent to participation at any time. The physician should then obtain the subject's freely-given informed consent, preferably in writing.

10. When obtaining informed consent for the research project the physician should be particularly cautious if the subject is in a dependent relationship to him or her or may consent under duress. In that case the informed consent should be obtained by a physician who is not engaged in the investigation and who is completely independent of this official relationship.

11. In case of legal incompetence, informed consent should be obtained from the legal guardian in accordance with national legislation. Where physical or mental incapacity makes it impossible to obtain informed consent, or when the subject is a minor, permission from the responsible relative replaces that of the subject in accordance with national legislation.

    Whenever the minor child is in fact able to give a consent, the minor's consent must be obtained in addition to the consent of the minor's legal guardian.

12. The research protocol should always contain a statement of the ethical considerations involved and should indicate that the principles enunciated in the present Declaration are complied with.

II. MEDICAL RESEARCH COMBINED WITH PROFESSIONAL CARE (Clinical research)

1. In the treatment of the sick person, the physician must be free to use a new diagnostic and therapeutic measure, if in his or her judgement it offers hope of saving life, re-establishing health or alleviating suffering.

2. The potential benefits, hazards and discomfort of a new method should be weighed against the advantages of the best current diagnostic and therapeutic methods.

3. In any medical study, every patient - including those of a control group, if any - should be assured of the best proven diagnostic and therapeutic method. This does not exclude the use of inert placebo in studies where no proven diagnostic or therapeutic method exists.

4. The refusal of the patient to participate in a study must never interfere with the physician–patient relationship.

5. If the physician considers it essential not to obtain informed consent, the specific reasons for this proposal should be stated in the experimental protocol for transmission to the independent committee (I, 2).

6. The Physician can combine medical research with professional care, the objective being the acquisition of new medical knowledge, only to the extent that medical research is justified by its potential diagnostic or therapeutic value for the patient.

III. NON-THERAPEUTIC BIOMEDICAL RESEARCH INVOLVING HUMAN SUBJECTS (Non-clinical biomedical research)

1. In the purely scientific application of medical research carried out on a human being, it is the duty of the physician to remain the protector of the life and health of that person on whom biomedical research is being carried out.

2. The subjects should be volunteers - either healthy persons or patients for whom the experimental design is not related to the patient's illness.

3. The investigator or the investigating team should discontinue the research if in his/her or their judgement it may, if continued, be harmful to the individual.

4. In research on man, the interest of science and society should never take precedence over considerations related to the well being of the subject.

Appendix B: Administrative Matters

I. Responsibilities of the Investigator

- To ensure that he/she has sufficient time to conduct and complete the study and has adequate staff and appropriate facilities and equipment which are available for the duration of the study and to ensure that other studies do not divert essential subjects or facilities away from the study at hand.

- To submit an up-to-date curriculum vitae and other credentials (e.g., medical license number in the United States) to GSK Biologicals and—where required—to relevant authorities.

- To acquire the normal ranges for laboratory tests performed locally and, if required by local regulations, obtain the Laboratory License or Certification.

- To ensure that no clinical samples (including serum samples) are retained on site or elsewhere without the approval of GSK Biologicals and the express written informed consent of the subject and/or the subject's legally authorized representative.

- To perform no other biological assays at the investigator site except those described in the protocol or its amendment(s).

- To prepare and maintain adequate case histories designed to record observations and other data pertinent to the study.

- To conduct the study in compliance with the protocol and appendices.

- To co-operate with a representative of GSK Biologicals in the monitoring process of the study and in resolution of queries about the data.

II. Protocol Amendments and Modifications

- No changes to the study protocol will be allowed unless discussed in detail with GSK Biologicals' Clinical Development Manager/Medical Monitor and filed as an amendment/modification to this protocol.

- Any amendment/modification to the protocol will be adhered to by the participating center(s) and will apply to all subjects. Written IRB/IEC approval of protocol amendments is required prior to implementation; modifications are submitted to IRBs/IECs for information only.

III. Sponsor's Termination of Study

GSK Biologicals reserves the right to discontinue the clinical study at any time for medical or administrative reasons. When feasible, a 30-day written notification will be tendered.

IV. Case Report Form Instructions

Prior to screening the first potential participant, the investigator will provide the Site Monitor with a list (Site Staff Signature Sheet) showing the signature and hand-written initials of all individuals authorized to make or change entries on CRFs (already defined). If the authorized individuals should change during the study, the investigator is to inform GSK Biologicals of the specific change(s).

CRFs (and subject diary cards, if applicable), will be supplied by GSK Biologicals for recording all data. It is the responsibility of the investigator or co-investigator to ensure that CRFs (and subject diary cards) are legible and completely filled in with a black ink fountain or ballpoint pen.

Errors must be corrected by drawing a single line through the incorrect entry and writing in the new value/data positioned as close to the original as possible. The correction must then be initialled, dated and justified, where necessary, by the authorized individual making the change. The original entry must not be obliterated, overwritten or erased when a correction is made.

When a subject completes a visit, it is anticipated that relevant sections of the CRF will be completed by the investigator (or designated staff as documented in the Site Staff Signature Sheet) as soon as possible after the last data becoming available. Similarly, when a subject completes a study, it is anticipated that all relevant CRF pages will be completed promptly after the last data becoming available. This also applies to forms for potential study participants who were screened but not randomized to a study group.

As soon as the subject has completed/withdrawn from the study and the CRF is completed, the principal investigator or designated physician(s) under his/her supervision will sign the study conclusion pages of the CRF to confirm that they have reviewed the data and that the data are complete and accurate. In all cases the investigator remains accountable for the study data collected.

An original (top copy) CRF or log sheets must be submitted for all subjects who have undergone protocol specific procedures, whether or not the subject completed the study.

While completed CRFs are reviewed by a GSK Biologicals professional monitor at the study site, errors detected by subsequent in-house CRF review may necessitate

clarification or correction of errors with documentation and approval by the investigator or appropriately qualified staff as documented on the Site Staff Signature Sheet. In all cases, the investigator remains accountable for the study data. Wherever possible the investigator should assist in the clarification or correction of errors detected after study finalization promptly after being brought to the attention of the investigator (preferably within 48 hours).

Any questions or comments related to the CRF should be directed to the assigned Site Monitor.

V. Monitoring by GSK Biologicals

Monitoring visits by a professional representative of GSK Biologicals will be scheduled to take place as close as possible to entry of the first subject, during the study at appropriate intervals and after the last subject has completed the study. It is anticipated that monitoring visits will occur at a frequency defined before study start.

These visits are for the purpose of confirming that GSK Biologicals' sponsored studies are being conducted in compliance with the relevant Good Clinical Practice regulations/ guidelines, verifying adherence to the protocol and the completeness and accuracy of data entered on the CRF pages and Vaccine Inventory Forms. The monitor will verify CRF entries by comparing them with the source data/documents that will be made available by the investigator for this purpose. Data to be recorded directly into the CRF pages will be specified in writing preferably in the source documentation agreement form that is contained in both the monitor's and investigator's study file. The investigator must ensure provision of reasonable time, space and adequate qualified personnel for monitoring visits.

Monitoring by WHO

Independent WHO monitors will visit the study site to monitor procedures/data.

VI. Archiving of Data

A study report will be written after the statistical analysis. This report, including all individual data, will be given to the sponsors of the study and to the principal investigator.

Following closure of the study, the investigator must maintain all site study records in a safe and secure location. The records must be maintained to allow easy and timely retrieval, when needed (e.g., audit or inspection), and, whenever feasible, to allow any subsequent review of data in conjunction with assessment of the facility, supporting systems, and staff. Where permitted by applicable laws/regulations or institutional policy, some or all of these records can be maintained in a validated format other than

hard copy (e.g., microfiche, scanned, electronic for studies with an eCRF, for example); however, caution needs to be exercised before such action is taken. The investigator must assure that all reproductions are legible and are a true and accurate copy of the original, and meet accessibility and retrieval standards, including re-generating a hard copy, if required. Furthermore, the investigator must ensure there is an acceptable back-up of these reproductions and that an acceptable quality control process exists for making these reproductions.

GSK will inform the investigator/ institution of the time period for retaining these records to comply with all applicable regulatory requirements. However, the investigator/ institution should seek the written approval of the sponsors before proceeding with the disposal of these records. The minimum retention time will meet the strictest standard applicable to that site for the study, as dictated by ICH GCP E6 Section 4.9, any institutional requirements or applicable laws or regulations, or GSK Biologicals standards/procedures; otherwise, the minimum retention period will default to 15 years.

The investigator/ institution must notify GSK Biologicals of any changes in the archival arrangements, including, but not limited to, the following: archival at an off-site facility, transfer of ownership of the records in the event the investigator leaves the site.

VII. Audits

For the purpose of compliance with Good Clinical Practice and Regulatory Agency Guidelines it may be necessary for GSK Biologicals or a Drug Regulatory Agency to conduct a site audit. This may occur at any time from start to after conclusion of the study.

When an investigator signs the protocol, he/she agrees to permit drug regulatory agencies and GSK Biologicals audits, providing direct access to source data/ documents. Furthermore, if an investigator refuses an inspection, his/her data will not be accepted in support of a New Drug Registration and/or Application, Biologics Licensing Application.

GSK Biologicals has made and is making a substantial investment in clinical studies. Having the highest quality data and studies are essential aspects of vaccine development. GSK Biologicals has a Regulatory Compliance staff who audit investigational sites. Regulatory Compliance assesses the quality of data with regard to accuracy, adequacy and consistency. In addition, Regulatory Compliance assures that GSK Biologicals sponsored studies are in accordance with GCP and that relevant regulations/guidelines are being followed.

To accomplish these functions, Regulatory Compliance selects investigational sites to audit. These audits usually take 1 to 2 days. The GSK Biologicals' audits entail review of

source documents supporting the adequacy and accuracy of CRFs, review of documentation required to be maintained, and checks on vaccine accountability. The GSK Biologicals' audit therefore helps prepare an investigator for a possible regulatory agency inspection as well as assuring GSK Biologicals of the validity of the database across investigational sites.

The Inspector will be especially interested in the following items:

- Log of visits from the sponsors' representatives
- IRB/IEC approval
- Vaccine accountability
- Approved study protocol and amendments
- Informed consent of the subjects (written consent [or witnessed oral if applicable])
- Medical records and other source documents supportive of CRF data
- Reports to the IRB/IEC and the sponsor
- Record retention

GSK Biologicals will gladly help investigators prepare for an inspection.

VIII. Confidentiality and Publication

You, the investigator, agree that all information communicated to you by GSK Biologicals is the exclusive property of GSK Biologicals.You agree to keep such information strictly confidential and to restrict access to such information to the minimum number of persons connected with the work performed under this protocol on a need-to-know basis and you will ensure that such persons shall be made aware that the information is confidential and shall be bound by confidentiality obligations at least as strict as those contained herein. You will be responsible for any breach of the confidentiality by any of those persons to whom such information has been furnished. You and any such person agree that said information shall not be disclosed, either orally or in written form, by you or such person to any third party without the prior written consent of GSK Biologicals. You shall communicate the results of the work promptly to GSK Biologicals. Such results shall be the property of GSK Biologicals and shall be subject to the confidentiality obligations contained herein except that you shall be authorised to publish the results pursuant to the provisions below.

The results of the study covered by this protocol shall be the subject of a joint publication by the sponsors and you within six (6) months from the conclusion of the study and availability of a final study report. You and the sponsors will be expected to treat matters of authorship in a proper collaborative spirit, giving credit where it is due and proceeding in a manner that fosters cooperation and communication, but will not do anything in this regard that will jeopardize the issuance of a valid patent.

Besides and after such joint publication, GSK Biologicals agrees that you shall have the right to publish or permit the publication of any information or material relating to or arising out of the work performed under the study protocol after submission to GSK Biologicals at least forty-five (45) days prior to the intended publication provided that if GSK Biologicals shall so request, you will delete any confidential information of GSK Biologicals and/or delay publication for a further sixty (60) days to enable GSK Biologicals to protect its rights in such information or material. Any proposed publication or presentation (e.g. manuscript, abstract or poster) for submission to a journal or scientific meeting, should also be sent to the Clinical Development Manager (CDM) at GSK Biologicals forty-five (45) days prior to submission, together with confirmation that any other author(s) has seen and agreed to the proposed publication/presentation. GSK Biologicals will undertake to comment on such documents within said forty-five (45) days.

GSK Biologicals will be acknowledged in all such publications or presentations by co-authorship or acknowledgement whichever is appropriate.

All rights and interests worldwide in any inventions, know-how or other intellectual or industrial property rights which arise during the course of and/or as a result of the clinical study which is the subject of this protocol or which otherwise arise from the information or materials supplied under this protocol, shall be assigned to, vest in and remain the property of GSK Biologicals.Only GSK Biologicals shall be authorised to utilise the results of the study for regulatory and commercial purposes.

Appendix C: Overview of the Recruitment Plan

The study will be conducted in the Kassena-Nankana district by the Navrongo Health Research Center in Ghana.

A series of meetings inviting regional and district leaders, compound heads and parents is organized by the NHRC, where the aim of the study is explained and questions can be answered.

Mothers delivering at the Navrongo War Memorial Hospital will be informed about the study and asked permission to contact them in the future about potential participation to the trial.

One day before first immunization, trained field workers will perform visits to the villages of potential study participants to invite them to attend the hospital/health center the next day.

On the day of immunization, prior to any study procedure, the parent information sheet and informed consent forms are read and explained to the subject's parents/guardians in the local language and according to the local custom and questions can be answered. Literate parents/guardians are given the information sheet and IC form to read.

When agreement is given by the parent/guardian, the IC is signed or thumbprinted (illiterates) by the parents/guardians of the subject, witnessed by an independent person.

All subjects should be enrolled within a period of 5 months.

Anticipated recruitment rate is approximately 56 subjects per month. Subjects will be recruited when they present themselves at the immunization clinics.

- Enrolment will be terminated when 280 subjects are enrolled.
- The recruitment will be monitored by weekly update through regular contacts with study monitors

Appendix D: Handling of Biological Samples Collected by the Investigator

Instructions for Handling of Serum Samples

When materials are provided by GSK Biologicals, it is mandatory that all clinical samples (including serum samples) be collected and stored using exclusively those materials in the appropriate manner. The use of other materials could result in the exclusion of the subject from analysis. The investigator must ensure that his/her personnel and the laboratory(ies) under his/her supervision comply with this requirement.

1. Collection

The whole blood (by capillary or venous route) should be collected observing appropriate aseptic conditions. It is recommended that Vacutainer® tubes WITH integrated serum separator (e.g. Becton-Dickinson Vacutainer® SST or Corvac® Sherwood Medical) be used to minimize the risk of haemolysis and to avoid blood cell contamination of the serum when transferring to standard serum tubes.

2. Serum separation

These guidelines aim to ensure high quality serum by minimizing the risk of haemolysis, blood cell contamination of the serum or serum adverse cell toxicity at testing.

- For separation of serum using Vacutainer® tubes, the instructions provided by the manufacturer should be followed. Siliconized tubes should never be used (cell toxicity). Often the manufacturer's instruction states that the relative centrifugal acceleration known also as "G" must be "between 1000 and 1300 G" with tubes spinning for ten minutes. Error in calculation of centrifuge speed can occur when laboratory personnel confuse "G" acceleration with "RPM" (revolutions per minute). The speed of centrifugation must be calculated using the "G" rate provided in the manufacturer's instructions and the radius of the centrifuge head. After measuring the radius of the centrifuge machine, a speed/acceleration nomograph must be employed to determine the centrifuge speed in "RPM".

- Following separation, the serum should be aseptically transferred to the appropriate standard tubes using a sterile disposable pipette. The serum should be transferred as gently as possible to avoid blood cell contamination.

- The tube should not be overfilled (max. 3/4 of the total volume) to allow room for expansion upon freezing.

- The tube should be identified by the appropriate label provided by GSK Biologicals (see point 3).

3. Labelling

- The standard labels provided by GSK Biologicals should be used to label each serum sample.

- If necessary, any hand-written additions to the labels should be made using indelible ink.

- The label should be attached to the tube as follows (see diagram):
  - first attach the paper part of the label to the tube
  - then wrap the label around the tube so that the transparent, plastic part of the label overlaps with the label text and bar code and shields them.

This will ensure optimal label attachment.

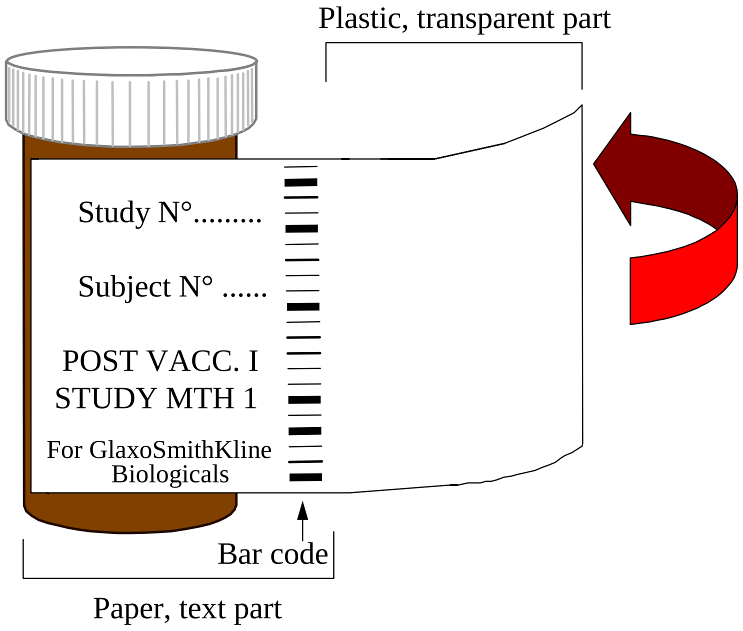

- Labels should not be attached to caps.

4. Sorting and storage

- Tubes should be placed in the GSK Biologicals' carton in numerical order from left to right, starting from the lower left hand corner, beginning with the pre-vaccination samples series, then with the post-vaccination sample series.

- The tubes of serum should be stored in a vertical position at -20°C (alternatively at -70°/80°C is also acceptable) until shipment to GSK Biologicals. Wherever possible, a backup facility for storage of serum samples should be available.

- A standard Serum Listing Form, specifying the samples being shipped for individual subjects at each timepoint, should be prepared for each shipment. A copy of this list should be retained at the study site, while the original should be sealed in a plastic envelope and shipped with the serum samples.

- Once flight details are known, a standard Specimen Transfer Form must be completed and faxed to GSK Biologicals to the number provided below. A copy of the Specimen Transfer Form must be in the parcel.[1]

GLAXOSMITHKLINE BIOLOGICALS
Attention: Biospecimen Reception
R & D Department/Building 44
Rue de l'Institut, 89
B-1330 Rixensart – Belgium
Telephone: +32-2-656 8949 or +32-2-656 6130
or +32-2-656 8549 or +32-2-656 6108
Fax: +32-2-656 6052
E-mail: rix.ugbiospecimen-reception@gskbio.com

Note: Handling of throat swabs are detailed in Appendix H.

[1] The Serum Listing Form and the Specimen Transfer Form are standard documents used in GSK Biologicals' clinical trials. These documents are provided by GSK Biologicals' Clinical Trials' monitor at study initiation.

Appendix E: Shipment of Biological Samples

Instructions for Shipment of Serum Samples

Serum samples should be sent to GSK Biologicals at regular intervals. The frequency of shipment of samples should be decided upon by the Site Monitor, Central Study Coordinator and the investigator prior to the study start.

Serum samples should always be sent by air, preferably on a Monday, Tuesday or Wednesday, unless otherwise requested by the sponsor.

Serum samples must be placed with dry ice (-20°C) in a container complying with International Air Transport Association (IATA) requirements. The completed standard serum listing form should always accompany the shipment.

The container must be clearly identified with the labels provided by GSK Biologicals specifying the shipment address and the storage temperature (-20°C).

The airway bill should contain the instruction for storage of samples at -20°C.

A "proforma" invoice, stating a value for customs purposes only, should be prepared and attached to the container. This document should contain the instruction for storage of samples at -20°C.

Details of the shipment, including:

* number of samples
* airway bill
* flight number
* flight departure and arrival times

should be sent by fax, two days before shipment, to:

GLAXOSMITHKLINE BIOLOGICALS
Attention: Biospecimen Reception
R & D Department/Building 44
Rue de l'Institut, 89
B-1330 Rixensart – Belgium
Telephone: +32-2-656 8949 or +32-2-656 6130
or +32-2-656 8549 or +32-2-656 6108
Fax: +32-2-656 6052
E-mail: rix.ugbiospecimen-reception@gskbio.com

Appendix F: Laboratory Assays

Section 5.5.2 Laboratory assays.

Appendix G: Vaccine supplies, packaging and accountability

It is NOT permitted to use any of the supplies provided by GSK Biologicals for purposes other than those specified in the protocol. Unused supplies will be collected by GSK Biologicals on completion of the study. Used vaccine vials can be disposed on site according to local biosafety standard for disposal of biological waste material.

1. Vaccine supplies

GSK Biologicals will supply the following amounts of numbered doses of study vaccines, sufficient to administer 3 doses to all subjects as described in the present protocol.

- 420 doses of Hib-MenAC vaccine in monodose vials
- 420 doses of Hiberix™ vaccine in monodose vials
- 1260 doses of Tritanrix™-HepB vaccine in monodose vials
- 280 doses of Mencevax™ AC
- 560 doses of 0.5 ml diluent for the Mencevax™ AC vaccine

An additional quantity of at least 5% of Tritanrix™-HepB and Hiberix™ will be supplied for replacement in case of breakage, bad storage conditions or any other reason that would make the vaccine unusable (i.e., given by mistake to another subject).

All monodose vials must be accounted for on the form provided.

In case of suboptimal response to an antigen (refer to Section 5.5.4) GSK Biologicals will also supply doses of Mencevax™ ACWY or Tritanrix™-HepB/Hiberix™ vaccines on a case by case basis.

2. Vaccine packaging

The vaccines will be packed in labelled boxes. In order to ensure the double blind of the study, all vaccine boxes will be identical (size and appearance), despite the different number of vials needed for reconstitution of the vaccines. Only the vaccination team will open the boxes for reconstitution and administration of the vaccine so only the vaccination team will know which vaccine is contained in the individual boxes (see section 6.4). The box label will contain, as a minimum, the following information: study number, subject number, lot number (or numbers, when double-blind), instructions for vaccine administration.

3. Vaccine accountability

The investigator or pharmacist must sign a statement that he/she has received the clinical supplies for the study. At all times the figures on supplied, used and remaining vaccine

doses should match. All used vials should be placed in sealed envelopes by the vaccination team in order to maintain the blinding of the study. After the end of the study and unblinding, it must be possible to reconcile delivery records with those of used and unused stocks. An explanation must be given of any discrepancies.

After approval from GSK Biologicals, used vaccine vials should be destroyed at the study site using locally approved biosafety procedures and documentation unless otherwise described in the protocol. If no adequate biosafety procedures are available at the study site, the used vaccine vials are to be returned to an appropriate GSK Biologicals site for destruction in accordance with current GSK SOP NPD-112. Unused vaccine vials will be disposed at the local GSK Biologicals site in accordance with GSK SOP NPD-112. If no processes for destruction of unused vaccines are in place in the local GSK Biologicals site; the unused vials must be returned to GSK Biologicals in Rixensart, Belgium.

4. Transfers of clinical vaccines or products from country medical department or dispatch center to study sites or between sites
Storage temperatures must be maintained during transport and deviations must be reported to Logistics and Packaging for guidance. All transfers of clinical vaccines or products must be documented using the Clinical Supply Transfer Form. If the duration of the transfer is less than four hours, a transportable fridge or any suitable container (e.g. styrofoam container) with a maximum of eight refrigerated cold packs (cooling elements) must be used in order to maintain the vaccines at 2°-8°C during transport. If the duration is more than four hours, a transportable fridge or any suitable container (e.g. styrofoam container) with a minimum of eight cold packs (cooling elements) must be used as well as a temperature monitoring system that must be placed as close as possible to the doses and checked upon reception at the final destination. Never place frozen cold packs or dry ice inside vaccine/product boxes for vaccine that must be kept at +4°C in order to avoid cold-chain deviation (e.g. frozen vaccines). Exceptions to these instructions are detailed in product-specific transport guidelines.

5. Labels for sample identification
The investigator will receive labels from GSK Biologicals to identify samples taken from each subject at each timepoint. Each label will contain the following information: study number, subject number, sampling timepoint (e.g., post vacc 3), timing (e.g., study Month 3).

6. Other supplies provided by GSK Biologicals
In addition to the vaccines, the study documentation and the sample labels, the investigator will receive the following supplies:

- tubes for blood sampling,
- tubes with screw caps for serum samples,

- racks for the tubes of serum
- butterflies, needles for reconstitution and injection, thermometers, waste disposals for used materials

Appendix H: Meningococcal carriage in the African meningitis belt

Meningococcal carriage in the African meningitis belt

**Date of Approval**	First Version: 14 January 2003<br>Revised Version: **Amended 27 January 2004**
**Title**	**Meningococcal carriage in the African meningitis belt**,<br><br>an ancillary study to<br><br>A phase II, double blind, randomized, controlled study to evaluate the immunogenicity, reactogenicity and safety of GlaxoSmithKline (GSK) Biologicals' Hib-MenAC vaccine, extemporaneously mixed with GSK Biologicals' Tritanrix™-HepB, as compared to GSK Biologicals' Hiberix™ vaccine, extemporaneously mixed with GSK Biologicals' Tritanrix™-HepB, when administered intramuscularly in infants at 6, 10 and 14 weeks of age.

Co-Sponsor, Swiss Tropical Institute (STI), Basel, Switzerland

Co-investigator: Dr. Gerd Pluschke, Molecular Immunology

Navrongo Health Research Center (NHRC), Navrongo, Ghana

Principal Investigator: Dr. Abraham Victor Obeng Hodgson
Co-investigator: Doctor Abudulai Adams Forgor

World Health Organization (WHO), Geneva, Switzerland

Collaborating Person: Dr. Marie-Paule Kieny

London School of Hygiene and Tropical Medicine

Collaborating Person: Professor Brian Greenwood

Collaborating Person: Dr. Daniel Chandramohan

GlaxoSmithKline Biologicals

Collaborating Person: Dominique Boutriau

Medical Monitor

GSK Biologicals:
Dominique Boutriau,
Director, Clinical Research &
Development, Meningitis vaccines
GlaxoSmithKline Biologicals,
89, Rue de l'Institut, 1330 Rixensart,
Belgium
Tel: + 32 2 656.91.20
Fax: + 32 2 656. ***8044***
e-mail: dominique.boutriau@gskbio.com

(Amendment 7: 12 April 2006)

Study Monitors

Cornelia Bevilacqua,
Central Study Coordinator,
'La Fédarié Haute'
81440 Vénès
France
Tel: + 33 563 75 92 66
Fax: + 33 563 75 92 66
Mobile phone: + 33 685 25 24 69
e-mail: corneliabevilacqua@usa.net

Valérie Marichal,
Central Study Coordinator,
GlaxoSmithKline Biologicals,
89, Rue de l'Institut, 1330 Rixensart, Belgium
Tel: + 32 2 656.9812
Fax: + 32 2 656.8133
e-mail: valerie.marichal@gskbio.com

Local Safety Monitor

Dr. John Williams
Navrongo Health Research Center,
Ministry of Health, PO Box 114, Navrongo, Ghana
Tel: ++ 233 742 22310/ 22380
Fax: ++ 233 742 22320
e-mail: jwilliams@navrongo.mimcom.net

Local Safety Monitor Back-up

Dr. Frank Baiden
Navrongo Health Research Center,
Ministry of Health, PO Box 114, Navrongo, Ghana
Tel: ++ 233 742 22310/ 22380
Fax: ++ 233 742 22320
e-mail: fbaiden@navrongo.mimcom.net

WHO Monitor

Dr.Lawrence Kweku Yamuah
Post-Doctoral Researcher / Data Manager
Armauer Hansen Research Institute (AHRI)
P.O. Box 1005
Addis Ababa
Ethiopia
Tel: + 251 1 71 02 88 (work)
Tel: + 251 1 71 17 04 (home)
Fax: + 251 1 71 13 90
yamuahlawrence_kweku@hotmail.com

WHO Study Contact for Reporting SAEs

Dr. Zarifah Reed
WHO/**VAB**/IVR/POP
20 Avenue Appia
CH-1211 GENEVE 27
Switzerland
Tel. +41 22 791 4760
Fax. +41 22 791 4860
Email: reedz@who.int

I PURPOSE OF THE ANCILLARY STUDY AND BACKGROUND

1. Purpose of the ancillary study

Aim of the ancillary colonization study is to investigate how frequently and for how long infants <1 year are colonized by *N. meningitidis* and *N. lactamica* in the study area. This will provide important baseline data allowing sample size calculations in later studies accompanying the introduction of a meningococcal conjugate vaccine and addressing the issue of potential serogroup replacement by mass vaccination. The proposed ancillary study will also address the question, whether colonization by A meningococci is reduced following meningococcal conjugate vaccination.

2. Background

African countries, and more specifically the countries of the meningitis belt, face regular epidemics of meningococcal disease, including meningitis with an incidence as high as 1000/100,000. Except for an outbreak of serogroup W-135 that spread in Burkina Faso in 2002, serogroup A and to a lesser extent serogroup C are largely responsible for *N. meningitidis* epidemics in African countries (1). Children aged 5-14 years are the most

affected. The existing meningococcal plain polysaccharide vaccines are less immunogenic in small children; also the duration of protection induced by these vaccines is short-lived, as polysaccharides vaccine do not induce immune memory. Therefore, there is a need to develop new generation meningococcal vaccines that protect against meningococcal disease in early childhood and induce immune memory (2, 3). Recently, meningococcal serogroup C conjugate vaccines have been developed and have proven effective in small children and infants (4).

In order to protect children from early childhood against *N. meningitidis* of the two most prevalent serogroups in Africa, and to facilitate the incorporation of such a vaccine in the immunization calendar, GSK Biologicals is developing a combined Hib-MenAC conjugate vaccine to be extemporaneously mixed with the Tritanrix™-HepB (DTPw-HBV) vaccine for administration as a 3-dose primary vaccination course in infants aged 6 weeks and over. The new Tritanrix™-HepB/Hib-MenAC vaccine will first be evaluated in a feasibility trial outside Africa. Once feasibility of the new vaccine is shown, the obove mentioned core study plans to evaluate the use of GSK Biologicals' Tritanrix™-HepB/Hib-MenAC vaccine in a high endemicity region in Africa.

In 1998 an ongoing longitudinal study of the dynamics of carriage of *Neisseria meningitidis* and *N. lactamica* was started by STI and NHRC in the Kassena-Nankana District of Northern Ghana (5-9). In 6-monthly field surveys (once in the dry season and once in the wet season of each year) 37 compounds randomly selected from a complete listing of the district population (Navrongo Demographic Surveillance System) are visited and *Neisseria* carriage of the total population of these compounds is monitored. The studies have provided information on longitudinal patterns of colonisation with multiple types of *N. meningitidis and N. lactamica*, and spatio-temporal patterns of colonisation and disease during and before a potential new epidemic. The data indicate that in contrast to Europe and the USA, carriage of non-encapsulated meningococci and other non-pathogenic meningococci is infrequent in Africa. It is thought that carriage of non-serogroupable meningococci protects against meningococcal disease by eliciting cross-reactive immunity against pathogenic strains. Therefore, in Africa, low levels of carriage may result in inadequate levels of naturally acquired immunity and thus an increased susceptibility of the population to meningococcal epidemics.

After a *N. meningitidis* serogroup A epidemic in the Kassena-Nankana district in 1997 serogroup A carriage decreased and for a period of about two years serogroup A meningococci were neither isolated from cases nor from carriers. However, after a period of only four years, a new serogroup A outbreak is now being observed, indicating that the decline in herd immunity may not be the crucial factor determining time intervals between epidemics. Molecular typing revealed that the current outbreak is caused by a different serogroup A clone (sequence type 7, a clone which was for the first time observed in 1995 in Africa) than the previous epidemic (sequence type 5, a clone which

caused epidemics first in Mecca (1987) and subsequently in several nations of the meningitis belt). Between the two outbreaks, a carriage epidemic of serogroup X meningococci was observed, coinciding with a number of cases caused by serogroup X meningococci. Currently it is not clear whether X meningococci have an epidemic potential (like W-135 meningococci, which are the cause of a recent epidemic in Burkina Faso associated with an unusually high mortality). The recent increase in outbreaks caused by non-A non-C meningococci may reflect serogroup replacements caused by mass vaccination with serogroup A/C carbohydrate vaccine. Similarly, shifts in the serogroups of *N. meningitidis* causing disease could potentially arise as a result of immune selection resulting from mass-vaccination with conjugate vaccines. In view of recent outbreaks caused by W-135- and X meningococci this possibility is a real concern. Therefore, the database on longitudinal patterns of colonisation with multiple types of *N. meningitidis*, and the spatio-temporal patterns of colonisation and disease provides a unique baseline from which to carry out trials on the effects of vaccination on acquisition of meningococcal infection and on serogroup replacement.

REFERENCES

1. Greenwood B. Manson Lecture. Meningococcal meningitis in Africa. Trans R Soc Trop Med Hyg 1999;93:341-353.
2. Jodar L, Feavers IM, Salisbury D, Granoff DM. Development of vaccines against meningococcal disease. Lancet 2002;359:1499-1508.
3. MacLennan J, Obaro S, Deeks J, Lake D, Elie C, Carlone G, Moxon ER, Greenwood B. Immunologic memory 5 years after meningococcal A/C conjugate vaccination in infancy. J Infect Dis 2001;183:97-104.
4. Maiden MC, Stuart JM. Carriage of serogroup C meningococci 1 year after meningococcal C conjugate polysaccharide vaccination. Lancet 2002;359:1829-1831.
5. Gagneux, S., Hodgson, A., Ehrhard, I., Morelli, G., Genton, B., Smith, T., Tanner, M., Binka, F., Achtman, M., and Pluschke, G. Microheterogeneity of serogroup A subgroup III Neisseria meningitidis during an outbreak in Northern Ghana. Trop. Med & Int. Health, 2000; 5, 280
6. Hodgson, A., Smith, T., Gagneux, S., Adjuik, M., Pluschke, G., Mensah, N. K., Binka, F. and Genton, B.,Risk factors for meningococcal meningitis in northern Ghana. Trans. Royal Soc. Med. & Hygiene, 2001; 95, 477 - 480
7. Hodgson A, Smith T, Gagneux S, Akumah I, Adjuik M, Pluschke G, Binka F, and Genton B. Survival and sequelae of meningococcal meningitis in Ghana. Int J Epidemiol., 2001; 30, 1440 - 1446
8. Gagneux, S., Hodgson, A., Smith, T., Wirth, T., Ehrhard, I., Morelli, G., Genton, B., Binka, F., Achtman, M. and Pluschke, G., Prospective study of a serogroup X *Neisseria meningitidis* outbreak in Northern Ghana. J. Infect. Diseases, 2002; 185, 618-626

9. Gagneux, S., Hodgson, A., Wirth, T., Ehrhard, I., Morelli, G., Kriz, P., Genton, B., Smith, T., Binka, F., Pluschke, G. and Achtman, M., Clonal Groupings in serogroup X *Neisseria meningitidis*. Emerging Infectious Diseases, 2002; 8, 462 - 466

II CHARACTERISTICS OF THE RESEARCH POPULATION

1. Number, gender, age, and race of subjects

Target enrolment of the core vaccine study will be 280 healthy male and female infants (140 subjects per group) to provide 220 evaluable subjects (110 per group) for the core immunogenicity analysis.

For the ancillary colonization study, throat swabbing of all infants and their consenting parents/guardians will be done. Samples will be taken from all children enrolled in the core study.

The study will be conducted by the Navrongo Health Research Center in Ghana at the War Memorial Hospital (serving Navrongo central) and at other health centres in the Kassena-Nankana health district.

Enrolment will be terminated when 280 infants have been enrolled for the core vaccine study.

Currently there is hardly any information available on the frequency and duration of meningococcal colonization of infants <1 year in the study area. Since there is concern that mass vaccination with a meningococcal conjugate vaccine might result in serogroup replacement it is important to investigate this aspect in the target group of an EPI-associated meningococcal conjugate vaccine. The ancillary study is expected to give first insight, thus providing important baseline data allowing sample size calculations in later studies accompanying the introduction of a meningococcal conjugate vaccine. Our long term colonization studies indicate that meningococci spread very fast within individual compounds. A colonization analysis of parents or guardians will provide insight into the frequency of this spread to infants.

The intended racial and ethnic distribution is determined by the fact that the study should be performed in a population at high risk for epidemic meningococcal meningitis; i.e inhabitants of the African meningitis belt.

2. Inclusion criteria

All subjects must satisfy the following criteria at study entry:

- Subjects for whom the investigator believes that their parents/guardians can and will comply with the requirements of the protocol (e.g., completion of the diary cards, return for follow-up visits) should be enrolled in the study.
- A male or female between, and including, 6 and 8 weeks of age at the time of the first vaccination.
- Written informed consent obtained from the parent or guardian of the subject for the participation of the subject and of the parent/guardian.
- Free of obvious health problems as established by medical history and clinical examination before entering into the study.

3. Exclusion criteria for enrolment

The following criteria should be checked at the time of study entry. If any apply, the subject must not be included in the study:

- Use of any investigational or non-registered drug or vaccine other than the study vaccine(s) within 30 days preceding the first dose of study vaccine, or planned use during the study period.
- Chronic administration (defined as more than 14 days) of immunosuppressants or other immune-modifying drugs since birth. (For corticosteroids, this will mean prednisone, or equivalent, ≥ 0.5 mg/kg/day. Inhaled and topical steroids are allowed.)
- Planned administration/ administration of a vaccine not foreseen by the study protocol within 30 days before each dose of vaccine, with the exception of OPV.
- Hepatitis B and BCG vaccine given within two weeks prior to vaccination.
- Previous vaccination against diphtheria, tetanus, pertussis, *Haemophilus influenzae* type b or meningococcal serogroup A or C diseases.
- History of diphtheria, tetanus, pertussis, hepatitis B, *Haemophilus influenzae* type b or meningococcal serogroup A or C diseases.
- Known exposure to (direct contact) diphtheria, tetanus, pertussis, hepatitis B, *Haemophilus influenzae* type b and/or meningococcal disease since birth.
- A diagnosis or clinical suspicion of an immune suppressive or immunodeficient condition of any cause based on full clinical history and medical examination.
- A family history of congenital or hereditary immunodeficiency.
- History of allergic disease or reactions likely to be exacerbated by any component of the vaccine.
- Major congenital defects or serious chronic illness.
- Babies for which birth weight is <2 kg (if known), and/or malnutrition at visit 1.
- History of any neurologic disorders or seizures.

- Acute disease at the time of enrolment. (Acute disease is defined as the presence of a moderate or severe illness with or without fever. All vaccines can be administered to persons with a minor illness such as diarrhea, mild upper respiratory infection with or without low-grade febrile illness, i.e., axillary temperature <37.5°C or rectal temperature <38.0°C).
  Note: Subjects with fever at the time of first vaccination (i.e., axillary temperature ≥ 37.5°C or rectal temperature ≥ 38.0°C) need not necessarily be excluded but can be vaccinated at a later date after the subject recovers and if the subject still meets all eligibility criteria.
- Administration of immunoglobulins and/or any blood products since birth or planned administration during the study period.
- Other conditions which in the opinion of the investigator may potentially interfere with interpretation of study outcomes.

4. Elimination criteria during the study

The following criteria should be checked at each visit subsequent to the first visit. If any become applicable during the study, it will not require withdrawal of the subject from the study but does determine a subject's evaluability in the according-to-protocol (ATP) analysis.

- Use of any investigational or non-registered drug or vaccine other than the study vaccine(s) during the study period.
- Chronic administration (defined as more than 14 days) of immunosuppressants or other immune-modifying drugs during the study period. (For corticosteroids, this will mean prednisone, or equivalent, ≥ 0.5 mg/kg/day. Inhaled and topical steroids are allowed).
- Administration of a vaccine not foreseen by the study protocol during the period starting from 30 days before the first dose of vaccine(s) and ending 30 days after the last dose of vaccine(s) with the exception of OPV.
- Administration of a meningococcal vaccine not foreseen by the study protocol during the entire study period.
- Administration of immunoglobulins and/or any blood products during the study period.

5. Withdrawal of subject from core study

If a study subject is withdrawn from the core study, that subject will also be withdrawn from the ancillary study.

III METHODS AND PROCEDURES

1. List of study procedures (core study)

Age	±6 w	±10 w	±14 w	±18 w	± 9 m	±12 m	±13 m
Visit	**VISIT 1**	**VISIT 2**	**VISIT 3**	**VISIT 4**	**VISIT 5**	**VISIT 6**	**VISIT 7**
Timing	Month 0	Month 1	Month 2	Month 3	Month 7.5	Month 10.5	Month 11.5
Sampling time point	Pre vacc			Post vacc III		Pre booster	Post booster
Informed consent#	•						
Check inclusion criteria	•						
Check exclusion criteria	•						
Check elimination criteria		•	•	•	•	•	•
Check contraindications		•	•		○##	•	
Medical history	•						
Physical examination	•	•	•	•	•	•	•
Pre-vaccination body temperature	•	•	•		•	•	
Measure/record height and weight	•						
Randomization	•						
Blood sampling for antibody determination (3.5 ml)	•			•		•	•
Throat swab	•**	•**		•**	•**	•**	
Vaccination	•	•	•		•***	•****	
Daily post-vaccination recording of solicited symptoms (days 0-3) by field workers	•	•	•			•	
Recording of non serious adverse events occurring one month (minimum 30 days) post-vaccination, by investigator	•	•	•	•		•	•
Return of diary cards		•	•	•			•
Diary card transcription		•	•	•			•
Record any concomitant medication	•	•	•	•	•	•	•
Reporting of Serious Adverse Events	•	•	•	•	•	•	•
Study Conclusion							•

• denotes a procedure which must be recorded in the individual CRF.
° denotes a procedure which need not be recorded in the individual CRF.
any parent/guardian accompanying the child at visits subsequent to the first visit who has not already signed an Informed Consent for this study will have to sign an Informed Consent prior to providing any throat swab
.## Contraindications for measles and yellow fever vaccines.
** from the subject and subject's parents/guardians; ***Measles, yellow fever vaccine
****1/5th of dose of Mencevax™ AC
Note: BCG and OPV vaccines are given at birth (or at least 2 weeks before the subject's first visit) according to the nationally recommended immunization schedule.
OPV vaccine can be administered to the subjects at 6, 10 and 14 weeks of age concomitantly with the study vaccines or at any time during the study, whichever is more appropriate.

2. Methods and procedures specific for the ancillary study

Throat swabbing of consenting parents/guardians and infant subjects will also be done at age 6w, 10w, 18w, 9m and 12m.

This non-invasive procedure is done using a sterile swab on stick. After consent is obtained from the subject (or his/her parent/guardian), the subject is asked to open the mouth as wide as possible and bend the head slightly backward. Children are assisted to bend their heads by an assistant. A trained investigator wearing disposable gloves depresses the tongue with a spatula in one hand. The subject is asked to say ah-ah (if an adult). The investigator picks the sterile swab with the other hand and passes it through the mouth gently rubs it against the posterior wall of the pharynx or behind the tonsils without touching the uvula and tongue. The swab stick is removed from the mouth and immediately inoculated directly on Thayer-Martin agar plates (Difco), which is labeled, and transported to the hospital laboratory within 2 hours for incubation in a candle jar at 37ºC for 24 hours.

THROAT SWAB BEING TAKEN FROM A SUBJECT

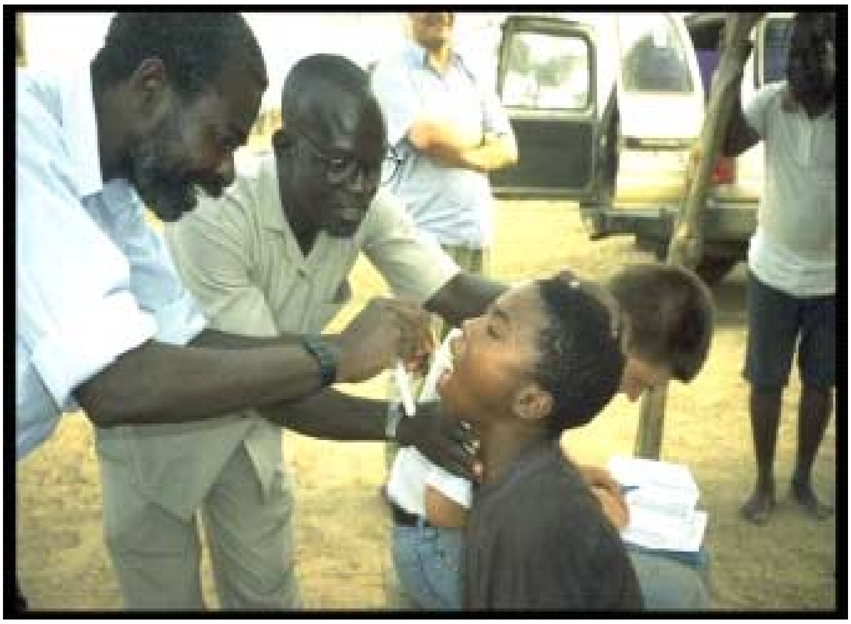

Neisseria-like colonies will be picked and characterized by standard microbiological procedures in Navrongo. Isolates are then frozen and transported to Switzerland for detailed molecular characterization. N. meningitidis or N. lactamica are identified by Gram’s stain morphology, cytochrome oxidase test using N,N,N’,N’-tetramethyl-1,4-phenylene-diammoniumdichloride as substrate, glucose, maltose and sucrose utilization

in cystine-trypticase agar, ONPG discs and gamma-glutamyltransferase activity. All isolated meningococci will be serogrouped by slide agglutination with serogroup specific antisera and serotyped/subtyped with monoclonal antibodies by whole cell ELISA. Further analysis is carried out using pulsed-field gel electrophoresis and for a sub-set of isolates representing the different PFGE variants multi locus sequence typing. N. meningitidis isolates are tested for sensitivity to penicillin G, cefotaxime, ciprofloxacin, rifampicin, chloramphenicol, tetracycline and sulfadiazine by the E-test method (AB Biodiscs, Solna, Sweden) according to the manufacturer's instructions.

3. Data analysis and data monitoring

3.1 Safety monitoring

An independent committee consisting of up to five experts in meningococcal diseases, paediatrics, vaccines, statistics and other appropriate disciplines will be appointed to oversee ethical and safety aspects of the study conduct.

Terms of reference (operating procedures) of the IDMC will be written after the first meeting of the IDMC members, prior to study start.

3.2 Role of the IDMC

The role of the IDMC includes the review of the implementation and progress of the study. It provides initial, regular, and closing advice on safety-related issues to GSK Biologicals. Its advice is based on the interpretation of study data with reference to the study protocol.

The IDMC will confer before the initiation of the study (pre-initiation review), during the study proper and at the close out of the study to review any relevant safety data and to review and approve the Report and Analysis Plan (RAP), where applicable. Other unscheduled meetings may be required. Meetings must be documented and minutes made available for the study files on site and to the sponsors. The IDMC may, if deemed necessary, convene a meeting with or request further information from the Principal Investigators, the Local Safety Monitor, GSK Biologicals and designated project representatives at any stage of the study.

The IDMC must be informed by the investigator of:

- all subsequent protocol amendments, informed consent changes or revisions of other documents originally submitted for review
- systemic grade 3 adverse events (AEs) related to vaccination**,** serious adverse events and adverse events suggesting a lack of efficacy of the vaccine occurring during the study (transmitted through GSK Biologicals for SAEs and through the Local safety Monitor for the other adverse events)

- all subsequent protocol modifications (for information)
- new information that may affect adversely the safety of the subjects or the conduct of the study.

The **IDMC** will be empowered to put the study on hold pending review of potential safety issues. All SAEs, including death, will be reported by the Principal Investigator to the local safety monitor, to the Manager of Clinical Safety Vaccines at GSK Biologicals, to WHO and to the WHO safety monitor. GSK Biologicals will be responsible to provide all information related to the SAEs to the IDMC according to the IDMC Charter.

3.3 Reporting to IDMC **– Role of Local Safety Monitor**

A Local Safety Monitor (LSM) will act independently from the investigational team.
The overall role of the Local Safety Monitor (LSM), who will be an experienced clinician based in-country, will be to support the clinical investigators and to act as a link between the investigators and the IDMC. All systemic grade 3 AEs probably or suspected to be related to vaccination, all SAEs and all AEs suggesting a lack of efficacy of the vaccine will be reported to him/her. His/her involvement will be particularly important when decisions have to be made quickly. In exceptional circumstances, for example a death possibly related to vaccination, he/she will have the authority to suspend the whole or any specific aspect of the trial pending discussion with the IDMC.
The LSM’s role will include:

- acting as the study volunteer's advocate
- provide the IDMC and the medical monitor of GSK Biologicals with a blinded listing of non-serious systemic grade 3 symptoms (solicited and unsolicited) that are related with vaccination, and of non-serious adverse events suggesting a lack of efficacy of the vaccine, this according to the IDMC Charter (note: GSK Biologicals will send all information related to serious adverse events to the IDMC according to the IDMC Charter).
- reviewing blinded reactogenicity data according to IDMC charter after each dose and discussing with IDMC if necessary
- promptly communicating relevant safety information to the IDMC
- providing advice to the investigators on whether a set of clinical circumstances in a study warrants formal notification to the IDMC
- providing clinical advice on any illness in study subjects especially in circumstances in which treatment might influence the course of the trial
- Unblinding a subject if deemed necessary, upon request of the investigator/physician in charge of the subject, to allow for adequate treatment.

The LSM will liaise closely with the chair of the IDMC throughout the course of the trial.

3.4 **Data analysis for ancillary study**

The ancillary study represents a pilot exploratory analyses that will provide important baseline data for later more extended studies. Only rough estimates can therefore be made at this stage about outcomes and types of statistical data analyses required.

It is expected that the proposed sampling of 280 infants at age 6w, 10w, 18w, 9m and 12m will provide much more detailed insight in the relative level of colonization and the duration of colonization in this age group than previous research. Results gained in this pilot study would therefore strongly facilitate the design of a more extended carriage study accompanying the large scale introduction of a conjugate vaccine. Furthermore, the sample size of 130 in each group will already give >80% power to detect a conjugate vaccine-associated 90% reduction in the number of children carrying A meningococci, assuming that in the control group approximately 5% of children carry these bacteria in one or more of the sequential samplings.

3.5 Data storage and confidentiality

Samples will be coded with running numbers. Records that correlate these numbers with the name of the patient will remain with the investigators of the core study and will not be provided to the researchers responsible for the ancillary study.

Electronic data will be stored in password-protected computers, and data in hardcopy form will be kept in a locked cabinet, accessible only to authorized study personnel.

Data from the ancillary study will be provided to the trial monitors and sponsors of the core study, upon request.

Publications of research results will not include any names or any personal information that could reasonably be associated with any research participant.

IV RISK/BENEFIT ASSESSMENT

1. Risk category

Risk associated with the throat swabbing procedure for the ancillary studiy is minimal.

2. Potential risk and protection against risk

Throat swabbing is considered a low risk procedure and is not known to produce substantial stress or discomfort.

The risk of stress or discomfort from the procedures will be further reduced, through the training of the investigators performing these procedures, which have been clearly described in writing (see herein).

In addition, there will be close monitoring for adverse events, largely because of potential

risks posed to participants by virtue of their participation in the vaccination clinical trial:

Field workers will be trained on the definition of adverse events, including serious adverse events, under the supervision of the Principal Investigator. On the day of vaccination of any study vaccine and on each of the three subsequent days or up to resolution of symptoms, whichever is longer, field workers will visit each research participant at his/her home to record any adverse events. Field workers will also visit each research participants one month after the vaccination of any study vaccine and will observe the research participant for any adverse events.

The Principal Investigator will report each serious adverse event within twenty-four (24) hours of becoming aware of such event. The Principal Investigator will report such event to the Local Safety Monitor, the WHO monitor and GSK Biologicals safety department. GSK Biologicals safety department will transmit the information to the IDMC according to the operating procedures of the IDMC. In addition, the investigator will also provide a listing of all systemic grade 3 symptoms suspected to be related to vaccination and any case of diphtheria, tetanus, pertussis, hepatitis B, invasive diseases due to *Haemophilus influenzae* type b, measles, yellow fever or *Neisseria meningitidis* of serogroup A and C to the LSM. The Local Safety Monitor will send the listing and information related to the non-serious grade 3 systemic symptoms related with vaccination and non-serious adverse events suggesting a lack of efficacy of the vaccine to the IDMC and the GSK medical monitor (the GSK medical monitor will forward the information to the other members of the Steering Committee) **according to the Charter for the IDMC**.

The Principal Investigator will proactively follow each subject (a) with a serious adverse event or who has withdrawn from the study as a result of an adverse event, until the event has resolved, subsided, stabilized, disappeared, the event is otherwise explained, or the subject is lost to follow-up; (b) with a non-serious adverse event who has not withdrawn from the study, until the subject completes the study or is lost to follow-up; or (c) with clinically significant laboratory abnormalities, until the subject’s conditions return to normal or a satisfactory explanation has been provided.

Updated information, including completed verbal autopsy questionnaire in case of death, will be recorded on each serious adverse event Report Form, and the Form will be signed and dated by the Principal Investigator. Updated information will be reported in the same manner as the original serious adverse event information.

After the study end, the Principal Investigator is not obliged to actively seek for adverse events or serious adverse events in former research participants. However, if the Principal Investigator learns of any serious adverse event, including a death, at any time after a research participant has been discharged from the study, and he considers the event

reasonably related to the study, the Principal Investigator will promptly notify the study contact for reporting serious adverse events.

3. Potential benefits

Individuals participating in the ancillary study will have no direct benefit. The data from the ancillary studies will, however, enable more precise sample size calculation in later studies which will follow the introduction of a meningococcal conjugate vaccine, addressing the potential for serogroup replacement. This is of considerable potential importance and benefit for the population of the African meningitis belt.

4. Alternatives to participation

After one parent/guardian has consented to participate in the ancillary study and core study, any other parent/guardian who accompanies the infant to a clinical trial site visit will have the option not to participate in the ancillary study. Subjects can withdraw from the core study and ancillary study at any time.

V SUBJECT IDENTIFICATION, RECRUITMENT AND CONSENT/ASSENT

1. Subject identification and recruitment

1.1. General enrollment information

The study will be conducted in the Kassena-Nankana district by the Navrongo Health Research Center in Ghana.

- All subjects should be enrolled within a period of 5 months.
- Anticipated recruitment rate is 56 subjects per month. Subjects will be recruited when they present themselves at the immunization clinics.
- Enrolment will be terminated when 280 subjects are enrolled.
- The recruitment will be monitored by weekly update through regular contacts with study monitors.

1.2 A community information campaign will be conducted as detailed below:

Prior to the start of the study, the Regional Director of Health Services will be briefed about the study and the start dates. The District Director of Health Services will be reminded of the start of the study and the use of the health facilities at the three sub districts during that period. The Medical Superintendent of the district hospital, War Memorial Hospital, will also be informed.

Meetings will be arranged with the District Chief Executive as well as paramount chiefs, sub chiefs and elders of the three sub districts separately at their convenience to explain the study to them and seek their consent.

At Navrongo central, the Navropio, his sub chiefs and elders will be met at the Navropio's palace. The Kologo chief, his sub chiefs and elders will be met at Kologo whilst the Bui chief and his elders will be met at Bui.

In the East sub district, separate meetings will be held with the chiefs and elders of Sirigu, Yua, Kandiga, Nabogo, Mirigu, Natugnia and Amontanga as in the table below.

At these meetings, the study will be explained to them, questions related to the study answered and their consent solicited to start the study.

1.3 Recruitment through the Navrongo War Memorial Hospital:

Mothers delivering at the Navrongo War Memorial Hospital will be informed about the study and permission solicited to contact them in future about their potential participation in the trial.

1.4 Recruitment through the NDSS

Potential study participants will also be identified from the demographic surveillance system of the Kassena-Nankana health district, the Navrongo Demographic Surveillance System (NDSS). The NDSS was established by the Ghana Ministry of Health for the purpose of enhancing research in the Kassena-Nankana district. The NDSS serves as the platform from which studies draw potential research participants. At the start of the study, a request is made to the head of the NHRC for access to the NDSS data set; once he approves, the requested data can be accessed. The approval for this study is attached.

1.5 Recruitment by field workers

Routine childhood immunizations are provided on fixed days at the Navrongo War Memorial Hospital and health clinics. One day before first immunization, the field workers will visit the potential study participants to invite them to attend the hospital/health centre the next day.

2. Process and documentation of consent

On the day of immunization, prior to any study procedure, the Parent Information Sheet and Informed Consent forms will be read and explained to the subject’s parents/guardians in the local languages (Kassem and Nankam) and according to the local custom and questions will be answered. Literate parents/guardians will be given the Parent Information sheet and Informed Consent form to read.

When agreement is given by the parent/guardian, the IC is signed or thumbprinted (illiterates) by the parents/guardians of the subject, witnessed by an independent person.

Consent documentation and records that correlate study numbers with the name of the patient will remain with the Principal Investigator of the core study and will not be provided to the researchers responsible for the ancillary study. Hardcopy documents will be kept in a locked cabinet, accessible only to authorized study personnel.

3. Costs/inconvenience of participation

In terms of the inconvenience the study poses to the participants, the maximum distance between a subject's compound and the nearest health facility will be ten kilometers. Each visit to the clinic for vaccination and throat swabbing will last approximately four hours, though the throat swabbing procedure will last approximately one minute.

4. Payment for participation

Subjects will not receive reimbursements or payments for their participation.

In case of reply the number and date of this letter should be quoted.

My Ref.:
Your Ref. No.

GHANA HEALTH SERVICE
Your Health-Our Concern

Navrongo Health Research Centre
Ghana Health Service
P. O. Box 114
Navrongo

Tel: +233-742 22380
Fax: + 233-742 22320

Email:
ahodgson@navrongo.mimcom.ne[illegible]

April 15, 2003

ACCESS TO NDSS DATASET

This is to inform you that the investigators of the Phase II, double blind, randomized, controlled trial to evaluate the immunogenicity, reactogenicity and safety of Glaxosmithkline (GSK) Biologicals' Hib-Men AC vaccine will have access to the Navrongo Demographic Surveillance System (NDSS) dataset for the purposes of the study.

Yours sincerely,

[signature]

Dr Abraham Hodgson
Director, NHRC

Appendix I Amendments and Modifications to the Protocol

GlaxoSmithKline Biologicals
Rue de l'Institut 89
1330 Rixensart, Belgium

Protocol Amendment Approval

Study Drug

Hib-MenAC

Protocol Number

759346/009 (***DTPwHB/HIBMenACTT-009***) **(Amended 05 June 2003)**

Protocol Title: A phase II, double blind, randomized, controlled study to evaluate the immunogenicity, reactogenicity and safety of GlaxoSmithKline (GSK) Biologicals' Hib-MenAC vaccine, extemporaneously mixed with GSK Biologicals' Tritanrix™-HepB, as compared to GSK Biologicals' Hiberix™ vaccine, extemporaneously mixed with GSK Biologicals' Tritanrix™-HepB, when administered intramuscularly in infants at 6, 10 and 14 weeks of age.

Amendment 1
05 June 2003

Coordinating Author: N. Bülow

Rationale/background for changes:

This amendment includes several changes / additional information as requested by several ethics committees who reviewed the protocol:

Change in the nomenclature of study alias: DTPwHB/HIBMenACTT instead of Hib-MenAC-TT. Details for the WHO monitor and SAE contact were added.

Information were added to

- justify why 1/5 dose of Mencevax™ AC will be administered
- further elaborate the study rationale and study site conditions
- stress that any lack of efficacy needs to be reported to the DSMB
- specify the distance between the subjects' compounds and the health facilities and the duration of each study visit

An additional informed consent is mentioned that has to be signed by any parent/guardian accompanying the child (instead of the mother, at visits subsequent to the first visit) prior to providing any saliva sample. Details of the timepoint of study start were removed and two references were added. A potential change of PATH' corporate structure was taken into account.

Appendix H (Meningococcal carriage and mucosal immunity to *Neisseria meningitidis* in the African meningitis belt) was restructured and additional information on e.g. sample size and the power of the ancillary study were included. A supplement to the ancillary study was added describing the procedure of taking throat swabs.

The following items were amended on 05 June 2003:

1st page: **Study Vaccine**

GlaxoSmithKline (GSK) Biologicals'*Haemophilus influenzae* type b-meningococcal AC-tetanus toxoid (Hib-MenAC) vaccine ***(as a mixed combination with Tritanrix™-HepB)***

Throughout the protocol the study alias Hib-MenAC-TT has been replaced by DTPwHB/HIBMenACTT.

On page 2 and in the abbreviation list a potential change of PATH' corporate structure was taken into account by adding a footnote/Note:

≠PATH currently expects to form a limited liability company (the "LLC") to pursue the mission of, and to hold all of PATH's interest in, the Meningitis Vaccine Project. PATH will be the sole member and the sole manager of the LLC. In such case, the LLC, rather than PATH, will act as the co-sponsor of this protocol.

Details of the WHO monitor and Study Contact for Reporting SAEs were added on page 4 (prior to "Sponsors"):

WHO Monitor

Dr.Lawrence Kweku Yamuah
Centre for Measurement and Information in Medicine
City University
Northampton Square
London, EC1V 0HB
United Kingdom
Tel: (+ 44) 20 7040 8371
Fax: (+ 44) 20 7040 8371
email: L.K.Yamuah@city.ac.uk
yamuahlawrence_kweku@hotmail.com

WHO Study Contact for Reporting SAEs
Yuppaporn Wattanagoon
M121 WHO/HTP/IVR/POP
Avenue Appia 20
CH-1211 GENEVE 27
Switzerland
Tel. +41 22 791 15 01
Email: wattanagoony@who.int

Sponsor Information

Study Monitor WHO

~~A WHO monitor will be identified prior to the start of the study.~~

Dr.Lawrence Kweku Yamuah
Centre for Measurement and Information in Medicine
City University
Northampton Square
London, EC1V 0HB
United Kingdom
Tel: (+ 44) 20 7040 8371
Fax: (+ 44) 20 7040 8371
email: L.K.Yamuah@city.ac.uk
yamuahlawrence_kweku@hotmail.com

Study Contact for Reporting of a Serious Adverse Event for WHO

~~TBD. The name, address, phone and fax numbers will be provided prior to study start.~~

Yuppaporn Wattanagoon
M121 WHO/HTP/IVR/POP
Avenue Appia 20
CH-1211 GENEVE 27
Switzerland
Tel. +41 22 791 15 01
Email: wattanagoony@who.int

Section 1.1 Background (One paragraph was added at the end of this section)

The immunogenicity of meningococcal conjugate vaccines in infants is best shown by measuring bactericidal antibodies one month after primary vaccination. Demonstrating that meningococcal serogroup A and C conjugate vaccines prime for immune memory is an important mechanism to support the long-term protection effect of these vaccines. Such priming for memory was shown for the meningococcal serogroup C conjugate vaccine [17] and for a preliminary meningococcal serogroup A+C conjugate candidate vaccine evaluated in the United Kingdom [18]. However, in another trial in the Gambia, priming for memory of the A+C conjugate vaccine was less convincing [15]. Priming for memory was demonstrated by administering a small dose (10 µg) of unconjugated polysaccharide C six to twelve months after priming. The dose of 10 µg polysaccharide was selected after three adverse events (allergic reactions) had been reported in 10 subjects initially vaccinated with the full polysaccharide vaccine (50 µg/polysaccharide). No further allergic reactions

were reported with the reduced dose of 10 μg [18].

Synopsis and Section 1.3 Rationale for the study (one paragraph was added at the end of this section)

The study will evaluate the immunogenicity induced by three doses of the vaccine given at 6, 10 and 14 weeks of age by measuring the immune response one month post-vaccination to ensure the vaccine is effective in the youngest children when the disease burden is highest. In order to assess whether the vaccine has induced long-term protection, the persistence of the immune response will be measured when the child is one year of age, and a small dose of plain polysaccharide (1/5th of a dose of Mencevax™ AC, i.e. 10μg/polysaccharide) will be given to evaluate whether immune memory was induced by priming.

Section 1.4 Study site

The following paragraphs were added just before the sub-section "Health indicators":

Coordination with the EPI

The vaccine trial will be conducted at three EPI clinics in the Kassena-Nankana district where routine immunization is taking place:

- ***KNE-Health Centre (serving the East sub district)***
- ***Biu Health Centre (serving the South sub district)***
- ***War Memorial Hospital (serving Navrongo central)***

All vaccinees in the EPI system have the so-called "Growth Monitoring Cards" on which the vaccines they receive are recorded. The subjects will be recruited at the time they are to receive the routine DTP, Hib and HepB vaccines (at 6 weeks of age). The vaccines given will be recorded on a specific immunization card (yellow card) and also on the Growth Monitoring Cards, i.e. the name of the study vaccine will be recorded in the box where DTP, Hib and HepB vaccines are usually recorded. Therefore, a duplication of vaccination is prevented as the Growth Monitoring Cards are always brought to the EPI clinics by the parents/guardians.

Section 3 Study Design Overview

In the flow chart and in the 3rd bullet point pluriel was used for measles and yellow fever vaccines.

Flow chart:

- Measles & Yellow fever vaccine***s*** @ 9 months of age
- At Visit 5, Month 7.5 Measles/ Yellow Fever Vaccine***s***

- To comply with the Ghanean Immunisation Calendar, at 9 months of age, all subjects will receive measles and yellow fever vaccine***s.***

Synopsis and Section 3 Study Design Overview (one bullet point was deleted)

- ~~Study start: The enrolment will preferably start at the onset of the rainy season (June) and should be completed prior to the onset of the dry season (October).~~

Section 5.1.3 Data Safety Monitoring Board (DSMB) (2nd bullet point and 2nd sentence in paragraph following "Role of Local Safety Monitor")

- systemic grade 3 adverse events (AEs) related to vaccination ~~and~~**,** serious adverse events ***and adverse events suggesting a lack of efficacy of the vaccine*** occurring during the study (transmitted through the Local Safety Monitor)

All systemic grade 3 AEs probably or suspected to be related to vaccination**,** ~~and~~ all SAEs ***and all AEs suggesting a lack of efficacy of the vaccine*** will be reported to him/her in his/her capacity as a liaison to the DSMB.

Section 5.2 General study aspects (one paragraph was added at the beginning of this section)

The maximum distance between a subject's compound and the nearest health facility will be 1 km. Each visit to the clinic for vaccination will last approximately 4 hours; each home visit by a field worker will last about 15 minutes.

Section 5.3 Outline of study procedures

The following footnote was added to the informed consent row in the table:

# any parent/guardian accompanying the child instead of the mother at visits subsequent to the first visit has to sign an Informed Consent prior to providing any saliva sample

Section 5.4 Detailed description of study stages/visits

The following note was added prior to the detailed description of Visits 3, 5, 6 and 7:

Note: ***Any parent/guardian accompanying the child instead of the mother at visits subsequent to the first visit has to sign an Informed Consent prior to providing any saliva sample.***

Section 6.5 Replacement of unusable vaccine doses (2nd paragraph)

In addition to the vaccine doses provided for the planned number of subjects, ***at least*** 5% additional doses will be supplied…

Section 8.3. Lack of efficacy (one sentence was added at the end of this section)

Any case of diphtheria, tetanus, pertussis, hepatitis B, invasive diseases due to Haemophilus influenzae type b, measles, yellow fever or Neisseria meningitidis of serogroup A and C will be reported to the Data and Safety Monitoring Board (DSMB).

In Section 8.7 Follow-up of adverse events and serious adverse events and assessment of outcome and *Section 8.8.1 Time frames for submitting serious adverse event reports to GSK Biologicals and PATH* the term "when appointed" was deleted for the WHO monitor as she is now appointed.

Section 8.7 paragraph written in bold: **All SAEs will be reported within 24 hours by the investigator to GSK Biologicals, to the PATH Focal Point, to the local safety monitor and~~, when appointed,~~ to the WHO monitor.**

Section 8.8.1, 2nd paragraph: In addition to the direct reporting of any SAE and any subsequent information by the investigator or its designate to GSK Biologicals and PATH, the investigator will also report the SAE and subsequent information to the local safety monitor for transmission to the DSMB (see section

5.1.3) and~~, when appointed,~~ to the WHO monitor.

Section 8.8.2 Completion and transmission of serious adverse event reports to GSK Biologicals and PATH

Details for the WHO study monitor were added:

~~TBD. The name, address, phone and fax numbers will be provided prior to study start.~~

Study Contact for SAE Reporting at WHO
Yuppaporn Wattanagoon
M121 WHO/HTP/IVR/POP
Avenue Appia 20
CH-1211 GENEVE 27
Switzerland
Tel. +41 22 791 15 01
Email: wattanagoony@who.int

Section References (two references were added)

[17] ***Richmond P, Borrow R, Miller E, Clarck S, Sadler F, Fox A, Begg N, Morris R, Cartwright K. Meningococcal serogroup C conjugate vaccine is immunogenic in infancy and primes for memory. Journal Infectious Diseases 1999; 179: 1569-72.***

[18] ***Borrow R, Fox AJ, Richmond PC, Clarck S, Sadler F, Findlow J, Morris R, Begg NT, Cartwright K. Induction of immunological memory in UK infants by a meningococcal A/C conjugate vaccine. Epidemiol. Infect 2000; 124: 427-432.***

Appencix C: Overview of the Recruitment Plan

The 1st bullet point of this section was amended:

- All subjects should be enrolled within a period of 5 months ~~(preferably June to October~~).

Appendix D: Handling of Biological Samples Collected by the Investigator and Appendix E: Shipment of Biological Samples

The Fax number at the end of each Appendix was corrected:

Fax: +32-2-656 ~~6052~~***9144***

Appendix G: Vaccine supplies, packaging and accountability (paragraph after bullet point list)

An additional ***quantity of at least*** 5% of Tritanrix™-HepB and Hiberix™ will be supplied for replacement in case of breakage, bad storage conditions or any other reason that would make the vaccine unusable (i.e., given by mistake to another subject).

Appendix H: Meningococcal carriage and mucosal immunity to Neisseria meningitidis in the African meningitis belt

The entire section was restructured and additional information on e.g. sample size and power were included. In addition, a supplement was added describing the procedure of taking throat swabs.

GlaxoSmithKline Biologicals
Rue de l'Institut 89
1330 Rixensart, Belgium

Protocol Amendment Approval

Study Drug

Hib-MenAC

Protocol Number

759346/009 (DTPwHB/HIBMenACTT009)

Amended 5 June 2003 and 02 October 2003)

Protocol Title: A phase II, double blind, randomized, controlled study to evaluate the immunogenicity, reactogenicity and safety of GlaxoSmithKline (GSK) Biologicals' Hib-MenAC vaccine, extemporaneously mixed with GSK Biologicals' Tritanrix™-HepB, as compared to GSK Biologicals' Hiberix™ vaccine, extemporaneously mixed with GSK Biologicals' Tritanrix™-HepB, when administered intramuscularly in infants at 6, 10 and 14 weeks of age.

Amendment 2
02 October 2003

Coordinating Author: Sheila Woods

Rationale/background for changes:

The formulation of the Tritanrix™-HepB/Hib-MenAC vaccine which will be used in this and other Phase II studies has now been selected following the analysis of data from the separate dose-range study which evaluated three different formulations of the Tritanrix™-HepB/Hib-MenAC vaccine administered in infants at 6, 10 and 14 weeks of age.

As the presentation, and preparation, of the selected formulation of vaccine is different to the Tritanrix™-HepB/Hiberix™ control vaccine, information regarding the practical procedures to be implemented to ensure the blinding of the study (preparation and administration of the vaccines) as well as the details of the content and presentation of the vaccine have been added in this amendment.

The immunogenicity analysis of the dose-range study has highlighted the importance of having an accurate picture of the pre-vaccination maternal antibody levels in the infants (especially hepatitis B, diphtheria, pertussis and meningococcal serogroup A and C antibodies) as it appears that the response to the vaccine (especially to the hepatitis B, diphtheria, pertussis and meningococcal serogroups A and C antigens) can be affected by these levels. It has therefore been decided to add an additional blood sampling from the infants in this study at the

pre-vaccination timepoint and to drop the planned blood sampling from the mother. The protocol has been amended accordingly, including changes to the objectives and endpoints where relevant. Furthermore since the mother is no longer required to accompany the child for the first visit, the protocol (and the informed consent) has been changed to allow another parent or guardian to provide informed consent.

As it is possible that the collection of the additional blood sample from the infants in the study may lead to an increased rate of withdrawal/drop-out of subjects from the study, the sample size has been increased from 260(130/group) to 280 (140/group) to ensure that the required number of evaluable subjects is reached.

As the committee that will be set up to oversee the ethical and safety aspects of the study conduct will be an independent organisation, it will now be called the Independent Data Monitoring Committee (IDMC) rather than the Data Safety Monitoring Board (DSMB).

Appendix H (Meningococcal carriage and mucosal immunity to *Neisseria meningitidis* in the African meningitis belt) has been restructured and additional information on the sample size included so that it complies with PATH’s guidelines for protocols.

The following items were amended on 02 October 2003:

GSK Biologicals has changed their monitoring system from CPMS to GSK’s clinical trials tracking tool, eTrack, with study identification by eTrack study number and abbreviated title. Therefore, throughout the protocol, the CPMS number and alias: 759346/009 (DTPwHB/HIBMenACTT-009) has been changed to the eTrack study number and abbreviated title: 759346/009 (DTPwHB/HIBMenACTT009); eTrack has also been added to the Glossary of Terms.

Sponsor Information and/or Title pages
Study Monitors: Cornelia Bevilacqua’s contact details were amended, contact details for the WHO monitor Dr. Lawrence Kweku Yamuah amended, WHO Study Contact for Reporting SAEs was changed from Yuppaporn Wattanagoon to Dr. Zarifah Reed and an additional collaborator from the London School of Hygiene and Tropical Medicine (Dr. Daniel Chandramohan) was added:

London School of Hygiene and Tropical Medicine

**Collaborating persons:**	Prof. Brian Greenwood<br>***Dr. Daniel Chandramohan***
**WHO Monitor**<br>***Dr. Lawrence Kweku Yamuah***<br>~~*Centre for Measurement and Information in Medicine*~~<br>~~*City University*~~<br>~~*Northampton Square*~~<br>~~*London, EC1V 0HB*~~<br>~~*United Kingdom*~~<br>~~*Tel: (+ 44) 20 7040 8371*~~<br>~~*Fax: (+ 44) 20 7040 8371*~~<br>***Email:*** ~~*L.K.Yamuah@city.ac.uk*~~<br>***yamuahlawrence_kweku@hotmail.com***	***Dr. Lawrence Kweku Yamuah***<br>***Post-Doctoral Researcher / Data Manager***<br>***Armauer Hansen Research Institute (AHRI)***<br>***P.O. Box 1005***<br>***Addis Ababa***<br>***Ethiopia***<br>***Tel: + 251 1 71 02 88 (work)***<br>***Tel: + 251 1 71 17 04 (home)***<br>***Fax: + 251 1 71 13 90***<br>***Email:***<br>yamuahlawrence_kweku@hotmail.com
**WHO Study Contact for Reporting SAEs**<br>~~*Yuppaporn Wattanagoon*~~<br>~~*M121 WHO/HTP/IVR/POP*~~<br>~~*Avenue Appia 20*~~<br>~~*CH-1211 GENEVE 27*~~<br>~~*Switzerland*~~<br>~~*Tel. +41 22 791 15 01*~~<br>~~*Email: wattanagoony@who.int*~~	***Dr. Zarifah Reed***<br>WHO/**VAB-I**/IVR/POP<br>***20 Avenue Appia***<br>***CH-1211 GENEVE 27***<br>***Switzerland***<br>***Tel. +41 22 791 4760***<br>***Fax. +41 22 791 4860***<br>***Email: reedz@who.int***
**Study Monitors**<br>Cornelia Bevilacqua<br>~~Central Study Coordinator,~~<br>~~GlaxoSmithKline Biologicals,~~<br>~~89, Rue de l'Institut, 1330 Rixensart, Belgium~~<br>~~Tel: + 32 2 656.77.70~~<br>~~Fax: + 32 2 656.81.33~~<br>~~Mobile: + 33 (0) 685 25 24 69~~<br>~~e-mail:~~ cornelia.bevilacqua@gskbio~~.com~~	Cornelia Bevilacqua<br>***'La Fédarié Haute'***<br>***81440 Vénès***<br>***France***<br>***Tel: + 33 563 75 92 66***<br>***Fax: + 33 563 75 92 66***<br>***Mobile phone: + 33 685 25 24 69***<br>***e-mail: cornelia_bevilacqua@yahoo.fr***

Sponsor Information

Study contact for Emergency Code Break

Dr. John Williams (who will forward information to ~~DSMB~~ ***IDMC*** chairman)

Section 1 Introduction (3rd paragraph, 2nd sentence); Section 1.3 Study rationale and Synopsis (2nd sentence)

Except for ~~an~~ ***outbreaks*** of serogroup W-135 that spread in Burkina Faso in 2002 ***and 2003***, serogroup A and to a lesser extent serogroup C are largely responsible for *N. meningitidis* epidemics in African countries.

Section1.1 Background (5th paragraph, 4th sentence amended including addition of underlined text)

However, in another trial in the Gambia, strong memory to the C component was induced but evidence of memory induction for the A component ~~priming for memory of the A+C conjugate vaccine~~ was less convincing.

Section 1.2 GSK Biologicals' combined… Hib MenAC) conjugated vaccine (4th paragraph amended to include results of the feasibility study)

~~The new combined Hib-MenAC (using TT as carrier protein) extemporaneously mixed with Tritanrix™-HepB was shown to be safe and immunogenic in preclinical studies. A phase II dose-range study in infants is ongoing outside the African continent and preliminary data will be available prior to starting this study.~~

In the first three-dose primary vaccination study performed in the Philippines, three formulations of the combined Hib-Men AC vaccine (using TT as carrier protein) each extemporaneously mixed with Tritanrix™-HepB were evaluated and the Tritanrix™-HepB/Hib-MenAC combination using the Hib-MenAC formulation with a dosage of 2.5 µg of each of the MenA, MenC and Hib conjugate antigens was selected for further clinical development: the selected formulation was well tolerated; it induced similar antibody levels against the diphtheria, tetanus, pertussis, hepatitis B, Hib and meningococcal serogroup C antigens as the benchmark vaccines (Tritanrix™-HepB/Hiberix™ and Meningitec™) and 97.7 % subjects had meningococcal serogroup A serum bactericidal antibody titres of at least 1:8. See Appendix I for a summary of clinical data of this study, including a preliminary report on the Serious Adverse Events reported during that study

(5th paragraph amended as follows)

Pre-clinical and clinical data generated with GSK Biologicals' meningococcal conjugate vaccines are available in the *Neisseria meningitidis* conjugate vaccines Investigator Brochure (*Neisseria meningitidis* A, C and Y conjugate vaccines, 3rd edition, September 2001). ***An updated edition of this Investigator Brochure (which will include the data presented in Appendix I) is under preparation.***

Section 1.3 Study Rationale and Synopsis (4th paragraph amended to include results of the feasibility study)

The new Tritanrix™-HepB/Hib-MenAC vaccine ~~is being~~ ***has been*** evaluated in a feasibility trial ~~outside Africa~~ in the Philippines. ***The combination using the Hib-MenAC formulation with a dosage of 2.5 µg of each of the MenA, MenC and Hib conjugate antigens has been selected for further clinical development: the selected formulation was well tolerated; it induced similar antibody levels against the diphtheria, tetanus, pertussis, hepatitis B, Hib and meningococcal serogroup C antigens as the benchmark vaccines (Tritanrix™-HepB/Hiberix™ and Meningitec™) and 97.7 % subjects had meningococcal serogroup A serum bactericidal antibody titres of at least 1:8.*** ~~Once~~ ***As*** feasibility of the new vaccine ~~is~~ ***has now been*** shown, the present study plans to evaluate the use of GSK Biologicals' Tritanrix™-HepB/Hib-MenAC vaccine in a high endemicity region in Africa.

Section 2.2 Secondary objectives and Synopsis (Deletion of the first bullet**)**

~~Just prior to the administration of the first vaccine dose, to assess the serum bactericidal antibodies against *N. meningitidis* serogroups A and C in the mother of the child as an indirect measurement of the prevaccination status of her infant.~~

Section 3 Study design overview

In the flowchart, the number of subjects enrolled was amended from 130 per group to 140 per group and the Visit 1 "Pre blood sample in the child's mother" was changed to Blood sampling [of the child].

Section 3 Study design overview and Synopsis (Note/bullet point regarding OPV amended and 3 further bullet points amended and one deleted)

BCG and OPV vaccines can be given at birth (or ***up to*** at least 2 weeks before the subject's first visit) according to the nationally recommended immunization

schedule. OPV can also be given at 6, 10, 14 weeks of age concomitantly with the study vaccines ***or at any time during the study, whichever is more appropriate.***

Experimental design: 2 parallel groups of ~~130~~ ***140*** subjects each to receive either of the following vaccines:

As the control group will not be properly immunised with 1/5th of a dose of Mencevax™ AC, ~~at study end, the control group Tritanrix™-HepB/Hiberix™ will be offered one dose of Mencevax™ ACWY in order to protect the children against the meningococcal diseases~~ ***after study end, one dose of Mencevax™ ACWY will be offered to the Tritanrix™-HepB/Hiberix™ control group to protect them against meningococcal diseases of these serogroups: Mencevax™ ACWY will be given when the child is two years of age, or, if a meningitis epidemic occurs, earlier in the second year of life.***

- For all subjects: ~~3~~ ***4*** blood samples (3.5 ml each):
  - ***immediately before the first dose of the primary vaccination course***
  - one month after the third dose of the primary vaccination course
  - just prior to administration of the plain polysaccharides of serogroups A and C
  - one month after the administration of the plain polysaccharides of serogroups A and C.

~~For the mothers of all subjects, just prior to the administration of the first vaccine dose to their infants: one blood sample (3.5 ml).~~

Section 4.1 Number of subjects and Synopsis (1st and 2nd paragraphs)

Target enrolment will be ~~260~~ ***280*** healthy male and female infants (~~130~~ ***140*** subjects per group) to provide 220 evaluable subjects (110 per group) for the immunogenicity analysis. The study will be conducted at the Navrongo Health Research Center in Ghana (War memorial Hospital and ~~several~~ ***2*** health centres of Navrongo health district).

Enrolment will be terminated when ~~260~~ ***280*** subjects have been enrolled

Section 4.2 Inclusion criteria (One bullet point amended)

Written informed consent obtained from the ~~mother~~ ***parent or guardian*** of the subject.

Section 4.5 Contraindications **(contraindications for Hiberix™ added)**

The Hiberix™ vaccine should not be administered to subjects with known hypersensitivity to any component of the vaccine, or to subjects having shown signs of hypersensitivity after previous administration of Hib vaccines.

Section 5.1.3 **(**section heading changed as follows and throughout section DSMB changed to IDMC: 17 changes)

5.1.3 ~~Data Safety Monitoring Board (DSMB)~~ *Independent Data Monitoring Committee (IDMC)*

(Composition of IDMC: 2nd paragraph amended as shown)

The Charter (terms of references) ~~of the Terms of reference (operating procedures)~~ of the ***IDMC*** will be written after the first meeting of the ***IDMC*** members, prior to study start.

Role of the ***IDMC*** (2nd paragraph)

The ***IDMC*** will confer before the initiation of the study (pre-initiation review), during the study proper and at the close out of the study to review any relevant safety data ~~and to review and approve the Report and Analysis Plan (RAP), where applicable~~ ***; after study end, the IDMC will have a first assessment report***…

(**Role of Local Safety Monitor**: Second and last bullet point amended as shown)

reviewing blinded reactogenicity data~~ after each dose~~ ***according to IDMC charter*** and discussing with ***IDMC*** if necessary

Unblinding a subject if deemed necessary***, upon request of the investigator/physician in charge of the subject,*** to allow for adequate treatment.

Section 5.2 General study aspects (1st paragraph and 2nd, 4th and 5th sentences of last paragraph)

The maximum distance between a subject's compound and the nearest health facility will be ~~1 km~~ 10 km. Each visit to the clinic for vaccination will last approximately 4 hours; each home visit by a field worker will last about 15 minutes.

PATH and GSK Biologicals are not sponsor of this ancillary study but the protocol of this ancillary study is attached as an Appendix to this protocol (refer Appendix H for further details) for operational reasons (only one subject

information sheet will be written and ~~only~~ one informed consent will be submitted to the approval of the parents/guardians of the child, ***however, any parent/guardian accompanying the child at visits subsequent to the first visit who has not already signed an Informed Consent for this study will also have to sign an Informed Consent prior to providing any throat swab or saliva sample***). This ancillary study will be reported separately by the investigators of the ancillary study. This ancillary study requires collection of throat swabs and saliva samples from the ~~mothers~~ ***parent/guardian*** and infant~~s~~ prior to the first dose (6-8 weeks) and at 10 weeks, 18 weeks, 9 months and 12 months of age. The collection of ***throat swab and*** saliva samples will be detailed in the study procedures (section 5. 3) and the subject information sheet.

Section 5.3 Outline of study procedures

In Table 5-1, as mother's blood sampling replaced by child's blood sampling at Visit 1, corresponding footnote amended to refer to serology section:

*~~ from the mother only~~ ***a minimum of 2 ml may be taken at prevaccination timepoint. See section 5.5.2;***

3rd footnote amended by addition of underlined text as follows :

# any parent/guardian accompanying the child ~~instead of the mother~~ at visits subsequent to the first visit <u>who has not already signed an Informed Consent for this study will have</u> ~~has~~ to sign an Informed Consent prior to providing any throat swab or saliva sample.

Note at end of footnotes (2nd sentence amended):
OPV vaccine can be administered to the subjects at 6, 10 and 14 weeks of age ***concomitantly with the study vaccines or at any time during the study, whichever is more appropriate.***

Section 5.4 Detailed description of study stages/visits
(**Visit 1** change of the following 2 bullet points, regarding informed consent and blood sampling)

Written informed consent from the subject's ~~mother~~ ***parent/guardian***.

~~Collection of 3.5 ml of whole blood from the mother.~~

~~Collection of blood for serology: a minimum of 3.5 ml of whole blood to provide a minimum of 1.5 ml of serum according to instructions in Appendix D.~~

Collection of blood for serology: it is understood that collection of blood in infants aged 6 weeks may be technically difficult. Therefore a minimum of 2.0 ml of whole blood, which is the minimum volume of blood that will allow the essential serology to be undertaken, has to be taken according to

instructions in Appendix D. However, whenever possible a 3.5 ml sample should be collected to provide the 1.5 ml sample of serum that is needed for full serological assessment of the response to the vaccine and for validation assays. Section 5.5.2.
Technically skilled and trained individuals will draw blood to avoid unnecessary pain and technical failure as much as possible.

(**Visits 1, 2, 3 and 6:** Amendment of the following bullet point)

The field worker will be given a diary card and will visit the subjects at their home to record, on the day of vaccine injection ***(day 0)*** and during the 3 subsequent days ***(days 1-3)*** or up to resolution of the symptoms, whichever is longer, the subject's body temperature and any local (at the injection site) or general adverse events ~~occurring on the day of vaccination (day 0) and during the 3 subsequent days (days 1-3)~~ (see section 8.5.1).

(**Visits 1, 2, 3, 4 and 6:** Amendment of the last bullet point)

The subjects' parents/guardians will be instructed to contact the investigator***/study contact person at the health centre*** immediately should the subject manifest any signs or symptoms they perceive as serious.

(**Visit 2** amendment of 1st bullet point and N.B.)

Provision of definitive study ID card (plastified ID card including picture of parent/guardian ~~(mother)~~ and infant).

N.B. The parent/guardian ~~(mother)~~ of the study subject will be asked to present the study ID card <u>every time</u> they attend the hospital / health centers.

(**Visits 2, 4, 5, and 6:** amendment of Note by deletions and addition of underlined text)

Note: ***Any parent/guardian accompanying the child ~~instead of the mother~~ at visits subsequent to the first visit <u>who has not already signed an Informed Consent for this study will have</u> ~~has~~ to sign an Informed Consent prior to providing any saliva sample.***

(**Visit 7:** amendment of last bullet point)

After study end, one dose Mencevax™ ACWY will be offered to the Tritanrix™-HepB/Hiberix™ control group to protect them against meningococcal diseases

of these serogroups: ~~The~~ Mencevax™ ACWY will be given when the child is two years of age or, if a meningitis epidemic occurs, ~~already~~ ***earlier*** in the second year of life.

(**Visit 4, 6 and 7:** amendment of following bullet point)

Collection of blood for serology: a minimum of 3.5 ml of whole blood to provide a minimum of 1.5 ml of serum according to instructions in Appendix B. ***Technically skilled and trained individuals will draw blood to avoid unnecessary pain and technical failure as much as possible..***

Section 5.5.2 Laboratory assays (2nd paragraph, points 1 and 4 and Table 5-3)

2nd paragraph amended as follows:
For all subjects, a 3.5 ml sample of whole venous blood will be collected at visits ***1,*** 4, 6 and 7 using tubes with serum separator. ***Since the investigator has suggested that the collection of this amount of blood at Visit 1 may be technically difficult in such small infants, the quantity collected at this visit can be reduced to 2.0 ml, as a strict minimum, however this will not allow for any validation/retesting etc that may be required when the testing is performed.*** ~~In addition, in all mothers, a 3.5 ml of whole venous blood will be collected at Visit 1.~~

The following was added to the end of point 1:

More recently, efficacy data from postlicensure surveillance has been shown to validate the SBA-MenC 1:8 cut-off as a serological correlate of protection for meningococcal serogroup C conjugate vaccines [29].

The following was added to the end of point 4:

Post-vaccination serum samples with ELISA anti-D antibody concentrations <0.1 IU/ml will be re-tested using a VERO-cell neutralization assay [33, 34]. The cut-off of the VERO-cell assay is 0.016 IU/mL.

Table 5-3: Laboratory Assays amended as follows:

Antigen	Assay method	Test Kit/ Manufacturer	Assay unit	Assay cut-off	Laboratory
SBA-MenA	Bactericidal assay	in house	dilution for 50% killing	1:8	Rixensart
SBA-MenC	Bactericidal assay	in house	dilution for 50% killing	1:8	Rixensart
PRP	ELISA	in-house	µg/ml	0.15	Rixensart*
PSA	ELISA	in-house	µg/ml	0.3	Rixensart
PSC	ELISA	in-house	µg/ml	0.3	Rixensart
Diphtheria	ELISA	in-house	IU/ml	0.1	Rixensart*
Tetanus	ELISA	in-house	IU/ml	0.1	Rixensart*
BP	ELISA	commercial	EL.U/ml	15	Rixensart*
HBs	ELISA	commercial	mIU/ml	10	Rixensart*
Diphtheria	***Vero-cell***	***in house***	***IU/ml***	***0.016***	***Rixensart***

*or in a validated laboratory designated by GlaxoSmithKline Biologicals

Section 5.5.3 Serology plan (1st, 2nd and 3rd paragraphs amended)

Pre-vaccination (Day 0) serum samples will be tested in all subjects for antibodies against SBA-MenA, SBA-MenC, anti-PSA, anti-PSC, diphtheria toxoid, pertussis antigen and recombinant DNA hepatitis B surface antigen (no pre-vaccination testing of anti-TT and anti-PRP is foreseen as immunogenicity results of the previous clinical study were highest for these two antibodies).

Post-vaccination III and pre-booster serum samples will be tested in all subjects for antibodies against all vaccine antigens (SBA-MenA, SBA-MenC, anti-PRP, anti-PSA, anti-PSC, diphtheria and tetanus toxoids, pertussis antigen and recombinant DNA hepatitis B surface antigen).

The post-booster serum sample taken from all children ~~**and the prevaccination serum sample taken from their mothers**~~ will be tested for SBA-MenA and SBA MenC, anti-PSA and anti-PSC antibodies.

In case of insufficient blood sample volume to perform assays for all antibodies, they will be analyzed according to the following priority ranking:

- SBA-MenA
- SBA-MenC
- anti-PRP ***(only for Post vacc III and pre-booster)***
- ~~anti-PSA~~
- ~~anti-PSC~~
- ~~anti-diphtheria, anti-tetanus, anti-BP, anti-HBs~~
- ***anti-diphtheria (Vero-cell assay only when required for Post vacc III and Pre-booster)***
- ***anti-HBs***
- ***anti-tetanus, (only for Post vacc III and pre-booster)***
- ***anti-BP***
- ***anti-PSA***
- ***anti-PSC***

Section 5.5.4 Endpoints for suboptimal response (suboptimal response for anti-diphtheria concentration changed to Vero-cell cut-off and last sentence amended)

anti-diphtheria concentration ~~<0.1IU/ml~~ ***< 0.016 IU/ml (Vero-cell assay)***

~~The~~ Mencevax™ ACWY will be given when the child is two years of age or, if a meningitis epidemic occurs, ~~already~~ ***earlier*** in the second year of life.

Section 6.1.1 GSK Biologicals' Haemophilus influenzae type b meningococcal AC-TT (Hib-MenAC) conjugate vaccine (section amended as follows)

One lot of the vaccine will be used. The vaccine will be supplied as a white freeze dried pellet in monodose vials to be reconstituted with Tritanrix™-HepB before use. ***Each vial of the Hib-MenAC (5/5/5) vaccine needs to be reconstituted with 2 vials of the Tritanrix™-HepB before use (0.5 ml per vial). Only half of the dose (i.e. 0.5 ml will be administered. For details of the dose dilution see Section 6.1.7.*** One dose (0.5 ml) of the reconstituted vaccine contains the following components:

Haemophilus influenzae type b capsular polysaccharide (PRP) conjugated to tetanus toxoid	:	~~TBD~~ ***2.5 µg***
Neisseria meningitidis A capsular polysaccharide conjugated to tetanus toxoid	:	~~TBD~~***2.5 µg***
Neisseria meningitidis C capsular polysaccharide conjugated to tetanus toxoid	:	~~TBD~~***2.5 µg***

~~Note: The dose of each component (2.5 µg or 5 µg for each polysaccharide) will be determined in a separate dose-range study, results of which will be available prior to study start.~~

Section 6.1.3: GSK Biologicals' Haemophilus influenzae type b conjugate vaccine: Hiberix™ (1st sentence)

The vaccine will be supplied as a white freeze-dried pellet in monodose vials to be reconstituted with *one 0.5 ml vial* ***of*** **Tritanrix™-HepB before use.**

Section 6.1.7 Extemporaneous mixing of Tritanrix™-HepB and Hib-MenAC vaccines or Hiberix™
New subheading "**Tritanrix™-HepB and Hiberix™"** added and 2nd sentence amended:
The full content of the Tritanrix™-HepB vaccine vial should be extracted and injected into the vial containing the lyophilized ~~Hib-MenAC or~~ Hiberix™ vaccine~~s~~.

Additional subsection with subheading added as follows:

Tritanrix™-HepB and Hib-MenAC : dose dilution to provide Hib-MenAC (2.5/2.5/5.5)

The Hib-MenAC (2.5/2.5/2.5) formulation containing 2.5 µg of each polysaccharide will be used in this study. This will be obtained by dilution of the supplied Hib-MenAC (5/5/5) vaccine that contains 5µg of each polysaccharide. Each vial of the Hib-MenAC (5/5/5) vaccine needs to be reconstituted with 2 vials of the Tritanrix™-HepB before use.

The liquid Tritanrix™-HepB vaccine should always be shaken before use. The full content of two monodose vials of Tritanrix™-HepB vaccine should be extracted and injected into the vial containing the lyophilized Hib-MenAC (5/5/5) vaccine. The vial should be agitated until the lyophilized vaccine pellet has completely dissolved. The mixed vaccines will appear white. The reconstituted mixed vaccines should be used promptly after reconstitution (within 30 minutes):

- ***withdraw one dose of 0.5 ml of the mixed vaccines from the vial;***
- ***a new needle should be used for injection;***

One dose (0.5 ml) of the mixed vaccines should be administered by intramuscular injection into the anterolateral quadrant of the left thigh.

Note: The reconstituted vaccine remaining in the vial after 1 dose (0.5 ml) has been withdrawn is to be retained by the investigator for purposes of vaccine accountability and should not be reused. See Appendix G for more details.

Section 6.3 Treatment allocation and randomization (1st sentence)
The target sample size is ~~260~~ ***280*** enrolled subjects (220 evaluable for immunogenicity analysis, with 110 subjects in each group).

Section 6.4 Method of blinding and breaking the study blind (1st paragraph)

The study will be double blind. ***The study vaccine and the control vaccine will have the same whitish appearance after reconstitution. However, since the vaccines used in this study are different in their packaging volumes (Tritanrix™-HepB and Hiberix™ = 1 vial of 0.5ml of each vaccine, Tritanrix™-HepB and Hib-MenAC = 2 vials of 0.5 ml of Tritanrix™-HepB and 1 vial of 0.5 ml of Hib-MenAC), special precautions will be taken to ensure blinding. For each vaccination during the course of the study, a Syringe Filler and Vaccinator will work as a vaccination team: the Syringe Filler will prepare the vaccine for administration to a specific subject and the Vaccinator will administer the vaccine to each subject, according to the investigator's SOP. To ensure blinding, a vaccination team will perform no other function in the study. The subject's parent/guardian as well as those responsible for the evaluation of safety or immunogenicity study parameters will all be unaware which vaccine preparation was administered to a particular subject.*** ~~The subject's parents/guardians, the investigator and the study personnel will be blinded to the vaccines to be administered.~~

Section 6.5 Replacement of unusable vaccine doses (addition of underlined text to 1st sentence of 2nd paragraph

In addition to the vaccine doses provided for the planned number of subjects, ***at least*** 5% additional doses of ***Tritanrix™-HepB and Hiberix™*** will be supplied.

*Section 8 Adverse Events**,** ~~and~~ Serious Adverse Events* ***and vaccine failure***

Section 8.3 Lack of efficacy (Last sentence amended)

Any case of diphtheria, tetanus, pertussis, hepatitis B, invasive diseases due to Haemophilus influenzae type b, measles, yellow fever or Neisseria meningitidis of serogroup A and C will be reported to the ~~Data and Safety Monitoring Board (DSMB)~~ Independent Data Monitoring Committee (IDMC).

Section 8.7 Follow-up of adverse events and serious adverse events and assessment of outcome (Penultimate paragraph)

All SAEs will be reported within 24 hours by the investigator to GSK Biologicals, to the PATH Focal Point, to the local safety monitor and to the WHO ~~monitor~~ *study contact for SAE reporting*. The local safety monitor will transmit the information to the ~~DSMB~~ *IDMC* according to the operating procedures of the ~~DSMB~~ *IDMC*. In addition, the local safety monitor will also provide a listing of all systemic grade 3 symptoms suspected to be related to vaccination *and any case of diphtheria, tetanus, pertussis, hepatitis B, invasive diseases due to Haemophilus influenzae type b, measles, yellow fever or Neisseria meningitidis of serogroup A and C* to the ~~DSMB~~ *IDMC* on a ~~bi-monthly~~ *regular* basis *according to the Charter for the IDMC.*

Section 8.8
Since reporting will be to the local safety monitor (who will transmit reports to WHO and IDMC) as well as to GSK Biologicals, PATH, the headings and subheadings and text have changed as follows:

8.8 Prompt reporting of serious adverse events to GSK Biologicals, *WHO,* ~~and~~ PATH *and the local safety monitor*

8.8.1 Time frames for submitting serious adverse events to GSK Biologicals, *WHO,* ~~and~~ PATH *and the local safety monitor*

Section 8.8.1 **(1st and 2nd paragraphs)**

SAEs will be reported promptly to GSK Biologicals, ***WHO,*** and PATH once the investigator determines that the event meets the protocol definition of an SAE. The investigator or designate will ~~fax~~ ***transmit*** the SAE reports to GSK Biologicals' Study Contact for Serious Adverse Event Reporting***, to the WHO study contact for SAE reporting,*** and to PATH Focal Point within 24 hours of his/her becoming aware of these events. Additional or follow-up information relating to the initial SAE report is also to be reported to the GSK Biologicals' Study Contact for Serious Adverse Event Reporting***, to the WHO study contact***

for SAE reporting, and to PATH Focal Point within 24 hours.

In addition to the direct reporting of any SAE and any subsequent information by the investigator or its designate to GSK Biologicals and PATH, the investigator will also report the SAE and subsequent information to the local safety monitor for transmission to the ***IDMC*** (see section 5.1.3) ~~and to the WHO monitor~~**.**

8.8.2 Completion and transmission of serious adverse events to GSK Biologicals, *WHO,* ~~and~~ PATH *and the local safety monitor*

Section 8.8.2 (1st paragraph and WHO study contact for reporting SAEs amended as follows)

Once an investigator becomes aware that a SAE has occurred in a study subject, she/he will report the information to GSK Biologicals**,** ***WHO*** and PATH within 24 hours as outlined in Section 8.8.1. The SAE Report Form will always be completed as thoroughly as possible with all available details of the event, signed by the investigator (or designee), and forwarded to GSK Biologicals**,** ***WHO*** and PATH within the designated time frames. If the investigator does not have all information regarding an SAE, he/she will not wait to receive additional information before notifying GSK Biologicals**,** ***WHO*** and PATH of the event and completing the form. The form will be updated when additional information is received and forwarded to GSK Biologicals**,** ***WHO*** and PATH within 24 hours as outlined in Section 8.8.1.

~~Yuppaporn Wattanagoon~~
~~M121 WHO/HTP/IVR/POP~~
~~Avenue Appia 20~~
~~CH-1211 GENEVE 27~~
~~Switzerland~~
~~Tel. +41 22 791 15 01~~
~~Email: wattanagoony@who.int~~

Dr. Zarifah Reed
WHO/ VAB/IVR/POP
20 Avenue Appia
CH-1211 GENEVE 27
Switzerland
Tel. +41 22 791 4760
Fax. +41 22 791 4860
Email: reedz@who.int

Section 10.1 Co-primary endpoints and Synopsis (6th bullet point amended by addition of bold italic text)
Anti-diphtheria concentration ≥ 0.1 IU/ml ***by Elisa and, if negative, ≥ 0.016 IU/ml by Vero-cell*** (seroprotection)

Section 10.2 Secondary endpoints and Synopsis
Pre-primary vaccination endpoints added, 11th bullet point of Antibody

persistence endpoints amended and Indirect pre vaccination status endpoints deleted as follows:

Pre-primary vaccination
In all subjects, just prior to the administration of the 1st dose of the primary vaccination

- ***SBA-MenA titer ≥ 1:8 (seropositivity)***
- ***SBA-MenC titer ≥ 1:8 (seropositivity)***
- ***Anti-PSA concentration ≥ 0.3 µg/ml (seropositivity)***
- ***Anti-PSC concentration ≥ 0.3 µg/ml (seropositivity)***
- ***Anti-PSA concentration ≥ 2 µg/ml***
- ***Anti-PSC concentration ≥ 2 µg/ml***
- ***Anti-diphtheria concentration ≥ 0.1 IU/ml (seroprotection)***
- ***Anti-HBs concentration ≥10 mIU/ml (seroprotection)***
- ***Anti-BP concentration ≥ 15 EL.U/ml (seropositivity)***

Concentration/titers of SBA-MenA, SBA-MenC, anti-PSA, anti-PSC, anti-HBs, anti-diphtheria and anti-BP antibodies

Antibody persistence (11th bullet point amended)

- Anti-diphtheria concentration ≥ 0.1 IU/ml ***by ELISA and, if negative, ≥ 0.016 IU/ml by Vero-cell*** (seroprotection)

~~**Indirect pre vaccination status through assessment of the mother:**~~
~~*Just prior to the administration of the first vaccine dose, in each mother.*~~

- ~~SBA-MenA titer ≥ 1:8 and titer~~
- ~~SBA-MenC titer ≥ 1:8 and titer~~
- ~~Anti-PSA and anti-PSC seropositivity and concentration~~

Section 10.3 Estimated sample size (1st sentence, Table 10-1 and Table 10-2)

The target sample size is ~~260~~ ***280*** enrolled subjects to reach 220 evaluable subjects in the ATP cohort for immunogenicity (110 evaluable subjects in each group).

Table 10.1: Power for criterion 1… Missing cut-off for SBA-MenC and SBA-MenA added in bold italics in Endpoint column as follows:

SBA-MenC = ***1:8***

SBA-MenA = ***1:8***

Table 10.2: Power for criterion 2… Cut-off for anti-diphtheria in Endpoint column and footnote amended in bold italics as follows:

Anti-diphtheria = 0.1 IU/ml ***by ELISA and,***

if negative, ≥ 0.016 IU/ml by Vero-cell

References: MenAC-Hib-001 for % of anti-PRP, Hib-065 for % of anti-tetanus, anti-diphtheria ***(by ELISA only)*** and anti-HBs, DTPw-HBV-033 for s.d. of log10 (concentration) for anti-BP.

Section 10.4 Study cohorts to be evaluated

This section was updated to reflect GSK Biologicals change of use of the **Total cohort** to the **Total vaccinated cohort.**
Changes were as follows with new text in bold italics:

A total of 6 cohorts are defined for the purpose of analysis.

- Three cohorts that will be used for the analysis of the primary vaccination course:
  - the ~~Primary~~ Total ***vaccinated*** cohort
  - the ~~Primary~~ ATP cohort for safety
  - the ~~Primary~~ ATP cohort for immunogenicity (used for the analysis of the co-primary objectives of immunogenicity)

- Three cohorts that will be used for the analysis of the booster phase:
  - the ~~Booster~~ Total ***vaccinated*** cohort
  - the ~~Booster~~ ATP cohort for safety
  - the ATP cohort for immune memory

The ~~Primary~~ Total ***vaccinated*** **cohort *(Primary vaccination)***

~~The primary total cohort will include all subjects vaccinated in the primary phase of the study. The analysis of an endpoint based on the primary total cohort will include all subjects vaccinated in the primary phase for whom data for the endpoint are available.~~
The Total vaccinated cohort will include all subjects vaccinated in the primary phase of the study. For the Total analysis of safety, this will include all subjects vaccinated in the primary phase of the study with at least one vaccine administration documented in the primary phase. For the Total analysis of immunogenicity / efficacy, this will include subjects vaccinated in the primary phase of the study for whom data concerning immunogenicity/efficacy endpoint measures are available. The Total vaccinated cohort analysis will be performed per treatment actually administered.

~~Primary~~ According to protocol (ATP) cohort for safety *(Primary vaccination)*

The ~~Primary~~ According to protocol (ATP) cohort for safety will include all subjects:

~~Primary~~ According to protocol (ATP) cohort for immunogenicity *(Primary vaccination)*

The ~~Primary~~ According to protocol (ATP) cohort for immunogenicity will include all evaluable subjects (i.e. those meeting all eligibility criteria, complying with the procedures defined in the protocol, and with no elimination criteria during the study) from the ~~Primary~~ ATP cohort for safety for whom assay results are available for antibodies against at least one study vaccine antigen component at Visit 4.

The ~~Booster~~ Total ***vaccinated*** ~~**Cohort**~~ ***cohort (Booster vaccination)***

~~The Booster total cohort will include all subjects vaccinated in the booster phase of the study. The analysis of an endpoint based on the Booster total cohort will include all subjects vaccinated in the booster phase for whom data for the endpoint are available.~~

The Total vaccinated cohort will include all subjects vaccinated in the booster phase of the study. For the Total analysis of safety, this will include all subjects vaccinated in the booster phase of the study with at least one vaccine administration documented in the booster phase. For the Total analysis of immunogenicity/efficacity, this will include subjects vaccinated in the booster phase of the study for whom data concerning immunogenicity / efficacy endpoint measures are available. The Total vaccinated cohort analysis will be performed per treatment actually administered.

~~Booster~~ According To Protocol (ATP) cohort for safety *(Booster vaccination)*

The ~~Booster~~ ATP cohort for safety will include all subjects from the ~~Primary~~ ATP cohort ***(primary vaccination)*** for safety

Section 10.5 Derived and transformed data (whole section amended)

~~For a given subject and cohort and the analysis of a given measurement, missing or non-evaluable measurements will not be replaced. Therefore, an analysis will exclude subjects with missing or non-evaluable measurements.~~

~~For immunogenicity data, the antibody concentrations/titers below the cut-off of the assay will be given an arbitrary value of half the cut-off for the purpose of the GMC/GMT calculation.~~

Immunogenicity

For a given subject cohort and the analysis of a given measurement, missing or unevaluable measurements will not be replaced. Therefore an analysis will exclude subjects with missing or unevaluable measurements.

- ***The cut-off value is defined by the laboratory before the analysis and is described in Section 5.5.2.***
- ***A seronegative subject is a subject whose titer or concentration is below the cut-off value.***
- ***A seropositive subject is a subject whose titer or concentration is greater than or equal to the cut-off value.***
- ***Antibody concentrations or titers below the cut-off of the assay will be given an arbitrary value of half the cut-off for the purpose of GMC or GMT calculation.***

Other

Subjects who missed reporting symptoms (solicited or unsolicited or concomitant medications) will be treated as subjects without symptoms (solicited or unsolicited or concomitant medications, respectively).

Section 10.6.2 Analysis of Immunogenicity (1st paragraph)

The analysis of immunogenicity will be based on the ~~Primary~~ ATP cohort for immunogenicity. If, ***for any vaccine group,*** the percentage of enrolled subjects ***with serological results*** excluded from this ATP cohort is more than 5%, a second analysis based on the ~~Primary~~ Total ***vaccinated*** cohort will be performed to complement the ATP analysis.

Section 10.6.3 Analysis of safety (1st paragraph)

The primary analysis will be based on the ~~Primary~~ Total ***vaccinated*** cohort. If the percentage of enrolled subjects excluded from the ~~Primary~~ ATP cohort for safety is more than 5%, a second analysis based on this ATP cohort will be performed to complement the ~~Primary~~ Total ***vaccinated*** cohort analysis.

Section 10.7.1 Analysis of immunogenicity (1st paragraph)

The analysis of antibody persistence and the analysis of immune memory will be

respectively based on the ~~Booster~~ ATP cohort for safety and the ATP cohort for immune memory. If***, for any vaccine group,*** the percentage of enrolled subjects ***with serological results*** excluded from an ATP cohort is more than 5%, a second analysis based on the ~~Booster~~ Total ***vaccinated*** cohort will be performed to complement the ATP analysis.

Section 10.7.2 Analysis of safety (1st paragraph)

All analyses will be based on the ~~Booster~~ Total ***vaccinated*** cohort. If the percentage of enrolled subjects excluded from the ~~Booster~~ ATP cohort for safety is more than 5%, a second analysis based on this ATP cohort will be performed to complement the ~~Booster~~ Total ***vaccinated*** cohort analysis.

Section 10.8 Reporting of final analyses (2nd paragraph)

A final analysis for the booster vaccination will be carried out based on all data collected up to one month after the administration of one fifth of a dose of Mencevax™ AC. ***An annex report based on the final analysis for the booster vaccination will be provided.***

References (number 26 was amended and three additional references added)

26 Enos K. Cerebrospinal meningitis in northern Ghana: the experience of the War Memorial ***Hospital, Navrongo [Report]. Ghana: Ministry of Health. 1997.***
29 Andrews N, Borrow R, Miller E. Validation of serological correlate of protection for meningococcal C conjugate vaccine by using efficacy estimates from post licensure surveillance in England. Clin Diagn Lab Immunol 2003;10:780-786.
33 Miyamura K, Nisho, S Ito A, Murata R, Kono R. Micro cell culture method for determination of diphtheria toxin and antitoxin concentrations using VERO cells: I. Studies on factors affecting the toxin and antitoxin titration. J Biol Stand 1974;2:189-201.
34 Miyamura K, Nisho ,S Ito A, Murata R, Kono R. Micro cell culture method for determination of diphtheria toxin and antitoxin concentrations using VERO cells: II. Comparison with rabbit skin method and practical application for seroepidemiological studies. J. Biol Stand 1974;2:203-209.

Appendix C Overview of the recruitment plan (5th and 6th paragraphs amended and 2 bullet points)

On the day of immunization, prior to any study procedure, the parent information sheet and informed consent forms are read and explained to the subject's parents/guardians ~~(mothers)~~ in the local language and according to the local custom and questions can be answered. Literate parents/guardians ~~(mothers)~~ are given the information sheet and IC form to read.

When agreement is given by the parent/guardian ~~(mother)~~, the IC is signed or thumbprinted (illiterates) by the parents/guardians ~~(mothers)~~ of the subject, witnessed by an independent person.

Anticipated recruitment rate is ***approximately*** ~~52~~ ***56*** subjects per month. Subjects will be recruited when they present themselves at the immunization clinics.

- Enrolment will be terminated when ~~260~~ ***280*** subjects are enrolled.

Appendix G: Vaccine supplies, packaging and accountability (Points 1, 2 and 3)

1. Vaccine supplies

- ~~390~~ ***420*** doses of Hib-MenAC vaccine in monodose vials
- ~~390~~ ***420*** doses of Hiberix™ vaccine in monodose vials
- ~~780~~ ***1260*** doses of Tritanrix™-HepB vaccine in monodose vials
- ~~260~~ ***280*** doses of Mencevax™ AC
- ~~520~~ ***560*** doses of 0.5 ml diluent for the Mencevax™ AC vaccine

2. Vaccine packaging

The vaccines will be packed in labelled boxes. ***In order to ensure the double blind of the study, all vaccine boxes will be identical (size and appearance), despite the different number of vials needed for reconstitution of the vaccines. Only the vaccination team will open the boxes for reconstitution and administration of the vaccine so only the vaccination team will know which vaccine is contained in the individual boxes (see section 6.4).*** The box label will contain, as a minimum, the following information: study number, subject number, lot number (or numbers, when double-blind), instructions for vaccine administration.

3 Vaccine accountability

The investigator or pharmacist must sign a statement that he/she has received the clinical supplies for the study. At all times the figures on supplied, used and remaining vaccine doses should match. ***All used vials should be placed in sealed envelopes by the vaccination team in order to maintain the blinding of the study.*** ~~At~~ ***After*** the end of the study ***and unblinding***, it must be possible to

reconcile delivery records with those of used and unused stocks. An explanation must be given of any discrepancies.

In addition, minor typographical errors have also been corrected throughout the protocol. The abbreviation list has been amended to include IDMC and Vero (African monkey kidney cells) and DSMB has been deleted. DSMB has also been changed to IDMC in definition of Local Safety Monitor in the Glossary of Terms.

PARENT INFORMATION SHEET AND INFORMED CONSENT

This document has also been amended so that it is consistent with the protocol. In addition, throughout the document, [your] child has been changed to [your] child/ward.

GlaxoSmithKline Biologicals
Rue de l'Institut 89
1330 Rixensart, Belgium

Protocol Amendment Approval
Study Drug
Hib-MenAC
Protocol Number
759346/009 (DTPwHB/HIBMenACTT009)
5 June 2003 and 02 October 2003)

Protocol Title: A phase II, double blind, randomized, controlled study to evaluate the immunogenicity, reactogenicity and safety of GlaxoSmithKline (GSK) Biologicals' Hib-MenAC vaccine, extemporaneously mixed with GSK Biologicals' Tritanrix™-HepB, as compared to GSK Biologicals' Hiberix™ vaccine, extemporaneously mixed with GSK Biologicals' Tritanrix™-HepB, when administered intramuscularly in infants at 6, 10 and 14 weeks of age.

Amendment 3
05 January 2004

Coordinating Author: Nikola Bülow
Rationale/background for changes:
As the collaboration with MVP on this study was cancelled, PATH does not longer act as sponsor for this study.

For the results of study 759346/001 (DTPwHB/HibMenACTT001) we refer to the new IB for Tritanrix™-HepB/Hib-MenAC (combined Diphtheria-Tetanus-whole cell Pertussis-Hepatitis B, *Haemophilus Influenzae* type B and *Neisseria meningitidis* serogroup A and C-Tetanus toxoid conjugate vaccine Investigator Brochure, 1st Edition, January 2004).

The following items were amended on 05 January 2004:

Reference to PATH and MVP was deleted from the entire protocol.

Section 1.2 GSK Biologicals' combined *H. influenzae* type b - *N. meningitidis* polysaccharide A, C (Hib-MenAC) conjugated vaccine (paragraph 4: last sentence was deleted, wording of paragraph 5 was changed)

~~***See Appendix I for a summary of clinical data of this study, including a preliminary report on the Serious Adverse Events reported during that study.***~~

Pre-clinical and clinical data generated with GSK Biologicals' ~~meningococcal conjugate vaccines are available in the *Neisseria meningitidis* conjugate vaccines Investigator Brochure (*Neisseria meningitidis* A, C and Y conjugate vaccines, 3rd edition, September 2001).~~ ~~***An updated edition of this Investigator Brochure (which will include the data presented in AppendixI) is under preparation.***~~ ***Tritanrix™-HepB/Hib-MenAC vaccine are available in the combined Diphtheria-Tetanus-whole cell Pertussis-Hepatitis B, Haemophilus Influenzae type B and Neisseria meningitidis serogroup A and C-Tetanus toxoid conjugate vaccine Investigator Brochure (DTPw-HBV-Hib-MenAC, 1st Edition, January 2004).***

Section 4.1 Number of subjects (last sentence of 1st paragraph)

The study will be conducted at the Navrongo Health Research Center in Ghana (War ***M***emorial Hospital and ***2*** health centres of Navrongo health district).

Section 8.8.2 Completion and transmission of serious adverse event reports to GSK Biologicals, *WHO and the local safety monitor* (1st sentence of 1st paragraph)

Once an investigator becomes aware that a SAE has occurred in a study subject, she/he will report the information to GSK Biologicals and WHO within 24 hours ***and to the local safety monitor*** as outlined in Section 8.8.1.

Appendix I: Summary of Clinical Results from Study 759346/001 (DTPwHB/HibMenACTT001)

The entire section was removed from the protocol as we now refer to the new IB for DTPw-HBV/Hib-MenAC.

GlaxoSmithKline Biologicals
Rue de l'Institut 89
1330 Rixensart, Belgium

Protocol Amendment Approval

Study Drug

Hib-MenAC

Protocol Number

759346/009 (DTPwHB/HIBMenACTT009)

5 June 2003, 02 October 2003

05 January 2004 and 27 January 2004)

**Protocol Title: Protocol Title:** A phase II, double blind, randomized, controlled study to evaluate the immunogenicity, reactogenicity and safety of GlaxoSmithKline (GSK) Biologicals' Hib-MenAC vaccine, extemporaneously mixed with GSK Biologicals' Tritanrix™-HepB, as compared to GSK Biologicals' Hiberix™ vaccine, extemporaneously mixed with GSK Biologicals' Tritanrix™-HepB, when administered intramuscularly in infants at 6, 10 and 14 weeks of age.

Amendment 4

27 January 2004

Coordinating Author: Catherine Murphy<br>***Rationale/background for changes:***<br>Due to a lack of funding, the mucosal study component of the ancillary study was removed .<br><br>***The following items were amended on 27 January 2004:***
The following names and items have been deleted from the entire protocol:<br>The School of Medical Sciences at the University of Bristol, United Kingdom (page 2, Section 5.1, Section 5.2 fourth paragraph and Appendix H); the Co-Investigator Dr Robert Heyderman (page 2 and Appendix H); and collection of saliva sample (Section 5.3 Outline of study procedures Table 1 and footnotes; Section 5.4 visits 1, 2, 4, 5 and 6; Section 5.5.1 See Appendix H; Section 5.5.2 last sentence; and Appendix D last sentence).

Section 5.3, Table 5-1 (Informed consent) and Section 5.4 (Detailed description of study stages/visits – Collection of throat swabs…): At Visit 2, 4, 5, and 6 added:
# any parent/guardian accompanying the child at visits subsequent to the first visit who has not already signed an Informed Consent for this study will have to sign an Informed Consent prior to providing any throat swab

Study Contact for SAE reporting at WHO, abbreviation VAB changed to IVB (Fourth page and Pages 4 and 78 Main Protocol):
From: ***WHO/~~VAB~~/IVR/POP*** To: **WHO/IVB/IVR/POP**

Appendices D and E

The following changes and/or additions were made to each appendix:

Attention changed from Mr. Emmanuel Dael/Mr. Philippe Hoebregts to ***Biospecimen Reception.***
An e-mail address and additional telephone numbers have been added and a new ***fax number*** provided.

From: ~~Attention Mr. Emmanuel Dael/Mr. Philippe Hoebregts~~
~~Clinical Immunology~~
R & D Department/Building 44
Rue de l'Institut, 89
B-1330 Rixensart – Belgium
Telephone: +32-2-656 8949/+~~32-2-656 9718~~
~~Fax: +32-2-656~~ ***9144*** **(Amended 05 June 2003)**

To:
Attention: Biospecimen Reception
R & D Department/Building 44
Rue de l'Institut, 89
B-1330 Rixensart – Belgium
Telephone: +32-2-656 8949 or +32-2-656 6130
or +32-2-656 8549 or +32-2-656 6108
Fax: +32-2-656 6052
E-mail: rix.ugbiospecimen-reception@gskbio.com

Appendix H<br>The Ancillary Study Protocol has been amended.<br>The title of this Appendix H and the Ancillary Study Protocol (within) are changed:<br>From: Meningococcal carriage and mucosal immunity to *Neisseria meningitidis* in the African meningitis belt<br>To ***Meningococcal carriage in the African meningitis belt.***
Please Note: Appendix I was deleted following the approval/availability of the IB, therefore, Appendix J sequentially became Appendix I.
The Parent Information Sheet/IC:<br>(Title page and Approval Section): The School of Medical Sciences, University of Bristol was deleted.<br>The Amendment date, 27 January 2004, was added : Introduction (page1); Study Participation, seventh bullet; Confidential and source document review (page 7); Compensation (page 10, above signature); and Informed Consent pages 11 and 12, and Informed Consent page 13).<br>In the Study Overview, paragraph 5, the following sentence was changed<br>From: The blood samples taken during the study will help to see whether the vaccine has induced antibodies (antibodies are particles ***acting as soldiers to*** protect against diseases) in the blood of your child***/ward*** but it is important that the vaccine also ***induces*** antibodies that will be present in your child's***/ward's*** nose/throat<br>To: The blood samples taken during the study will help to see whether the vaccine has induced antibodies (antibodies are particles ***acting as soldiers to*** protect against diseases) in the blood of your child***/ward*** but it is important that the vaccine also ***prevents the bacteria from staying in the throat.***

GlaxoSmithKline Biologicals
Rue de l'Institut 89
1330 Rixensart, Belgium

Protocol Amendment Approval

Study Drug

Hib-MenAC

Protocol Number

759346/009 (DTPwHB/HIBMenACTT009)

5 June 2003, 02 October 2003, 05 January 2004, 27 January 2004 and 2 April 2004)

Protocol Title: Protocol Title: A phase II, double blind, randomized, controlled study to evaluate the immunogenicity, reactogenicity and safety of GlaxoSmithKline (GSK) Biologicals' Hib-MenAC vaccine, extemporaneously mixed with GSK Biologicals' Tritanrix™-HepB, as compared to GSK Biologicals' Hiberix™ vaccine, extemporaneously mixed with GSK Biologicals' Tritanrix™-HepB, when administered intramuscularly in infants at 6, 10 and 14 weeks of age.

Amendment 5

2 April 2004

Coordinating Author: Sheila Woods

Rationale/background for changes:

The 1st meeting of the IDMC recommended the following changes to the protocol:

- Considering the problem of assessment of birth weight in the field (birth weight is taken in less than 1/2 of the babies), amend eligibility criteria to exclude babies with low birth-weight/malnutrition
- Include the use of verbal autopsy questionnaires to improve the mortality investigations in case of death
- Clarify/improve the flow of communication for SAEs and non serious AEs suggesting lack of efficacy and non-serious vaccine-related grade 3 AEs;

The following items were amended on 2 April 2004:

The back-up study contact for reporting SAEs at GSK Biologicals is changed from ~~Marc Ceuppens~~ to the ***GSK Biologicals Clinical Safety Physician***; the protocol has been amended accordingly.

The email address of the study monitor Cornelia Bevilacqua has changed from ~~cornelia_bevilacqua@yahoo.fr~~ to ***corneliabevilacqua@usa.net*** and the fax number of the study monitor Valérie Marichal has changed from +32 2 656.~~9072~~ to +32 2 656.***8133***; the protocol has been amended accordingly.

Section 1.4: the **'Coordination with the EPI'** section was amended as follows: The vaccine trial will be conducted at ~~**three**~~ ***several*** EPI clinics in the Kassena-Nankana district where routine immunization is taking place**.**~~:~~

~~**- KNE Health Centre (serving the East sub district)**~~

~~**- Biu Health Centre (serving the South sub district)**~~

~~**- War Memorial Hospital (serving Navrongo central)**~~

Section 4.1: 2nd sentence amended as follows:

The study will be conducted at the Navrongo Health Research Center in Ghana (War Memorial Hospital and ~~2~~ ***several*** health centres of Navrongo health district).

Section 4.2: the following inclusion criterion deleted:

- ~~Birth weight > 2 kg.~~

Section 4.3: exclusion criteria amended as follows:

- Major congenital defects~~,~~ ~~malnutrition~~ or serious chronic illness.
- ***Babies for which birth weight is <2 kg (if known), and/or malnutrition at visit 1.***

Section 5.1.3: Role of the IDMC section
3rd paragraph, 2nd bullet changed as follows:

- systemic grade 3 adverse events (AEs) related to vaccination, serious adverse events and adverse events suggesting a lack of efficacy of the vaccine occurring during the study (transmitted ***through GSK Biologicals for SAEs and*** through the Local safety Monitor ***for the other adverse events***)

4th paragraph: 2nd and 3rd sentences changed as follows:

All SAEs, including death, will be reported by the Principal Investigator to the ~~***IDMC***~~ ~~(via the local safety monitor), to the Manager of Clinical Safety Vaccines at GSK Biologicals, to WHO and to the WHO safety monitor. All SAEs will be provided on an "as received" basis to the Board.~~ ***local safety monitor, to the Manager of Clinical Safety Vaccines at GSK Biologicals, to WHO and to the WHO safety monitor. GSK Biologicals will be responsible to provide all information related to the SAEs to the IDMC according to the IDMC Charter.***

Section 5.1.3: Role of Local Safety Monitor section.
Heading and first paragraph changed as follows:

Reporting to IDMC **– Role of Local Safety Monitor**

A Local Safety Monitor (LSM) will act independently from the investigational team. The overall role of the Local Safety Monitor (LSM), who will be an experienced clinician based in-country, will be to support the clinical investigators and to act as a link between the investigators and the **IDMC**. All systemic grade 3 AEs probably or suspected to be related to vaccination, all SAEs and all AEs suggesting a lack of efficacy of the vaccine will be reported to him/her ~~in his/her capacity as a liaison to the **IDMC**~~. His/her involvement…

Following added as 2nd bullet to bulleted list under "The LSM's role will include:"

- ***provide the IDMC and the medical monitor of GSK Biologicals with a blinded listing of non-serious systemic grade 3 symptoms (solicited and unsolicited) that are related with vaccination, and of non-serious adverse events suggesting a lack of efficacy of the vaccine, this according to the IDMC Charter (note: GSK Biologicals will send all information related to serious adverse events to the IDMC according to the IDMC Charter).***

Section 8.7:
5th paragraph: Addition of following sentence at end of:
In the case of a death, supplementary information will be gained using the verbal autopsy technique. This will be conducted according to previously published methods and detailed in the SOPs on file with the investigators. The Verbal Autopsy Questionnaire will be completed and transmitted by the investigator to GSK Biologicals, WHO and the Local Safety Monitor, in addition to the SAE report, irrespective of relationship to vaccination and whether a written autopsy was performed or not. The Standard Verbal Autopsy Questionnaire will not replace a written autopsy.

7th Paragraph: Amended (including dividing into 2 paragraphs) as follows:
All SAEs will be reported within 24 hours by the investigator to GSK Biologicals ***safety department***, to the local safety monitor and to the WHO *study contact for SAE reporting*. ~~The local safety monitor~~ ***GSK Biologicals*** will transmit the information to the *IDMC* according to the operating procedures of the *IDMC*.

In addition, the ~~local safety monitor~~ ***investigator*** will also provide a listing of all systemic grade 3 symptoms suspected to be related to vaccination and any case of diphtheria, tetanus, pertussis, hepatitis B, invasive diseases due to *Haemophilus influenzae* type b, measles, yellow fever or *Neisseria meningitidis* of serogroup A and C ***to the LSM. The Local Safety Monitor will send the listing and information related to the non-serious grade 3 systemic symptoms related with vaccination and non-serious adverse events suggesting a lack of efficacy of the vaccine*** to the IDMC ***and the GSK medical monitor (the GSK medical monitor will forward the information to the other members of the Steering Committee)*** ~~on a regular basis~~ **according to the Charter for the IDMC**.

Section 8.8.1:
1st paragraph: 1st sentence amended as follows:
SAEs will be reported promptly to ***the LSM,*** GSK Biologicals and WHO once the investigator determines that the event meets the protocol definition of an SAE.
1st paragraph: 3rd sentence amended as follows:
Additional or follow-up information relating to the initial SAE report***, including completed verbal autopsy questionnaire in case of death,*** is also to be reported to the GSK Biologicals' Study Contact for Serious Adverse Event Reporting and ***to*** the WHO study contact for SAE reporting within 24 hours.**,**

2nd paragraph: Amended as follows:
~~In addition to the direct reporting of any SAE and any subsequent information by the investigator or its designate to~~ GSK Biologicals ~~and WHO, the investigator will also report the SAE and subsequent information to the local safety monitor for transmission~~ ***will transmit the SAEs to the IDMC on a regular basis according to the Charter for the IDMC*** (see section 5.1.3).

Appendix H; protocol for ancillary study

Section II 1. 3rd paragraph amended in line with the main protocol.

Section II 2. and 3.Inclusion and exclusion criteria have been amended in line with the main protocol.

Section III 3.2 and 3.3 amended in line with the main protocol.

Section IV 2. Amended in line with main protocol

The Parent Information Sheet/IC: There were no changes to this document for this amendment.

Various typographical errors were also corrected.

GlaxoSmithKline Biologicals
Rue de l'Institut 89
1330 Rixensart, Belgium

Protocol Modification Approval

Study Drug

Hib-MenAC

Protocol Number

759346/009 (DTPwHB/HIBMenACTT009)

5 June 2003, 02 October 2003, 05 January 2004,
27 January 2004 and 2 April 2004, modified 07 July 2004)

**Protocol Title: Protocol Title:** A phase II, double blind, randomized, controlled study to evaluate the immunogenicity, reactogenicity and safety of GlaxoSmithKline (GSK) Biologicals' Hib-MenAC vaccine, extemporaneously mixed with GSK Biologicals' Tritanrix™-HepB, as compared to GSK Biologicals' Hiberix™ vaccine, extemporaneously mixed with GSK Biologicals' Tritanrix™-HepB, when administered intramuscularly in infants at 6, 10 and 14 weeks of age.

Modification 1
07 July 2004

Coordinating Author: Nikola Bulow

Rationale/background for changes:

Change in study personnel: The back-up of the local safety monitor, Dr Rita Baiden, was replaced by Dr. Frank Baiden.

The following items were modified throughout the protocol on 07 July 2004:

Back-up Local Safety Monitor
~~Dr. Rita Baiden~~ ***Dr. Frank Baiden***
Navrongo Health Research Center,
Ministry of Health, PO Box 114, Navrongo, Ghana
Tel: + 233 742 22310/ 22380
Fax: + 233 742 22320
e-mail: ~~radomako-bamfi@navrongo.mimcom.net~~***fbaiden@navrongo.mimcom.net***

GlaxoSmithKline Biologicals
Rue de l'Institut 89
1330 Rixensart, Belgium

Protocol Amendment Approval
Study Drug
Hib-MenAC
Protocol Number
759346/009 (DTPwHB/HIBMenACTT009)
104430 (DTPwHB/HIBMenACTT023 BST:009)
5 June 2003, 02 October 2003, 05 January 2004,
27 January 2004 and 2 April 2004, modified 07 July 2004, amended 14 January 2005)

**Protocol Title: Protocol Title:** A phase II, double blind, randomized, controlled study to evaluate the immunogenicity, reactogenicity and safety of GlaxoSmithKline (GSK) Biologicals' Hib-MenAC vaccine, extemporaneously mixed with GSK Biologicals' Tritanrix™-HepB, as compared to GSK Biologicals' Hiberix™ vaccine, extemporaneously mixed with GSK Biologicals' Tritanrix™-HepB, when administered intramuscularly in infants at 6, 10 and 14 weeks of age.

Amendment 6
14 January 2005

Coordinatin Author: Nikola Bulow

Rationale/background for changes:

To allow the analysis of the primary phase (up to Visit 4) on cleaned data, the database will be divided into two separate parts and therefore a new e-track study number and abbreviated title have been allocated for the booster phase of the study; the conclusion of the primary phase has been fixed at the end of Visit 4 (additional page on the CRF). Recording of concomitant medication/vaccination will be done according to the initial protocol (see Section 6.8, i.e. throughout the study, from Visit 1 to Visit 7), but due to the separation of the data base in two parts, clarification on how this will be handled has been added to the protocol. It has to be noted that the informed consent signed by the subject's parents/

guardians is referring to the e-Track study number 759346/009; parents/guardians of the subjects will not be asked to re-consent for adding the second e-Track number and abbreviated title 104430 (DTPwHB/HIBMenACTT023 BST:009) as this change will not affect any aspect of the safety of the subject, nor the study procedures the subject is involved in or the objectives of the study.

The following items were amended on 14 January 2005:

Throughout the protocol the new e-Track number and abbreviated title 104430 (DTPwHB/HIBMenACTT023 BST:009) for the booster phase of the study was added.

Section 4.1 Number of subjects (one paragraph was added at the end of the section)

If at the time of the initiation of the booster phase any parent/guardian declines participation of his/her child, refusal will be documented as instructed in the "subject tracking document" provided by GSK Biologicals. A copy of the completed tracking document will be forwarded to GSK Biologicals' Study Monitor. The information will be entered in the GSK Biologicals' clinical database for use in identification of any safety issue that may have prevented a subject's participation.

Section 5.3 Outline of study procedures (Table 5-1)

The heading "***Primary Phase (759346/009)***" was added for Visits 1 to 4 and the heading "***Booster Phase (104430)***" was added for Visits 5 to 7. A double-line border was added between Visit 4 and Visit 5 to indicate the conclusion of the Primary Phase of the study. A row for the "***Study Conclusion of the Primary Phase***" was added and will be checked at Visit 4. A row for "***Retrospective recording of relevant concomitant medication/vaccination since Visit 4***[###]" was added and will be checked at Visit 5. The last row "Study conclusion" was changed to "Study conclusion ***of Booster Phase***".
[###]***Relevant medication/treatment will include meningococcal vaccine, any investigational or non-registered product (drug or vaccine), any immunosuppressant(s) or other immune-modifying drug(s), immunoglobulins and/or any blood product(s).***

Section 5.4 Detailed description of study stages/visits

Visit 4: The following bullet point was added:

- ***Study conclusion of the primary phase***

Visit 5: The following bullet points were added:

- ***If at the time of the initiation of the booster phase any parent/guardian declines participation of his/her child, refusal will be documented as instructed in the "subject tracking document" provided by GSK Biologicals. A copy of the completed tracking document will be forwarded to GSK Biologicals' Study Monitor. The information will be entered in the GSK Biologicals' clinical database for use in identification of any safety issue that may have prevented a subject's participation.***
- ***Retrospective recording of any relevant concomitant medication/ vaccination since the last visit according to instructions provided in Section 6.8. Relevant vaccines will include meningococcal vaccines and investigational or non registered vaccines and will be recorded with trade name, route of administration and date(s) of administration. Relevant medications/treatments will include any investigational or non-registered product(s), any immunosuppressant(s) or other immun-modifying drug(s), immunoglobulins and/or any blood products. All such medication/treatment is to be recorded with generic name of the medication (trade names are allowed for combination drugs, i.e. multi-component drugs), medical indication, total daily dose, route of administration, start and end dates of treatment.***

Section 6.8.1 Recording of concomitant medication/treatment during the primary phase of the study

This sub-heading was added to differentiate the record of medication/treatment during the primary and booster phases.

Section 6.8.2 Recording of concomitant medication/treatment during the booster phase of the study

The entire section was added:
At Visit 5, the investigator will question the parents/guardians in a RETROSPECTIVE manner if the subject received any relevant medication/treatment or vaccine in the time period since the last study visit of the primary phase of the study (Visit 4). Relevant vaccines will include meningococcal vaccines and investigational or non registered vaccines and will be recorded with trade name, route of administration and date(s) of administration. Relevant medications/ treatments will include any investigational or non-registered product(s), any immunosuppressant(s) or other immune-modifying drug(s), immunoglobulins and/or any blood products. All such medication/treatment is to be recorded with generic name of the medication (trade named are allowed for combination drugs, i.e. multi-component drugs), medical indication, total daily dose, route of administration, start and end dates of treatment.

In addition, all concomitant medication, with the exception of vitamins and/or dietary supplements, administered at ANY time during the period starting with administration of each dose and ending one month (minimum 30 days) after each dose are to be recorded with generic name of the medication (trade names are allowed for combination drugs, i.e., multi-component drugs), medical indication, total daily dose, route of administration, start and end dates of treatment.

Any treatments and/or medications specifically contraindicated, e.g., any immunoglobulins, other blood products and any immune modifying drugs administered at any time during the study period are to be recorded with generic name of the medication (trade names are allowed for combination drugs only), medical indication, total daily dose, route of administration, start and end dates of treatment. Refer to Section 4.4.

Any vaccine not foreseen in the study protocol administered throughout the study period are to be recorded with trade name, route of administration and date(s) of administration, with the exception of OPV that can be given throughout the study. Refer to Section 4.4.

Any concomitant medication administered prophylactically in anticipation of reaction to the vaccination must be recorded in the CRF with generic name of the medication (trade names are allowed for combination drugs only), total daily dose, route of administration, start and end dates of treatment and coded as 'Prophylactic'.

Concomitant medication administered for the treatment of an AE or SAE must be recorded in the CRF with generic name of the medication (trade names are allowed for combination drugs only), medical indication (including which AE/SAE), total daily dose, route of administration, start and end dates of treatment. Refer to Section 8.2 for definition of SAE.

GlaxoSmithKline Biologicals
Rue de l'Institut 89
1330 Rixensart, Belgium

Protocol Amendment Approval

Study Drug

Hib-MenAC

Protocol Numbers

759346/009 (DTPwHB/HIBMenACTT009)
104430 (DTPwHB/HIBMenACTT023 BST:009)

Amended on: 05 June 2003, 02 October 2003, 05 January 2004, 27 January 2004, 2 April 2004 and 14 January 2005

Modified on: 7 July 2004

**Protocol Title:** A phase II, double blind, randomized, controlled study to evaluate the immunogenicity, reactogenicity and safety of GlaxoSmithKline (GSK) Biologicals' Hib-MenAC vaccine, extemporaneously mixed with GSK Biologicals' Tritanrix™-HepB, as compared to GSK Biologicals' Hiberix™ vaccine, extemporaneously mixed with GSK Biologicals' Tritanrix™-HepB, when administered intramuscularly in infants at 6, 10 and 14 weeks of age.

Amendment 7: 12 April 2006

Co-ordinating Author: Chitra Nair, Scientific Writer

Rationale/background for changes:

In the primary vaccination phase of this study, serious adverse events were reported for 11 subjects in the DTPw-HepB/Hib-MenAC group (including three deaths) and 2 subjects in the DTPw-HepB/Hib control group; none of these SAEs were related to vaccination. The pattern of SAEs was varied and reflected the medical and health conditions in the district and there were no unusual causes of death. All 10 subjects with non-fatal serious adverse events had infectious diseases commonly reported in Navrongo Health District (malaria, bronchopneumonia, respiratory tract infection, typhoid fever, enteritis, gastroenteritis). The three fatal cases were dysentery and impetigo, aspiration pneumonia, and sudden infant death syndrome, all after the first vaccine dose. After review of these safety data, the Independent Data Monitoring Committee advised the sponsor (GSK Biologicals) to conduct a retrospective follow-up of SAEs for all subjects who participated in this study since the last study visit up to when subjects are aged at least 24 months. The protocol is being amended to reflect the addition of the extended safety follow-up phase.

Additionally, contact details were updated in the relevant sections of the protocol.

An addendum to the representative subject information sheet was prepared to detail the extended safety follow-up phase.

Amended text has been included in *bold italics* in the following sections:

Title page of the protocol:

Coordinating Authors: Uma Swaminathan ***and Chitra Nair***, Scientific Writer***s***

Contributing Authors: Dr. Dominique Boutriau, ~~Associate Director, Paediatric Vaccine Development~~ ***Director, Clinical Research & Development, Meningitis vaccines***
Toufik Zahaf, Thierry Van-Effelterre ***and Christelle Durand***, Statisticians

Sponsor Information sheet:
Study Contact for Reporting of a Serious Adverse Event at GSK Biologicals
Dr. Dominique Boutriau,
~~Associate Director Paediatric Vaccine Development~~ ***Director, Clinical Research & Development, Meningitis vaccines***
GlaxoSmithKline Biologicals,
Rue de l'Institut 89, 1330 Rixensart, Belgium
Tel: +32.2.656. 9120
Fax: +32.2.656. ~~8133~~ ***8044***
e-mail: dominique.boutriau@gskbio.com

Back-up Study Contact for Reporting SAEs at GSK Biologicals
GSK Biologicals Clinical Safety Physician
Tel: +32-2-656 ***88 50***
Fax: +32-2-656 80 09***/5116***
Mobile phone for 7/7 day availability: +32 477 404 713 or ***32-(0) 474 53 48 68***
24/24 hour and 7/7 day availability

The above changes were made in all the relevant sections of the protocol.

Study design in the Synopsis and in Section 3: Study Design Overview
The following bullet points were updated and/or added:

- Study duration per subject: approximately ~~12 months (from 6-8 weeks up to 13 months of age). The total study duration will depend on recruitment~~ ***22.5 months to include the retrospective follow-up of SAE(s) up to the time that subjects are at least 24 months of age.***
- ***Retrospective follow-up of SAE(s). The parents/guardians of study subjects will be contacted by the local health staff when their child is at least 24 months of age for a follow-up of SAE(s) (i.e. any SAE(s) that occurred since the last study visit [Visit 7] up to the time that subjects are at least 24 months of age).***

The graphic schema of the study design in Section 3 was also updated to reflect the extended safety follow-up phase.

Section 5.3: Outline of study procedures

A table was added to describe the study procedures of the extended safety follow-up phase

Table 5-2: List of study procedures for the extended safety follow-up phase

	EXTENDED SAFETY FOLLOW-UP PHASE
Age	***±24 m***
Visit	
Timing	***Month 22.5***
Informed consent	•
Retrospective recording of SAE(s) since Visit 7 up to the time that subjects are aged at least 24 months	•
Study conclusion	•

Section 5.4: Detailed description of study stages/visits

The following information was added to describe the details of the extended safety follow-up phase:

Extended Safety Follow-Up Phase: Retrospective recording of SAE(s)

The parents/guardians of all study subjects will be contacted by the local health staff when their child is at least 24 months of age for a retrospective follow-up of SAE(s) (i.e. occurrence of any SAE(s) since the last study visit [Visit 7] up to the time that subjects are at least 24 months of age). Prior to initiating the study procedures for this safety follow-up phase, an informed consent will be obtained from the parents/guardians of the child after explaining the purpose of this contact.

The study personnel will contact the parents/guardians of the subjects and will question them in a RETROSPECTIVE manner if the subject has suffered any unreported SAE(s) in the period between the last study visit (study conclusion of the booster phase) and this follow-up contact; the study personnel will also consult the child health card whenever available. If SAE(s) had occurred, further investigations will be performed on the subject's hospital/health centre records (if available). Details of these SAE(s) will be reported on the SAE forms. If a death had occurred, a verbal autopsy report will be obtained whenever possible.

As subjects will be at least two years of age at this safety follow-up visit, the study staff will also remind parents/guardians that the children part of the control group

and those with sub-optimal response to the MenA and/or MenC component of the vaccines given to the DTPw-HepB/Hib-MenAC group, will be offered a dose of Mencevax™ ACWY vaccine to protect them against meningococcal diseases (serogroups A, C, W and Y).

This will be the study conclusion for the extended safety follow-up phase.

Section 8.8.2: Completion and transmission of serious adverse event reports to GSK Biologicals, WHO and the local safety monitor

**Study Contacts for Reporting SAEs**
**Medical Monitor at GSK Biologicals**
Dr. Dominique Boutriau,<br>~~Associate Director Paediatric Vaccine Development~~<br>***Director, Clinical Research & Development, Meningitis vaccines***<br>GlaxoSmithKline Biologicals,<br>Rue de l'Institut 89, 1330 Rixensart, Belgium<br>Tel: +32.2.656.9120<br>Fax: +32.2.656. ~~8133~~ ***8044***<br>e-mail: dominique.boutriau@gskbio.com
**Back-up Study Contact for Reporting SAEs at GSK Biologicals**
~~**Manager Clinical Safety Vaccines**~~
GSK Biologicals Clinical Safety Physician<br>Tel: +32-2-656 ~~87 98~~ ***88 50***<br>Fax: +32-2-656 80 09***/5116***<br>Mobile phone for 7/7 day availability: +32 477 404 713 ***or 32-(0) 474 53 48 68***<br>**24/24 hour and 7/7 day availability**

Section 9.3: Extension study
~~Not applicable.~~
There will be a retrospective follow-up of SAE(s) that occurred since the last study visit [Visit 7] up to the time that subjects are at least 24 months of age.

Section 8.5: Time period, frequency, and method of detecting adverse events and serious adverse events
There will be a retrospective follow-up of SAE(s) (i.e. occurrence of any SAE(s) since the last study visit [Visit 7] up to the time that subjects are at least 24 months

of age).

Section 10.8: Reporting of final analyses
Serious adverse events reported during the extended follow-up period (i.e. from visit 7 up to 24 months of age) will be reported in a separate annex report.

eTrack study number:	759346/009 (DTPwHB/HIBMenACTT009)<br>104430 (DTPwHB/HIBMenACTT023 BST:009)
Protocol title:	A phase II, double blind, randomized, controlled study to evaluate the immunogenicity, reactogenicity and safety of GlaxoSmithKline (GSK) Biologicals' Hib-MenAC vaccine, extemporaneously mixed with GSK Biologicals' Tritanrix™-HepB, as compared to GSK Biologicals' Hiberix™ vaccine, extemporaneously mixed with GSK Biologicals' Tritanrix™-HepB, when administered intramuscularly in infants at 6, 10 and 14 weeks of age.
Amendment number:	Amendment 7
Amendment date:	12 April 2006

Approved by (Sponsor at GSK Biologicals):

Director, Clinical Research & Development, Meningitis vaccines	Dr. Dominique Boutriau	dd-mm-yyyy

eTrack study number:	759346/009 (DTPwHB/HIBMenACTT009)<br>104430 (DTPwHB/HIBMenACTT023 BST:009)
Protocol title:	A phase II, double blind, randomized, controlled study to evaluate the immunogenicity, reactogenicity and safety of GlaxoSmithKline (GSK) Biologicals' Hib-MenAC vaccine, extemporaneously mixed with GSK Biologicals' Tritanrix™-HepB, as compared to GSK Biologicals' Hiberix™ vaccine, extemporaneously mixed with GSK Biologicals' Tritanrix™-HepB, when administered intramuscularly in infants at 6, 10 and 14 weeks of age.
Amendment number:	Amendment 7
Amendment date:	12 April 2006

Approved by (Sponsor at GSK Biologicals):

Associate Director,
Statistical Manager

	Brigitte Cheuvart	dd-mm-yyyy

eTrack study number:	759346/009 (DTPwHB/HIBMenACTT009)<br>104430 (DTPwHB/HIBMenACTT023 BST:009)
Protocol title:	A phase II, double blind, randomized, controlled study to evaluate the immunogenicity, reactogenicity and safety of GlaxoSmithKline (GSK) Biologicals' Hib-MenAC vaccine, extemporaneously mixed with GSK Biologicals' Tritanrix™-HepB, as compared to GSK Biologicals' Hiberix™ vaccine, extemporaneously mixed with GSK Biologicals' Tritanrix™-HepB, when administered intramuscularly in infants at 6, 10 and 14 weeks of age.
Amendment number:	Amendment 7
Amendment date:	12 April 2006

Approved by:

Collaborator (at WHO)	Dr. Marie-Paule Kieny	dd-mm-yyyy

eTrack study number:	759346/009 (DTPwHB/HIBMenACTT009)<br>104430 (DTPwHB/HIBMenACTT023 BST:009)
Protocol title:	A phase II, double blind, randomized, controlled study to evaluate the immunogenicity, reactogenicity and safety of GlaxoSmithKline (GSK) Biologicals' Hib-MenAC vaccine, extemporaneously mixed with GSK Biologicals' Tritanrix™-HepB, as compared to GSK Biologicals' Hiberix™ vaccine, extemporaneously mixed with GSK Biologicals' Tritanrix™-HepB, when administered intramuscularly in infants at 6, 10 and 14 weeks of age.
Amendment number:	Amendment 7
Amendment date:	12 April 2006

Approved by:

Collaborators (at the London School of Hygiene & Tropical Medicine)	Prof. Brian Greenwood	dd-mm-yyyy
	Dr. Daniel Chandramohan	dd-mm-yyyy

eTrack study number:	759346/009 (DTPwHB/HIBMenACTT009)<br>104430 (DTPwHB/HIBMenACTT023 BST:009)
Protocol title:	A phase II, double blind, randomized, controlled study to evaluate the immunogenicity, reactogenicity and safety of GlaxoSmithKline (GSK) Biologicals' Hib-MenAC vaccine, extemporaneously mixed with GSK Biologicals' Tritanrix™-HepB, as compared to GSK Biologicals' Hiberix™ vaccine, extemporaneously mixed with GSK Biologicals' Tritanrix™-HepB, when administered intramuscularly in infants at 6, 10 and 14 weeks of age.
Amendment number:	Amendment 7
Amendment date:	12 April 2006

Approved by:

Investigator	Dr. Abraham Victor Obeng Hodgson	dd-mm-yyyy

eTrack study number:	759346/009 (DTPwHB/HIBMenACTT009)<br>104430 (DTPwHB/HIBMenACTT023 BST:009)
Protocol title:	A phase II, double blind, randomized, controlled study to evaluate the immunogenicity, reactogenicity and safety of GlaxoSmithKline (GSK) Biologicals' Hib-MenAC vaccine, extemporaneously mixed with GSK Biologicals' Tritanrix™-HepB, as compared to GSK Biologicals' Hiberix™ vaccine, extemporaneously mixed with GSK Biologicals' Tritanrix™-HepB, when administered intramuscularly in infants at 6, 10 and 14 weeks of age.
Amendment number:	Amendment 7
Amendment date:	12 April 2006

Approved by:

Investigator

Dr. Gerd Pluschke ____________________ dd-mm-yyyy ____________________
